# Mechanical Network in Titin Immunoglobulin from Force Distribution Analysis

**DOI:** 10.1371/journal.pcbi.1000306

**Published:** 2009-03-13

**Authors:** Wolfram Stacklies, M. Cristina Vega, Matthias Wilmanns, Frauke Gräter

**Affiliations:** 1 CAS-MPG Partner Institute for Computational Biology (PICB), Shanghai, People's Republic of China; 2 Institut de Biologia Molecular de Barcelona (IBMB-CSIC) and Institute for Research in Biomedicine (IRB), Barcelona, Spain; 3 EMBL-Hamburg Unit, Hamburg, Gemany; 4 Bioquant BQ0031, Universität Heidelberg, Heidelberg, Germany; 5 Max-Planck Institute for Metals Research, Stuttgart, Germany; UT Southwestern Medical Center, United States of America

## Abstract

The role of mechanical force in cellular processes is increasingly revealed by single molecule experiments and simulations of force-induced transitions in proteins. How the applied force propagates within proteins determines their mechanical behavior yet remains largely unknown. We present a new method based on molecular dynamics simulations to disclose the distribution of strain in protein structures, here for the newly determined high-resolution crystal structure of I27, a titin immunoglobulin (IG) domain. We obtain a sparse, spatially connected, and highly anisotropic mechanical network. This allows us to detect load-bearing motifs composed of interstrand hydrogen bonds and hydrophobic core interactions, including parts distal to the site to which force was applied. The role of the force distribution pattern for mechanical stability is tested by *in silico* unfolding of I27 mutants. We then compare the observed force pattern to the sparse network of coevolved residues found in this family. We find a remarkable overlap, suggesting the force distribution to reflect constraints for the evolutionary design of mechanical resistance in the IG family. The force distribution analysis provides a molecular interpretation of coevolution and opens the road to the study of the mechanism of signal propagation in proteins in general.

## Author Summary

Many biological processes such as cell proliferation and signaling are guided by mechanical stress. Proteins as the molecular machinery behind these processes are reacting to or withstanding mechanical forces in specific ways. How mechanical stress propagates through proteins to induce a certain mechanical response is currently unknown. We here present a new method that detects force distribution in proteins, reminiscent of computational approaches used to engineer macroscopic structures. The method is based on molecular dynamics simulations during which we calculate changes in interatomic forces, here caused by pulling the protein. We apply this method to the extremely robust immunoglobulin domain of the muscle protein titin and obtain the mechanical network of stress-bearing elements spanning the protein scaffold. Mutations in this region were shown to result in a significant loss of mechanical stability. We then ask how the remarkable mechanical stability has been designed during evolution. To this end, we compare the force distribution network with a network of coevolved residues found in the immunoglobulin family. Both networks show a remarkable overlap, thereby suggesting that the observed stress propagation pattern reflects constraints used during evolution to render this protein mechanically robust.

## Introduction

Cellular functions such as growth, motility, and signaling are tightly coupled with mechanical forces [1]–[3]. Proteins play a pivotal role in such mechanically guided processes, as robust elements bearing cellular stress, and as mechanosensors transducing the mechanical signal into a biochemical response [4]. A fundamental question is how a protein of mechanical function has been evolutionarily designed to withstand and transmit high levels of stress. Analysis and design of macroscopic structures such as cells, organs, or implants is routinely guided by the calculation of force propagation to predict and improve mechanical response [5],[6]. However, how force propagates through the microscopic structure of proteins upon external stress is currently unknown.

Mechanical response of proteins can be directly revealed by measuring forces for protein unfolding [7]–[9], activation [2],[10], and enzymatic action [1],[11]. For different titin immunoglobulin (IG) domains and engineered variants thereof, unfolding forces have been measured and rupture mechanisms inferred [12]–[16]. These data provide important insight into the load-bearing structural motifs of IG. A more fundamental question is how mechanical load distributes through a protein. It is an obvious assumption that force propagates through the structure to parts which, being distant from the application site of the perturbation, cannot be straightforwardly inferred from unfolding forces. Currently, there is no direct way at hand to explain the frequent experimental observation of site specific changes in dynamics (see, e.g., [17]). In this study we present a new method to detect the mechanical network sustaining load within a protein from molecular dynamics simulations. We apply the force distribution analysis to I27, an IG domain of the muscle protein titin and one of the most robust protein domains known. The atomic resolution force distribution analysis relies on an accurate three dimensional picture of atomic interactions. We have determined the first high-resolution crystal structure of I27, a cornerstone for the interpretation of force spectroscopy experiments and crucial for our analysis.

The sparse mechanical network spanning the I27 structure is reminiscent of the molecular networks revealed by statistical coupling analysis on multiple sequence alignments [18],[19]. Such networks of evolutionarily coupled residues have been proposed to reflect dynamic or energetic couplings along signaling pathways in proteins [20]–[22]. A direct relation of coevolution with molecular behavior, however, remains to be found. We compare the obtained pattern of force propagation with a network of coevolved residues found in the IG domain family, and find strongly coevolved residues to play a dominant role in force distribution. We therefore suggest internal strain propagation to present a first microscopic interpretation of a coevolved network in a protein with mechanical function.

## Results

### Overall Structure of I27

**Figure 1:**
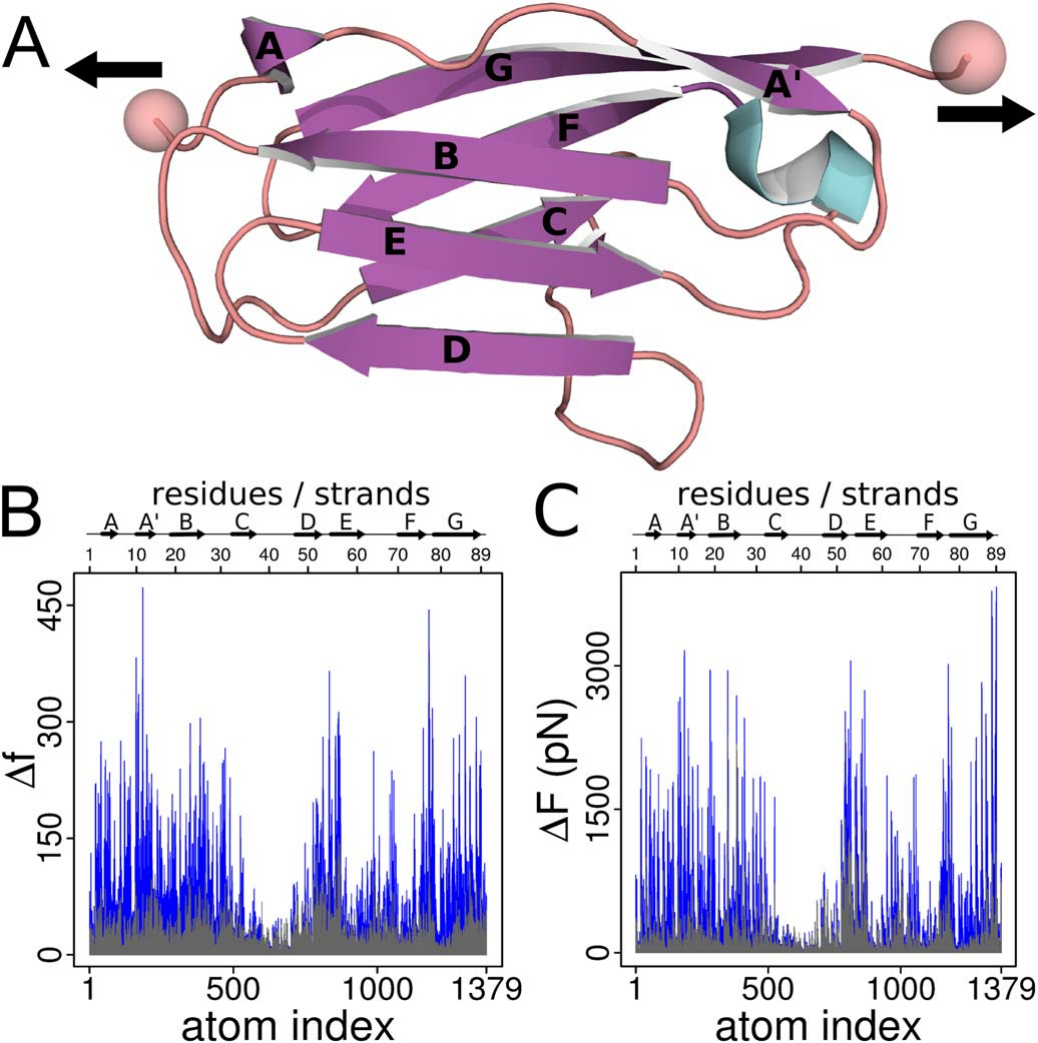
Crystal structure and force propagation in I27. (A) Crystal structure of I27 (PDB-entry 1WAA). Mechanical load during MD simulations is applied to I27 by pulling the termini with a constant force as indicated. All protein structures were plotted using PyMOL [61]. (B) Signal-to-noise ratio of atomic forces after mechanical loading and equilibration. The dimensionless normalized force signal, Δ*f*, per atom after summing over all atom pairs is measured by the difference in atomic forces between strained and relaxed state (blue), and is compared to noise, estimated from normalized differences between two sets of equilibrium trajectories (gray). (C) The raw force signal, Δ*F*, with noise plotted in gray. Comparison with the normalized signal in (B) shows that the overall force distribution pattern is not affected by normalization, see Methods for details.

The crystal structure of wild-type human cardiac muscle I27 (residues 5253–5341, renumbered to 1–89 for simplicity) has been solved to 1.8Å by molecular replacement, Figure 1A. There are six I27 molecules in the asymmetric unit arranged around the local non-crystallographic 6-fold axis, with the N-termini of I27 closest to the local axis and the C-termini pointing outward. The entire amino acid sequence from residue 1–89 is visible in electron density (except L89 in chain E), as well as an additional 3–4 residues left from the TEV cleavage site after the N-terminal H6-tag (-AMA- in chain A; none in chain B; and -GAMA- in chains C–F, see Methods). The crystal structure reveals the well-known IG-like fold of the titin I-band IG domains and closely resembles the structure of the average NMR ensemble published earlier (PDB code 1TIT [23]); with 1.23Å root mean square distance (RMSD) between C_α_ carbons from all superposed residues (Figure S1). The largest distance (3.66Å for G53) is explained by the presence of a drastically different turn conformation between strands D and E, removing this turn (residues 52–55) reduces global RMSD to 1.05Å. As a comparison, the RMSD between non-crystallographic symmetry-related chains in the I27 crystal structure ranges from 0.16–0.35Å. Residues involved in the H-bond pairing across the A'G strand have RMSD between 0.6–1.4Å (res. 11–16) and 0.5–1.5Å (res. 78–87). After globally fitting to the wild-type backbone, the RMSD at the position of the two mutations encountered in the NMR structure (A42T, T78A) is 0.4 and 0.6Å, respectively. Crystallographic refinement statistics are given in Tables S3 and S4.

The present crystal structure of I27 represents an improved model for further studies because it does not contain potential structural perturbations introduced by unwanted mutations, and due to the high quality diffraction data, with overall coordinate error of 0.15Å. Overall, there are more and shorter H-bonds in the crystal structure comparing to the previously described NMR structure [23]. An additional H-bond between E3 and S26 and a stronger bond between K6 and E24 are found between strands A and B. While the hydrogen bonding between the A' and G strand largely overlaps, the interaction between Y9-N83 is absent but E12-K87 are within interaction distance, the latter supposedly adding resistance. Of the two residues mutated in the NMR structure, T78 (first residue of strand G) makes a side chain H-bond interaction with the carbonyl oxygen of L2 (preceding strand A) which is absent in the NMR structure and which could potentially add further mechanical resistance to I27. The implications of the differences observed between the interaction networks of both I27 structures for atomic force microscopy (AFM) and molecular dynamics studies are obvious, since site-directed mutagenesis of the residues involved in those interactions has been used as a tool to discern the mechanical properties of I27.

### Force Distribution

To elucidate distribution of mechanical stress in the titin I27 domain, we directly calculate forces 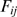 between each pair of atoms *i* and *j* during MD simulations of the high-resolution crystal structure of I27 described above. Forces are calculated individually for bonded and non-bonded (electrostatic and van der Waals) interactions below the cutoff distance using the interaction potentials defined by the OPLS [24] force field. By considering pairwise instead of atomic forces, forces do not average to zero over time. The propagation of the externally applied mechanical perturbation is measured as the change in pairwise forces upon applying external stress, 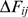, defined as the difference between the average force in the strained state and the relaxed state as obtained from equilibrium and force clamp (FCMD) [25] molecular dynamics simulations, respectively. In the strained state, as *in vivo*, force is applied to the termini of I27 in opposing direction. It remains controversial if IG domains in muscle titin ever fully unfold under physiological forces [26]–[28]. We here are interested in the force propagation within the fully folded protein, the physiologically relevant force-bearing structure. To this end, we apply a constant force of 300 pN, low enough to keep the protein structure intact. Importantly, no break in the AB strand, which is known as the first rupture event during I27 unfolding [29], was seen during 20 ns of simulation time. The heavy atom RMSD between average equilibrium and FCMD structures was ∼1.6Å for all simulations. For convergence, forces were averaged over ten equilibrium and eight FCMD trajectories, each 20 ns in length, respectively. To reduce noise further, mainly resulting from slow side chain fluctuations, data were normalized as described in Methods. Dimensionless normalized changes in force are denoted Δ*f*.

**Figure 2:**
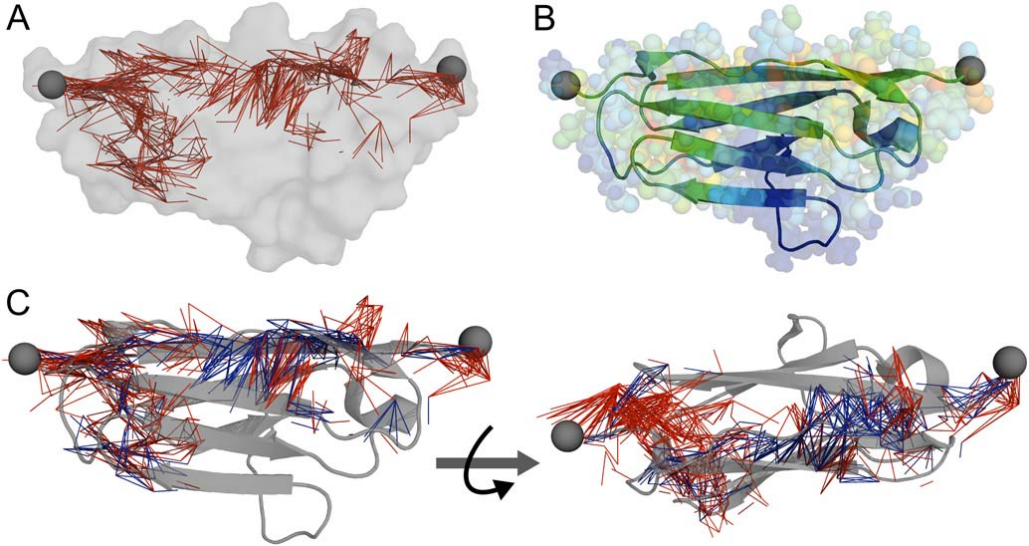
Force distribution in I27. (A) Graph representation of changes in interatomic forces, 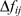, observed upon mechanical perturbation of I27. Edges connect non-bonded atom pairs with 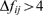. The protein surface is shown in gray. A 3D animation of this figure is available as Video S1. (B) Changes in atomic forces, Δ*f*, mapped onto the protein structure. Colors range from blue for elements outside the mechanical network with Δ*f* = 0 to red for force bearing elements with high Δ*f*. (C) Graph representation of 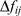 displayed as edges as in (A). I27 is shown as cartoon. Edges connecting main-chain atoms are colored blue, those connecting side chain atoms are colored red. Mechanical load at the C-terminus is mainly taken up by main-chain interactions around the A'G strand, whereas at the N-terminal side forces are primarily propagating into the protein core by side chain interactions.

Regarding the previously established importance of of inter-sheet hydrogen bonding for mechanical robustness [29], the highly resolved non-bonded interactions in the crystal structure are important for the atomic detail force propagation network we are analyzing here. Figure 1B shows the normalized force distribution along the protein sequence, 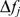, obtained from summing over all changes of pairwise forces of atom *j* upon stress application. The high signal-to-noise ratio indicates statistical significance of the data, with a remaining average error <35 of normalized force estimated from equilibrium data. Importantly, the force distribution pattern forms a spatially connected network of residues (Figure 2A and Video S1). The overwhelming majority of force signals are part of a network spanning the protein between the stretched termini, suggesting that the network indeed reflects propagation of the external stress into the structure. Remarkably, the mechanical network is sparse in the sense that large parts of the protein including strands C, D, E and F are not part of the network and thus apparently play no direct role in mechanical stability (Figure 2B).

The pattern is found to be highly anisotropic, with the terminal strands passing the tension along the strands adjacent to the force application sites and partially into the protein core through a connected network of mainly hydrophobic interactions spanning between I2, V4, N77, I23, L25, V30, W34, H56, F73 and T78 from the N-terminal side and V11, A19, F21, L60, M67, and L84 from the C-terminal side. We observe very different mechanisms of force distribution for the A'G and AB strands (Figure 2C). The A'G strand, known to be crucial for mechanical resistance [29], forms a mechanical clamp. Under load it shows a strong increase in interstrand H-bond (blue) and side chain forces (red). This is accompanied by a stiffening of the strand and neighboring residues as reflected by decreased root mean square fluctuations (Figure S2). In contrast, the AB strand, even though it has been shown to be the first point of rupture of the IG fold, does not show any major contribution to mechanical stability (Figure 2C), in agreement with experimental data [14]. Instead, force applied at the N-terminus is directly deflected into the protein core via mainly hydrophobic side chain interactions (red edges) between strands A and G, bypassing the AB interstrand hydrogen bonds, what again involves stiffening (Figure S2). This illustrates that rupture points are not necessarily involved in taking up large conformational load. Interestingly, signal propagation via side chain interactions involving stiffening of connected residues was previously observed experimentally in another globular protein [17]. A number of structural features, such as the A'G interstrand hydrogen bonds or the interactions involving T78, which was absent in the NMR structure due to the T78A mutation, are part of the force-bearing network. The determination of a refined crystal structure thus served as an important basis for our force distribution analysis.

### Comparison of the Mechanical with the Evolutionary Network in IG Domains

Sparse networks which span protein molecular structures in a spatially connected and anisotropic way have been previously observed for evolutionary couplings [18],[19]. An obvious assumption is that coevolved and therefore presumedly functionally important residues are involved in distributing and sustaining mechanical stress in IG domains. We tested this by comparing the force distribution pattern with evolutionary data from the IG domain family. Statistical coupling analysis (SCA) [18],[19] was performed to identify coevolved residues. As a measure of coevolution, the impact of a perturbation in the amino acid frequency at one site in a multiple sequence alignment (MSA) on the frequency at another site is used, here termed statistical coupling energy ΔΔ*E*. We constructed a MSA denoted 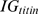 (Dataset S1) containing sequences from the 152 IG domains found in human muscle titin. We thereby restricted the sequences to those evolved for the specific function of bearing mechanical load.

**Figure 3:**
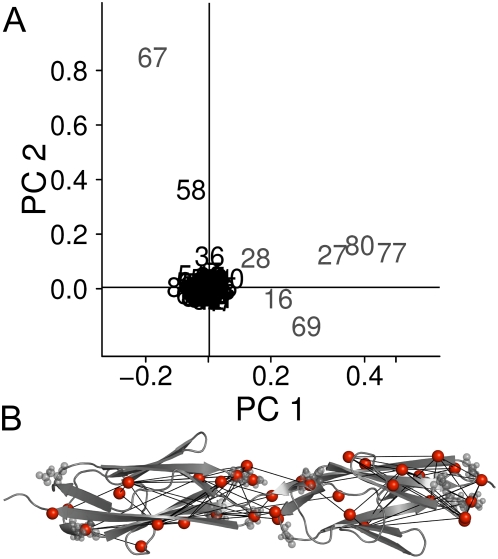
Coevolved interaction interface. (A) Principal component analysis on the perturbation matrix (see Methods) separates a set of residues along the first component (gray numbers). (B) Mapping these residues on the adjacent titin domains I67 and I68 (PDB-Code 2RIK [30]) reveals that they apparently belong to a conserved IG-IG interaction interface. Interaction interface residues are marked as gray spheres. Edges connect highly coevolved residues (red spheres) with ΔΔ*E*>0.7.

Mapping of the highly coupled residues on the I27 structure shows many of the couplings to span a spatially connected network between physically close residues residing in the protein core. In contrast, a subset of coevolved residues was found to couple distantly. It apparently belongs to a conserved IG-IG interaction interface, which becomes obvious when mapping coevolved residues onto two adjacent IG domains (PDB-entry 2RIK [30], Figure 3B). Indeed principal component analysis on the perturbation matrix (see Methods) clearly separates a subset from the bulk that coincides with the interaction interface, namely residues G16, E27, P28, M67, G69, N77, and S80 (Figure 3A). For direct comparison with the force distribution analysis, which was restricted to interactions within one domain, we exclude these interface residues from further analysis.

**Figure 4:**
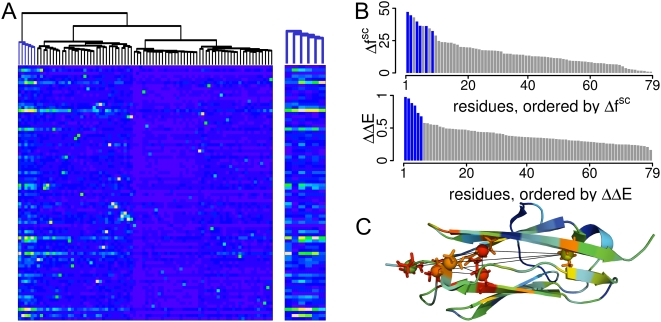
Overlap of the mechanical and coevolutionary network. (A) Heatmap of the clustered, symmetric coupling matrix containing ΔΔ*E* values for each pair of residues, interaction interface residues were excluded. The cluster containing residues important for mechanical stability is marked in blue (augmented plot). Heatmap colors range from blue for ΔΔ*E* = 0 to yellow for high ΔΔ*E* values. (B) Comparison of evolutionary and mechanical couplings. Inter-side chain forces 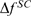 and evolutionary couplings ΔΔ*E* are shown as barplots and sorted in descending order; interface residues were excluded. The six residues forming a highly connected cluster via evolutionary couplings, colored blue, are found to be among the highest 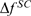 values. The average error in the (dimensionless) 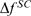 values is <5 as estimated from equilibrium data (Figure 1B). (C) Structural overlap of the evolutionary with the mechanical network. The six clustered residues shown in blue in (B) mapped as spheres/sticks onto the 1WAA structure. Sticks are colored with 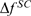 and spheres with ΔΔ*E*. SCA identifies the six residues as highly coevolved, edges show couplings between these residues with ΔΔ*E*>0.7. The secondary structure was colored with 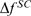 to give an overview of the overall force distribution.

We compared the evolutionary network with the force distribution pattern in I27; hereto we restricted our analysis to inter side chain forces, 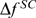 (see Methods), as evolution mainly optimizes side chains. A distinct group of residues with highest evolutionary couplings, residues I23, V4, F73, I2, V30, and L84, were found to mainly couple among each other and to clearly separate from the bulk, as indicated by hierarchical clustering analysis on the coevolution data (Figure 4A) Remarkably, these evolutionarily strongly connected residues show a very strong response to the applied mechanical perturbation, being among those showing highest changes in force distribution values (Figure 4B). This suggests that evolutionary and force distribution analysis show an overlapping set of residues which are crucial for mechanical robustness (Figure 4C). The overall comparison of evolutionary couplings with inter-residue forces of all IG residues indicates a connection of the evolutionary with the mechanical network as well. ΔΔ*E* and 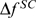 show a significant correlation, shown in Figure S3A, with a correlation coefficient of *R* = 0.52 (*t* = 5.56 and 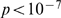 for 86 data points as calculated using student's t-test). This correlation is remarkable, regarding that the two data sets, from molecular simulations and sequence analysis, are completely independent. Furthermore, constraints acting on the evolution of proteins can be expected to be of manifold nature and thus to blur the correlation. One of these additional constraints is the optimization of the IG-IG interaction interface. Indeed, excluding the interfacial residues (see above) increases the correlation coefficient to *R* = 0.60 (with *t* = 6.62 and 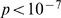 for 79 data points). For the same reason, the correlation is expected to weaken when including sequences into the alignment that are not necessarily designed to bear mechanical load. To test this, we constructed a second more diverse alignment denoted 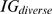 (Dataset S2), containing 282 sequences with high similarity to the I27 structure. Results from coupling analysis for 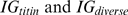 overlap (Figure S3B), suggesting that the observed conservation pattern is robust with regard to the MSA. We observe a lower correlation for 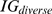 than for 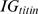 (0.37 vs. 0.52) corroborating our conclusion that the overlap found between evolutionary couplings and force distribution reflects an optimization for mechanical robustness of IG domains. Similarly, overlap of 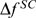 with overall residue conservation is low (*R* = 0.18), suggesting that it is the network rather than individual residues that are important for mechanical stability.

An alternative explanation for the observed correlation could be packing interactions of core residues that give rise to both, high evolutionary dependencies and high inter-residue forces. In particular, coevolution has been suggested to primarily reflect packing interactions or constraints from structural integrity or thermodynamic stability. We however find the correlation between ΔΔ*E* and packing density, measured as the number of close atomic contacts in I27 (within a 6Å cutoff), to be low, with *R* = 0.23. The correlation of ΔΔ*E* with 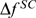 of *R* = 0.52 is significantly higher (*p*<0.05), and thus can barely, if at all, be explained by packing density. Similarly, changes in thermodynamic fold stability of I27 upon point mutation, as measured previously for 29 residues [31], do not correlate with ΔΔ*E* (*R* = 0.19). Instead, we find the fold stability to correlate significantly with the number of close contacts (*R* = 0.71). Consequently, while thermodynamic stability can be largely explained by core packing constraints, the evolutionary couplings can not be considered to reflect a simple spatial relationship or thermodynamic constraints. While mechanical and evolutionary couplings are mainly found between residues vicinal in the structure, vicinity in turn is not an indicator for strong coupling.

### Unfolding Simulations of I27 Mutants

By force distribution and coevolution analysis, we have identified the force-bearing scaffold of IG domains. The analysis predicts these residues to be crucial for mechanical function. We therefore expected a loss in mechanical stability upon their mutation, and directly tested this by forced unfolding of I27 *in silico* mutants in force-probe MD (FPMD) simulations [25].

**Figure 5:**
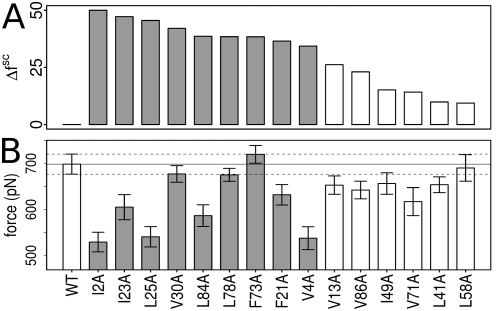
Forces needed for transition into the intermediate state measured for a selected set of *in silico* mutations. (A) 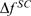 values for the mutated residues sorted in descending order. The nine residues with highest 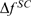, for which decreased rupture forces are expected, are colored gray. (B) Rupture forces observed for transition into the intermediate. The majority of the nine residues with highest 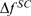 shows a significant decrease in rupture forces comparing to the wild-type (WT), while residues with significantly lower 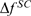 show less impact onto rupture forces upon mutation. The overall decrease comparing to the WT and to the six negative controls is statistically significant (*p*<0.01 and *p*<0.03, respectively, student's t-test on cumulative rupture force data. For individual p-values, see Table S1).

We here considered the force distribution of the native state as the physiologically most relevant state of IG. We thus monitored force changes for the rupture of the AB strand, the primary unfolding event [29], and of the A'G strand, whose rupture is visible as the highest force peak in AFM experiments, upon *in silico* point mutations. We selected the nine residues with highest 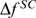 values, which include the cluster of highly coevolved residues, and performed at least 10 independent FPMD simulations for each candidate after mutation to alanine. For most of the mutants we observed statistically significantly lower forces (*p*<0.01 in student's t-test, Table S1) required to unfold into the intermediate state, Figure 5A and 5B. Interestingly, three of the residues with high 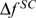 instead show little change in mechanical resistance upon mutation. In addition, none of the mutations gave rupture forces smaller than 500 pN. This may reflect the robustness of the mechanical network to local perturbations, allowing it to re-balance load. We performed six control simulations of residues that featured low 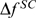 values despite their location in the protein core. As expected, none or only minor decreases in rupture forces are found, with an overall destabilization significantly lower than for the nine selected high 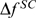 candidates (*p*<0.03, Figure 5B).

**Figure 6:**
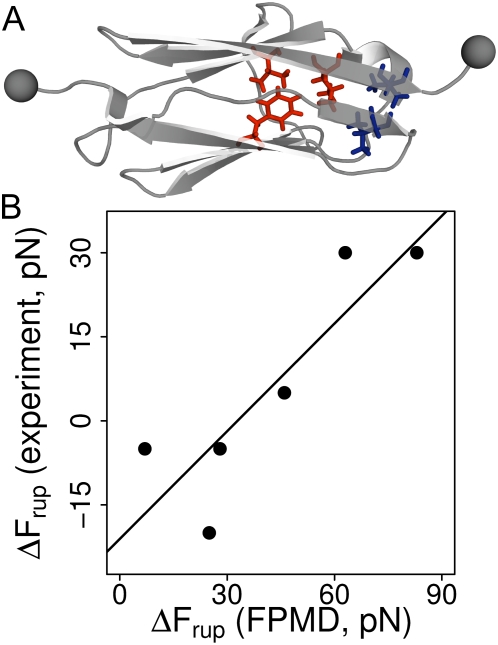
Comparison of *in silico* rupture forces for the A'G strand with experimental data. (A) Our simulations predict decreased stability of the A'G strand for 5 residues near the A'G strand, rendering them interesting targets for further experimental studies. The figure shows these residues mapped as sticks on the I27 structure. F21, L84 and V71 are colored blue, the experimentally validated V13 and V86 are colored red. (B) Changes in rupture force 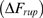 from experiment and simulation plotted against each other. The line shows a fit of the data to a linear model. Experimental data for a pulling speed of 10 nm s^−1^ were extracted from Figure 3, Best et al. 2003 [14].

Several residues located in the A'G strand show a high degree of force distribution into the protein core, including F21 and L84, as indicated by their high 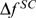 signal, in contrast to the common notion of the primary importance of interstrand hydrogen bonding. Indeed, removing the hydrophobic contacts of F21 and L84 results in significantly decreased rupture force of the A'G strand (Figure 6A). This renders F21 and L84 interesting targets for further experimental studies and stresses our observation that specific core interactions are important force-bearing interactions.

Force spectroscopy experiments mainly probe the later step of unfolding of the intermediate via the C-terminal A'G strand rupture, since it involves the highest rupture forces [29]. For validating our FPMD simulations with experimental data, we monitored rupture forces of this unfolding step. We included additional *in silico* mutations for comprehensive comparison with a series of AFM experiments done by Clarke and co-workers [14]. Our results are in good agreement with their data, with a significantly decreased rupture force only found for the V13A and V86A mutants (Figure 6B), but not for the others.

## Discussion

We present here the first force distribution network in a protein at atomic detail, thereby revealing the force-bearing scaffold rendering I27 mechanically robust. The network is in good agreement with previous experimental results [12],[14] and provides a basis for the choice of mutants to rationally alter the mechanical response, even at sites distant from force application. Our force distribution analysis can be directly tested by force probe experiments of such mutants, and can in future allow the design of proteins having specific mechanical stability and response. It can also help in explaining and engineering stability in biological as well as synthetic materials, such as silk or polymers. Previous attempts to detect force distribution in proteins have been restricted to elastic network models [32]–[34], which assume linear elasticity of the interactions. Importantly, we find the non-linear nature of non-bonded interactions to give rise to stiffening of structural motifs. Examining the extent of non-linearity in the mechanical response of IG and other proteins by determining the force pattern as a function of external stress will be the subject of future studies, and will allow the development of coarse-grain models without the need for assuming harmonic interaction potentials. Furthermore, while in elastic network models sequence specificity is either neglected [32] or taken into account implicitly by modifying inter-residue force constants [33],[34], our pairwise atomic analysis is directly sensitive towards residue types.

An interesting question is how long an externally applied perturbation needs to propagate through a protein, which is not directly accessible by force spectroscopy. We find a sub-nanosecond time scale to be sufficient for convergence of forces in I27 (Figure S4), a rigid protein not undergoing major conformational changes upon stretching. Remarkably, this time scale is comparable to that measured experimentally for heat transport in helices [35] and to previous theoretical studies [36]. However, a more detailed force distribution analysis that is both time and spatially resolved is required to determine the timescale for force propagation and mechanical equilibration, which is the aim of future studies.

The mechanical response of I27 appears to be remarkably robust with regard to point mutations within the force distribution network, which lower the rupture force never by more than ∼30%. In particular, a small subset of highly force bearing residues (V30, F73, L78) do not cause any loss of overall stability upon mutation. This suggests a certain redundancy or plasticity of the mechanical network. Biological networks ranging from interaction networks to gene regulatory networks are increasedly well characterized and understood [37],[38]. The force network described in this study represents a new type of biological network, which asks for graph theoretical analysis to further clarify its function, including splits, redundancy, hubs, and other properties.

We hypothesize that strain propagation as revealed here acts in an allosteric protein as a mechanism to transduce an external signal through the protein core to distant sites. The force distribution analysis was here applied to the propagation of mechanical force as a perturbation acting on a protein, but can easily be extended to other types of perturbation, in particular to allosteric signals. Since forces can monitor allosteric signal propagation more sensitively than coordinates, our method is particularly suited for allosteric proteins not undergoing an obvious conformational change, i.e., rigid proteins [39], and dynamic allostery [40] for which changes in fluctuations in pairwise forces can be expected.

Networks of coevolved residues and their relevance for protein stability and function have been exhaustively analyzed for many proteins [18], [19], [21], [41]–[43]. It has been suggested that the molecular mechanism by which coevolved residues couple is of dynamic or energetic nature [20]. Couplings in binding free energies [44] have not yet been unambiguously correlated with evolutionary couplings. Recently, attempts have been made to compare couplings in dynamic fluctuations with evolutionary couplings [45]. In contrast to the notion of functional protein fluctuations propagating force, we here propose the stiffness, i.e., the static nature, of the force-bearing scaffold to be functionally crucial (Figure S2). The relevance of this first molecular interpretation of evolutionary design for other proteins, with mechanical, allosteric, or other functions, remains to be investigated.

## Methods

### Force Distribution

We use a modified version of Gromacs 3.3.1 to write out forces 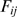 between each atom pair *i* and *j*. Forces include contributions of individual bonded (bond, angle, dihedral) and non-bonded (electrostatic and van der Waals) terms below the cutoff distance, which are stored separately for further analysis. The force between each atom pair is represented as the norm of the force vector and thus is a scalar. Attractive and repulsive forces are assigned opposite signs, forces are averaged over simulation time and converge to an equilibrium value. As we consider the direct force between each atom pair, this equilibrium value can be different from zero, even for the theoretical case of a system without any motion. We hereby obtain the advantage to be able to observe signal propagation even through stiff materials, where forces propagate without causing major atomic displacement. Atomic forces, i.e., the sum over the force vectors acting on an atom, instead average out to zero over time and are not of interest here. A real world example for such force propagation is Newton's cradle. Due to the nature of the non-bonded potentials pairwise forces are most significant for atom pairs in relative close proximity, resulting in a force-propagation network comprised of a series of short to medium ranged connections.

An approximation is used to represent angle and dihedral forces (Figure S5). Multi body forces such as hydrophobic effects and PME forces are not included and thus cannot be accounted for in the analysis. Average forces were written out every 10 ps. To obtain converged averages, forces for each atom pair were averaged for each trajectory and afterwards over all pulling and equilibrium trajectories, respectively. The normalized change in force is defined as the difference between forces in the strained state, 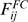, and equilibrium forces, 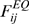, for each atom pair *i*,*j*. Normalization by the standard error of the mean ε accounts for differences caused by insufficient sampling, i.e., slow side chain or backbone fluctuations that cannot equilibrate in simulation time.
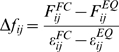
(1)The overall distribution, however, remains largely unchanged upon normalization, Figure 1C. The mechanical coupling of an atom *j* with respect to all other atoms is then defined as the absolute sum 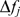.
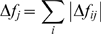
(2)


### Side Chain Forces

In analogy, for all pairs of side chains *u* and *v*, we sum up forces 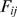, where atom *i* and atom *j* must not be part of the same residue. Forces 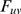 are averaged for each trajectory and afterwards over equilibrium and pulling trajectories. The normalized change in inter side chain forces is defined as the difference between force clamp 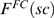 and equilibrium 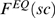 forces normalized by the standard errors 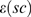 observed between equilibrium and force probe trajectories, respectively.
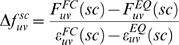
(3)We then define the mechanical coupling for residue *v* as the absolute sum 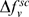.
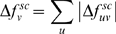
(4)


### Molecular Dynamics (MD) Simulations

were carried out using Gromacs 3.3.1 [46]. The OPLS all atom force field [24] for the protein and the TIP4P [47] water model were employed. The crystal structure of I27 (PDB-entry 1WAA) was used as starting structure for all simulations. Simulation times were 20 ns for equilibrium and FCMD simulations. A constant force of 300 pN was applied to both terminal residues in opposing direction. The applied force was low enough to keep the protein structure intact and no partial rupture events were seen during the simulation time. Simulations were run in the NpT ensemble, temperature was kept constant at 300 K by coupling to the Berendsen thermostat [48]. The pressure was kept constant at p = 1 bar using anisotropic coupling to a Berendsen barostat [48] with 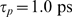 and a compressibility of 4.5 10^−5^ bar^−1^ in the x, y, and z directions. In FPMD simulations of the I27 mutants all bonds were constrained using the LINCS [49] algorithm; an integration timestep of 2fs was used. No constraints and an integration timestep of 1fs was used for equilibrium and FCMD simulations. Lennard-Jones interactions were calculated using a cutoff of 10Å. At a distance smaller than 10Å, electrostatic interactions were calculated explicitly, whereas long-range electrostatic interactions were calculated by Particle-Mesh Ewald summation [50]. System coordinates were saved every 10 ps.

The X-ray structure of I27 (PDB-entry 1WAA) was used as starting structure for all simulations. Protonation states of histidines were determined by optimizing the hydrogen bond network using Whatif [51]. All mutations were done using Whatif, starting from the equilibrated structure. Structures were solvated in a triclinic box with dimensions 55×55×100Å, containing ∼40,000 atoms. Sodium and chloride ions corresponding to a physiological ion strength of 100 mM were added. An energy minimization of 1000 steps using the steepest descent algorithm was followed by a 400 ps MD simulation with harmonical restraints on the protein heavy atoms with a force constant of k = 1000 kJ mol^−1^ nm^2^ to equilibrate water and ions. For mutants, this simulation was followed by a 1 ns MD simulation with the same harmonical constraints on backbone atoms only. A subsequent free MD simulation of 5 ns length was performed to equilibrate the whole system, during which the protein backbone root mean-square deviation (RMSD) was monitored. All mutants remained stable during free MD, with a heavy atom RMSD to the starting structure <2Å for all structures (Table S2). For each run, new velocities were chosen form a random distribution using Gromacs, followed by a 400 ps MD simulation with restraints on protein heavy atoms as described above. During unfolding simulations partial rupture at the N-terminus that leads to the intermediate state was measured by means of the distance between the 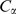 of S26 and the backbone *N* of E3. Rupture of the A'G strand was measured by monitoring the length of the V13-K85 hydrogen bond.

Mutants were partially unfolded during a 12 ns FPMD simulation with a harmonic spring potential applied on both terminal residues, with a spring constant of k = 500 kJ mol^−1^ nm^−1^. The springs were moved with a constant velocity of 0.02Å ps^−1^ each.

### Statistical Coupling Analysis (SCA)

Coupling analysis was carried out as described by Ranganathan and co-workers [18],[19]. SCA assigns a “statistical coupling energy” ΔΔ*E* to each position in a multiple sequence alignment. ΔΔ*E* is determined upon perturbation by removal of sequences of the alignment that changes the amino acid distribution at a specific position. If the observed amino acid distribution at position *b* changes with respect to perturbation at position *a*, these positions are “coupled”. Intuitively, if evolution changed residue *a* it is also likely to change residue *b* to maintain the protein's functionality, i.e., *a* and *b* are statistically dependent throughout evolution. We perform a set of small perturbations to the MSA by removing one row at a time. Each perturbation results in a small fluctuation of ΔΔ*E* values for each position. The matrix containing perturbations versus ΔΔ*E* fluctuations is referred to as perturbation matrix, and PCA was done on this matrix. We then define the coupling score between two positions as the cross product of the two ΔΔ*E* trajectories, which is the norm of the two position vectors in perturbation space.

We perform SCA on two sets of sequences. The first, 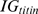, contains the sequences of all 152 IG domains in human muscle titin (UniProtKB/Swiss-Prot Q8WZ42). The second more general alignment 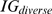 was chosen from the IG I-set (Pfam id: PF07679) family by similarity to I27 and contains 282 sequences. Hereto a Blast [52] search against all sequences from the I-set family was done and sequences with e-score <1.00 were selected. All sequences with similarity >90% to any other sequence were removed. SCA requires the set of sequences to be sufficiently diverse. Further the set of sequences should be well balanced, in the sense that the average similarity to all other sequences should be roughly the same for each sequence. We checked the diversity of sequences within the 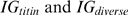 alignments and found it to be comparable to results published earlier. I.e., the average sequence similarity within IG domains of human titin of 0.46 compares well with the average similarity of 0.48 for the alignment of PDZ-domain sequences used by Ranganathan and co-workers [18]. Figure S3C shows that the variance in average sequence similarity is low, indicating that both alignments are well balanced. To assess the alignment quality, we calculated the sequence entropy [53], a measure for conservation at each position, and again find it to be very comparable with data published earlier (*PDZ* = 1.95, 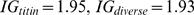). Sequences were aligned using Dialign [54] that is reported to perform well on local sequence alignments [55]. In the final alignments, no position aligned to the I27 sequence contained more than 50% gaps.

### Correlation with Side Chain Forces

For the calculation of correlations with inter-side chain forces the terminal residues which were directly subjected to force and the ultra-conserved W34 were excluded. Hierarchical cluster analysis was done using Ward's algorithm [56] as implemented in R (R Development Core Team). At each step in the analysis, the method considers the union of every possible cluster pair, and the two clusters with minimal square sum of error are combined. The city-block metric was used as distance measure. For the comparison of 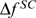 with residue conservation, conservation was calculated using Shannon entropy.

### Structure Determination and Refinement

The I27 structure was determined by molecular replacement with AMORE [57] using as search model a representative structure from a previously determined NMR ensemble (PDB-entry 1TIT [23]). Of the six copies of I27, four copies were orientated and placed successively with AMORE. Correctness of this incomplete model was assessed by the increase in correlation coefficient (from 24.8% to 40.4%) and the concomitant decrease in R-factor (from 50.2% to 45.2%) attained between the first and the fourth I27 molecule. The last two copies of I27 could not be automatically located with AMORE using the same protocol. Instead, this first model containing four I27 molecules was refined (rigid body) and then the missing two copies of I27 were placed manually. Since the I27 NMR structure contained two mutations not present in the wild type titin sequence (A42T, T78A), the sequence in our I27 structure was modified accordingly. Refinement and modeling was performed iteratively using REFMAC5 [58] and Turbo-Frodo (http://www.afmb.univ-mrs.fr/-TURBO-). The model has been refined to a final R-factor of 0.211 and R-free of 0.268 (Table S3). Upon convergence, the maximum-likelihood coordinate error estimation is 0.15Å.

### Protein Expression, Purification and Crystallization

Residues 5253–5341 of human cardiac titin I27 (renumbered 1–89 for simplicity) were amplified from a cDNA clone (accession code X90568 of the EMBL data library) by polymerase chain reaction (PCR) and subcloned into the pETM11 expression vector for expression of I27 in *E. coli* fused to a TEV (tobacco edged virus protease) cleavable N-terminal His_6_-tag. An overnight preculture of BL21(DE3) cells transformed with the I27 expression plasmid was used as inoculum to 3 liters of Luria-Bertani medium plus 50 µM kanamycin, and I27 expression was induced at OD_600_ of 0.6 by the addition of 1 mM IPTG. Cells were harvested 3 h post induction, lysed by sonication in 20 mM Tris-HCl, 300 mM NaCl, 20 mM imidazole, pH 8.0, and clarified by centrifugation at 20,000×g and filtration through a 0.22 µm membrane. The supernatant, containing soluble protein, was poured on a Ni^2+^-NTA agarose column and I27 was eluted with a linear imidazole gradient. Elution fractions containing I27 were pooled together and incubated with TEV protease for 3 h to remove the affinity tag. The cleaved tag and TEV were removed by passing the digestion over a second Ni^2+^-NTA column (Qiagen). The flow-through, containing cleaved I27, was dialysed overnight against 20 mM Tris-HCl, 2 mM DTT, pH 8.0, loaded onto a MonoQ (GE Healthcare) ionic exchange column and eluted with a 0–1 M NaCl linear gradient. Final polishing of I27 was brought about by gel filtration chromatography on a Supedex 75 (GE Healthcare) column. Purified I27 was concentrated to 10 mg/ml in 20 mM Tris-HCl, 50 mM NaCl, pH 7.5.

### Diffraction Data Collection and Processing

Crystals of I27 were grown by hanging drop vapor diffusion in 20% PEG MME 550, 75 mM MES, 7.5 mM ZnSO4 at pH 6.5. The crystals were mounted in Hampton Research nylon loops, and cryoprotected in a cryosolution made of the mother liquor and 5% (v/v) glycerol. Crystals belonged to P2_1_2_1_2_1_ space group and X-ray data up to a maximum resolution of 1.8Å were collected on beam-line BW7B (EMBL, Hamburg, DESY) at a wavelength of 0.841Å at 100 K. One segment of 90° was sliced in 0.5° rotation steps to give complete and redundant data. Diffraction data were processed with MOSFLM [59] and scaled in SCALA [60] (Table S4). Cell content analysis and self-rotation function calculations indicated that the asymmetric unit contained 6 copies of I27.

## Supporting Information

Dataset S1 Multiple sequence alignment - IG_titin_ alignment
(0.16 MB DOC)

Dataset S2 Multiple sequence alignment - IG_diverse_ alignment
(0.34 MB DOC)

Figure S1 Root mean square distance between C_α_ carbons of the NMR ensemble (1TIT) and the newly determined crystal structure of I27.
(0.07 MB TIF)

Figure S2 Stiffening of force bearing parts under load in I27 indicated by decreased root mean square fluctuations (RMSF). The observed decrease in RMSF corresponds well with the force distribution pattern. (A) Differences in RMSF between equilibrium and force clamp simulations plotted along the protein sequence. (B) RMSF differences color coded on the I27 structure. Colors range from blue for no change to red for high change. Data are averages over 10 equilibrium and 8 FCMD simulations, with 20 ns simulation time each.
(2.75 MB TIF)

Figure S3 Correlation of the evolutionary and mechanical network and quality assessment for the multiple alignments. (A) Correlation of evolutionary ΔΔE values with inter side-chain forces Δf^sc^. Plotted are ΔΔE versus Δf^sc^ including (left) and excluding (right) interaction interface residues, yielding correlation coefficients of R = 0.52 and R = 0.60. The lines show the fit of a linear model to the data. (B) Correlation between pair-wise statistical coupling ΔΔE values for IG_titin_ and IG_diverse_, yielding a correlation coefficient of R = 0.65. The line shows the fit of a linear model to the data. (C) The average sequence similarity for IG_titin_ and IG_diverse_ is comparable with the PDZ alignment published by Ranganathan and co-workers.
(0.78 MB TIF)

Figure S4 Speed of signal propagation in I27, shown are forces averaged over all atom pairs in I27 during equilibrium (left) and FCMD simulations (right). Each data point corresponds to the average force during 100 ps. The global means are plotted as black lines.
(0.27 MB TIF)

Figure S5 Approximations used for angle and dihedral terms. The OPLS force field uses angle and dihedral terms calculated as multibody forces between atoms i,j,k for angles and atoms i,j,k,l for dihedrals. For angles, Gromacs internally calculates the force vectors I,J,K acting on these atoms. As we cannot directly map such multibody forces to pairwise interactions, we represent angle bending as |K-I|, the force component acting in i,k direction. Similarly, for dihedral terms the force vectors I,J,K,L acting on the atoms i, j, k, l are calculated. To represent bending of dihedral angles we use |L-I|, the force component acting in direction i,l. This will not provide physically correct forces, but is sufficient to detect rearrangements under mechanical load.
(0.25 MB TIF)

Table S1 T-tests for unfolding forces of *in silico* mutants.
(0.05 MB DOC)

Table S2 Backbone and heavy atom root mean square deviation (RMSD) in Angstroem for I27 *in silico* mutants.
(0.04 MB DOC)

Table S3 X-ray refinement statistics.
(0.05 MB DOC)

Table S4 Crystallographic statistics, data processing and scaling.
(0.04 MB DOC)

Video S1 A movie showing the force distribution network spanning I27.
(5.13 MB MOV)
