# Supplementary material for: Mechanical Network in Titin Immunoglobulin from Force Distribution Analysis
Source: PLoS Comput Biol. 2009 Mar 13;5(3):e1000306. doi: 10.1371/journal.pcbi.1000306 (PMC2643529; doi:10.1371/journal.pcbi.1000306)
Supplement: Dataset S1 — Multiple sequence alignment - IGtitin alignment (0.16 MB DOC) [file pcbi.1000306.s001.doc]

>1WAA|DOMAIN_5253-5341

------------------------LIEVEKPLY--GVE----VFVGE-TA

HFEIELSE-PDVHGQWKLKGQP----------LA--A----S-------P

D-C-E----------------------------II-E-DG-KK--HILIL

HN--CQLGMTGEVSFQAANTKSAANLKVKEL---

>Q8WZ42|DOMAIN_6-96

-------------------------PTFTQPLQ--SVV----VLEGS-TA

TFEAHISGFPVPEVSWFRDGQV----------IS--T----STl-----P

G-V-Q----------------------------IS-F-SD-GR--AKLTI

PA--VTKANSGRYSLKATNGSGQATSTAELL---

>Q8WZ42|DOMAIN_104-192

-------------------------PNFVQRLQ--SMT----VRQGS-QV

RLQVRVTGIPTPVVKFYRDGAE----------IQ--S----S-------L

D-F-Q----------------------------IS-Q-EG-DL--YSLLI

AE--AYPEDSGTYSVNATNSVGRATSTAELL---

>Q8WZ42|DOMAIN_943-1031

-------------------------PTLVSGLK--NVT----VIEGE-SV

TLECHISGYPSPTVTWYREDYQ----------IE--S----S-------I

D-F-Q----------------------------IT-F-QS-GI--ARLMI

RE--AFAEDSGRFTCSAVNEAGTVSTSCYLA---

>Q8WZ42|DOMAIN_1082-1172

-------------------------PYFITKPV--VQK----LVEGG-SV

VFGCQVGGNPKPHVYWKKSGVP----------LT--T----G-------Y

R-Y-K----------------------------VS-Y-NK-QTGECKLVI

SM--TFADDAGEYTIVVRNKHGETSASASLL---

>Q8WZ42|DOMAIN_1291-1382

s-------------------------GFDLRIK--NYR----ILEGM-GV

TFHCKMSGYPLPKIAWYKDGKR----------IK--H----G-------E

R-Y-Q----------------------------MD-F-LQ-D-GRASLRI

PV--VLPEDEGIYTAFASNIKGNAICSGKLYVE-

>Q8WZ42|DOMAIN_1457-1546

-------------------------PVFVLKPV--SFK----CLEGQ-TA

RFDLKVVGRPMPETFWFHDGQQ----------IV--N----D-------Y

T-H-K----------------------------VV-I--K-EDGTQSLII

VP--ATPSDSGEWTVVAQNRAGRSSISVILT---

>Q8WZ42|DOMAIN_1556-1646

-------------------------PMFVEKLK--NVN----IKEGS-QL

EMKVRATGNPNPDIVWLKNSDI----------IV--PH--K---------

Y-P-K----------------------------IR-I-EG-TKGEAALKI

DS--TVSQDSAWYTATAINKAGRDTTRCKVN---

>Q8WZ42|DOMAIN_1703-1793

-------------------------PFFKKKLTslRLK----RFGPA-HF

ECRLTPIGDPTMVVEWLHDGKP----------LE--A----A-------N

R-L-R----------------------------MI-N-EF-GY--CSLDY

GV--AYSRDSGIITCRATNKYGTDHTSATLI---

>Q8WZ42|DOMAIN_1841-1928

-------------------------PDIVLYPE--PVR----VLEGE-TA

RFRCRVTGYPQPKVNWYLNGQL----------IR--K----S-------K

R-F-R----------------------------VR-Y-DG--I--HYLDI

VD--CKSYDTGEVKVTAENPEGVIEHKVKLE---

>Q8WZ42|DOMAIN_2078-2167

-------------------------PKIFERIQ--SQT----VGQGS-DA

HFRVRVVGKPDPECEWYKNGVK----------IE--R----S-------D

R-I-Y----------------------------WY-WpED-NV--CELVI

RD--VTAEDSASIMVKAINIAGETSSHAFLL---

>Q8WZ42|DOMAIN_2171-2262

k----------------------QLITFTQELQ--DVV----AKEKDtMA

TFECETSE-PFVKVKWYKDGME----------VH--E----G-------D

K-Y-R----------------------------MH-S-DR-KV--HFLSI

LT--IDTSDAEDYSCVLVEDENVKTTAKLIV---

>Q8WZ42|DOMAIN_2264-2354

g----------------------AVVEFVKELQ--DIE----VPESY-SG

ELECIVSP-ENIEGKWYHNDVE----------LK--S----N-------G

K-Y-T----------------------------IT-S-RR-GR--QNLTV

KD--VTKEDQGEYSFVIDGKKTTCKLKMKPR---

>Q8WZ42|DOMAIN_2353-2443

----------------------PRPIAILQGLS--DQK----VCEGD-IV

QLEVKVSL-ESVEGVWMKDGQE----------VQ--P----S-------D

R-V-H----------------------------IV-I-DK-QS--HMLLI

ED--MTKEDAGNYSFTIPALGLSTSGRVSVY---

>Q8WZ42|DOMAIN_2430-2529

PA----------LGLSTSgrvsvySVDVITPLK--DVN----VIEGT-KA

VLECKVSVPDVTSVKWYLNDEQ----------IK--P----D-------D

R-V-Q----------------------------AI-V-KG-TK--QRLVI

NR--THASDEGPYKLIVGRVETNCNLS-------

>Q8WZ42|DOMAIN_2620-2703

-------------------------GAISKPLT--DQT----VAESQ-EA

VFECEVAN-PDSKGEWLRDGKH----------LP--L----T-------N

N-I-R----------------------------SE-S-DG-HK--RRLII

AA--TKLDDIGEYTYKVATSKTSAKLK-------

>Q8WZ42|DOMAIN_2880-2965

-----------------------ETLHITKTMK--NIE----VPETK-TA

SFECEVSH-FNVPSMWLKNGVE----------IE--M----S-------E

K-F-K----------------------------IV-V-QG-KL--HQLII

MN--TSTEDSAEYTFVCGNDQVSATLT-------

>Q8WZ42|DOMAIN_2968-3050

------------------------PIMITSMLK--DIN----AEEKD-TI

TFEVTVNY-EGISYKWLKNGVE----------IK--S----T-------D

K-C-Q----------------------------MR-T-KK-LT--HSLNI

RN--VHFGDAADYTFVAGKATSTAT---------

>Q8WZ42|DOMAIN_3058-3141

-------------------------IEFRKHIK--DIK----VLEKK-RA

MFECEVSE-PDITVQWMKDDQE----------LQ--I----T-------D

R-I-K----------------------------IQ-K-EK-YV--HRLLI

PS--TRMSDAGKYTVVAGGNVSTAKLF-------

>Q8WZ42|DOMAIN_3239-3327

-------------------------PQVLQELQ--PVT----VQSGK-PA

RFCAVISGRPQPKISWYKEEQL----------LS--T----G-------F

K-C-K----------------------------FL-H-DG-QE--YTLLL

IE--AFPEDAAVYTCEAKNDYGVATTSASLS---

>Q8WZ42|DOMAIN_3344-3432

------------------------PPAIITPLQ--DTV----TSEGQ-PA

RFQCRVSG-TDLKVSWYSKDKK----------IK--P----S-------R

F-F-R----------------------------MT-Q-FE-DT--YQLEI

AE--AYPEDEGTYTFVASNAVGQVSSTANLS---

>Q8WZ42|DOMAIN_3503-3586

-------------------------PIFIKEVS--NAD----ISMGD-VA

TLSVTVIGIPKPKIQWFFNGVL----------LT--P----S-------A

D-Y-K----------------------------FV-F-DG-DD--HSLII

LF--TKLEDEGEYTCMASNDYGKTIC--------

>Q8WZ42|DOMAIN_3621-3712

-------------------------PHFLKELK--PIR----CAQGL-PA

IFEYTVVGEPAPTVTWFKENKQ----------LC--T----S-------V

YyT-I----------------------------IH-N-PN-GS--GTFIV

ND--PQREDSGLYICKAENMLGESTCAAELLVL-

>Q8WZ42|DOMAIN_4289-4376

-------------------------PMIHTPLV--DTV----SEEGD---

IVHLTTSITNAKEVNWYFENKL----------VP--S----D-------E

K-F-K----------------------------CL-Q-DQ--N--TYTLV

IDkvNTEDHQGEYVCEALNDSGKTATSAKLT---

>Q8WZ42|DOMAIN_4383-4471

-------------------------PVIKRKIE--PLE----VALGH-LA

KFTCEIQSAPNVRFQWFKAGRE----------IY--E----S-------D

K-C-S----------------------------IR-S-SK-YI--SSLEI

LR--TQVVDCGEYTCKASNEYGSVSCTATLT---

>Q8WZ42|DOMAIN_4478-4566

-------------------------PTFLSRPK--SLT----TFVGK-AA

KFICTVTGTPVIETIWQKDGAA----------LS--P----S-------P

N-W-K----------------------------IS-D-AE-NK--HILEL

SN--LTIQDRGVYSCKASNKFGADICQAELI---

>Q8WZ42|DOMAIN_4571-4659

-------------------------PHFIKELE--PVQ----SAINK-KV

HLECQVDEDRKVTVTWSKDGQK----------LP--P----G-------K

D-Y-K----------------------------IC-F-ED-KI--ATLEI

PL--AKLKDSGTYVCTASNEAGSSSCSATVT---

>Q8WZ42|DOMAIN_4664-4753

------------------------PSFVKKVDP--SYL----MLPGE-SA

RLHCKLKGSPVIQVTWFKNNKE----------LS--E----S-------N

T-V-R----------------------------MY-F-VN-SE--AILDI

TD--VKVEDSGSYSCEAVNDVGSDSCSTEIV---

>Q8WZ42|DOMAIN_4758-4846

-------------------------PSFIKTLE--PAD----IVRGT-NA

LLQCEVSGTGPFEISWFKDKKQ----------IR--S----S-------K

K-Y-R----------------------------LF-S-QK-SL--VCLEI

FS--FNSADVGEYECVVANEVGKCGCMATHL---

>Q8WZ42|DOMAIN_4851-4936

-------------------------PTFVKKVD--DLI----ALGGQ-TV

TLQAAVRGSEPISVTWMKGQEV-----------I-RE----D-------G

K-I-K----------------------------MS-F-SN-GV--AVLII

PD--VQISFGGKYTCLAENEAGSQTSVG------

>Q8WZ42|DOMAIN_4943-5032

------------------------PAKIIERAE--LIQ----VTAGD-PA

TLEYTVAGTPELKPKWYKDGRP----------LV--A----S-------K

K-Y-R----------------------------IS-F-KN-NV--AQLKF

YS--AELHDSGQYTFEISNEVGSSSCETTFT---

>Q8WZ42|DOMAIN_5040-5128

-------------------------PFFTKPLR--NVD----SVVNG-TC

RLDCKIAGSLPMRVSWFKDGKE----------IA--A----S-------D

R-Y-R----------------------------IA-F-VE-GT--ASLEI

IR--VDMNDAGNFTCRATNSVGSKDSSGALI---

>Q8WZ42|DOMAIN_5133-5221

-------------------------PSFVTKPG--SKD----VLPGS-AV

CLKSTFQGSTPLTIRWFKGNKE----------LV--S----G-------G

S-C-Y----------------------------IT-K-EA-LE--SSLEL

YL--VKTSDSGTYTCKVSNVAGGVECSANLF---

>Q8WZ42|DOMAIN_5225-5314

P-----------------------ATFVEKLEP--SQL----LKKGD-AT

QLACKVTGTPPIKITWFANDRE----------IK--E----S-------S

K-H-R----------------------------MS-F-VE-ST--AVLRL

TD--VGIEDSGEYMCEAQNEAGSDHCSSIV----

>Q8WZ42|DOMAIN_5320-5408

-------------------------PYFTKEFK--PIE----VLKEY-DV

MLLAEVAGTPPFEITWFKDNTI----------LR--S----G-------R

K-Y-K----------------------------TF-I-QD-HL--VSLQI

LK--FVAADAGEYQCRVTNEVGSSICSARVT---

>Q8WZ42|DOMAIN_5413-5501

-------------------------PSFIKKIE--STS----SLRGG-TA

AFQATLKGSLPITVTWLKDSDE-----------I-TE----D-------D

N-I-R----------------------------MT-F-EN-NV--ASLYL

SG--IEVKHDGKYVCQAKNDAGIQRCSALLS---

>Q8WZ42|DOMAIN_5505-5594

------------------------PATITEEAV--SID----VTQGD-PA

TLQVKFSGTKEITAKWFKDGQE----------LT--L----G-------S

K-Y-K----------------------------IS-V-TD-TV--SILKI

IS--TEKKDSGEYTFEVQNDVGRSSCKARIN---

>Q8WZ42|DOMAIN_5602-5690

-------------------------PSFTKKLK--KMD----SIKGS-FI

DLECIVAGSHPISIQWFKDDQE----------IS--A----S-------E

K-Y-K----------------------------FS-F-HD-NT--AFLEI

SQ--LEGTDSGTYTCSATNKAGHNQCSGHLT---

>Q8WZ42|DOMAIN_5695-5783

-------------------------PYFVEKPQ--SQD----VNPNT-RV

QLKALVGGTAPMTIKWFKDNKE----------LH--S----G-------A

A-R-S----------------------------VW-K-DD-TS--TSLEL

FA--AKATDSGTYICQLSNDVGTATSKATLF---

>Q8WZ42|DOMAIN_5788-5877

------------------------PQFIKKPSP--VLV----LRNGQ-ST

TFECQITGTPKIRVSWYLDGNE----------IT--A----I-------Q

K-H-G----------------------------IS-F-ID-GL--ATFQI

SG--ARVENSGTYVCEARNDAGTASCSIELK---

>Q8WZ42|DOMAIN_5882-5970

-------------------------PTFIRELK--PVE----VVKYS-DV

ELECEVTGTPPFEVTWLKNNRE----------IR--S----S-------K

K-Y-T----------------------------LT-D-RV-SV--FNLHI

TK--CDPSDTGEYQCIVSNEGGSCSCSTRVA---

>Q8WZ42|DOMAIN_5975-6063

-------------------------PSFIKKIE--NTT----TVLKS-SA

TFQSTVAGSPPISITWLKDDQI----------LD--E----D-------D

N-V-Y----------------------------IS-F-VD-SV--ATLQI

RS--VDNGHSGRYTCQAKNESGVERCYAFLL---

>Q8WZ42|DOMAIN_6067-6156

------------------------PAQIVEKAK--SVD----VTEKD-PM

TLECVVAGTPELKVKWLKDGKQ----------IV--P----S-------R

Y-F-S----------------------------MS-F-EN-NV--ASFRI

QS--VMKQDSGQYTFKVENDFGSSSCDAYLR---

>Q8WZ42|DOMAIN_6164-6252

-------------------------PSFTKKLT--KMD----KVLGS-SI

HMECKVSGSLPISAQWFKDGKE----------IS--T----S-------A

K-Y-R----------------------------LV-C-HE-RS--VSLEV

NN--LELEDTANYTCKVSNVAGDDACSGILT---

>Q8WZ42|DOMAIN_6257-6347

-------------------------PSFLVKPG--RQQ----AIPDS-TV

EFKAILKGTPPFKIKWFKDDVE----------LV--S----G-------P

K-C-F----------------------------IG-L-EG-ST--SFLNL

YS--VDASKTGQYTCHVTNDVGSDSCTTMLLVT-

>Q8WZ42|DOMAIN_6350-6440

------------------------PKFVKKLEA--SKI----VKAGD-SS

RLECKIAGSPEIRVVWFRNEHE----------LP--A----S-------D

K-Y-R----------------------------MT-F-ID-SV--AVIQM

NN--LSTEDSGDFICEAQNPAGSTSCSTKVIV--

>Q8WZ42|DOMAIN_6444-6534

-------------------------PVFSSFPP--IVE----TLKNA-EV

SLECELSGTPPFEVVWYKDKRQ----------LR--S----S-------K

K-Y-K----------------------------IA-S-KN-FH--TSIHI

LN--VDTSDIGEYHCKAQNEVGSDTCVCTVKLK-

>Q8WZ42|DOMAIN_6537-6626

-------------------------PRFVSKLN--SLT----VVAGE-PA

ELQASIEGAQPIFVQWLKEKEE----------VI-RE----S-------E

N-I-R----------------------------IT-F-VE-NV--ATLQF

AK--AEPANAGKYICQIKNDGGMEENMATLM---

>Q8WZ42|DOMAIN_6630-6721

------------------------PAVIVEKAG--PMT----VTVGE-TC

TLECKVAGTPELSVEWYKDGKL----------LT--S----S-------Q

K-H-K----------------------------FS-F-YN-KI--SSLRI

LS--VERQDAGTYTFQVQNNVGKSSCTAVVDVS-

>Q8WZ42|DOMAIN_6727-6815

-------------------------PSFTRRLK--NTG----GVLGA-SC

ILECKVAGSSPISVAWFHEKTK----------IV--S----G-------A

K-Y-Q----------------------------TT-F-SD-NV--CTLQL

NS--LDSSDMGNYTCVAANVAGSDECRAVLT---

>Q8WZ42|DOMAIN_6820-6908

-------------------------PSFVKEPE--PLE----VLPGK-NV

TFTSVIRGTPPFKVNWFRGARE----------LV--K----G-------D

R-C-N----------------------------IY-F-ED-TV--AELEL

FN--IDISQSGEYTCVVSNNAGQASCTTRLF---

>Q8WZ42|DOMAIN_6912-7001

------------------------PAAFLKRLS--DHS----VEPGK-SI

ILESTYTGTLPISVTWKKDGFN----------IT--T----S-------E

K-C-N----------------------------IV-T-TE-KT--CILEI

LN--STKRDAGQYSCEIENEAGRDVCGALVS---

>Q8WZ42|DOMAIN_7005-7093

------------------------PPYFVTELE--PLE----AAVGD-SV

SLQCQVAGTPEITVSWYKGDTK----------LR--P----T-------P

E-Y-R----------------------------TY-F-TN-NV--ATLVF

NK--VNINDSGEYTCKAENSIGTASSKTVF----

>Q8WZ42|DOMAIN_7102-7190

-------------------------PSFARQLK--DIE----QTVGL-PV

TLTCRLNGSAPIQVCWYRDGVL----------LR--D----D-------E

N-L-Q----------------------------TS-F-VD-NV--ATLKI

LQ--TDLSHSGQYSCSASNPLGTASSSARLT---

>Q8WZ42|DOMAIN_7198-7286

-------------------------PFFDIKPV--SID----VIAGE-SA

DFECHVTGAQPMRITWSKDNKE----------IR--P----G-------G

N-Y-T----------------------------IT-C-VG-NT--PHLRI

LK--VGKGDSGQYTCQATNDVGKDMCSAQLS---

>Q8WZ42|DOMAIN_7291-7380

------------------------PKFVKKLEA--SKV----AKQGE-SI

QLECKISGSPEIKVSWFRNDSE----------LH--E----S-------W

K-Y-N----------------------------MS-F-IN-SV--ALLTI

NE--ASAEDSGDYICEAHNGVGDASCSTALT---

>Q8WZ42|DOMAIN_7385-7473

-------------------------PVFTQKPS--PVG----ALKGS-DV

ILQCEISGTPPFEVVWVKDRKQ----------VR--N----S-------K

K-F-K----------------------------IT-S-KH-FD--TSLHI

LN--LEASDVGEYHCKATNEVGSDTCSCSVK---

>Q8WZ42|DOMAIN_7478-7567

-------------------------PRFVKKLS--DTS----TLIGD-AV

ELRAIVEGFQPISVVWLKDRGE----------VI-RE----S-------E

N-T-R----------------------------IS-F-ID-NI--ATLQL

GS--PEASNSGKYICQIKNDAGMRECSAVLT---

>Q8WZ42|DOMAIN_7571-7662

------------------------PARIIEKPE--PMT----VTTGN-PF

ALECVVTGTPELSAKWFKDGRE----------LS--A----D-------S

K-H-H----------------------------IT-F-IN-KV--ASLKI

PC--AEMSDKGLYSFEVKNSVGKSNCTVSVHVS-

>Q8WZ42|DOMAIN_7668-7756

-------------------------PSFIRKLK--DVN----AILGA-SV

VLECRVSGSAPISVGWFQDGNE----------IV--S----G-------P

K-C-Q----------------------------SS-F-SE-NV--CTLNL

SL--LEPSDTGIYTCVAANVAGSDECSAVLT---

>Q8WZ42|DOMAIN_7761-7849

-------------------------PSFEQTPD--SVE----VLPGM-SL

TFTSVIRGTPPFKVKWFKGSRE----------LV--P----G-------E

S-C-N----------------------------IS-L-ED-FV--TELEL

FE--VQPLESGDYSCLVTNDAGSASCTTHLF---

>Q8WZ42|DOMAIN_7853-7942

------------------------PATFVKRLA--DFS----VETGS-PI

VLEATYTGTPPISVSWIKDEYL----------IS--Q----S-------E

R-C-S----------------------------IT-M-TE-KS--TILEI

LE--STIEDYAQYSCLIENEAGQDICEALVS---

>Q8WZ42|DOMAIN_7946-8035

------------------------PPYFIEPLE--HVE----AVIGE-PA

TLQCKVDGTPEIRISWYKEHTK----------LR--S----A-------P

A-Y-K----------------------------MQ-F-KN-NV--ASLVI

NK--VDHSDVGEYSCKADNSVGAVASSAVLV---

>Q8WZ42|DOMAIN_8042-8133

------------------------PPFFARKLK--DVH----ETLGF-PV

AFECRINGSEPLQVSWYKDGVL----------LK--D----D-------A

N-L-Q----------------------------TS-F-VH-NV--ATLQI

LQ--TDQSHIGQYNCSASNPLGTASSSAKLILS-

>Q8WZ42|DOMAIN_8138-8229

------------------------PPFFDLKPV--SVD----LALGE-SG

TFKCHVTGTAPIKITWAKDNRE----------IR--P----G-------G

N-Y-K----------------------------MT-L-VE-NT--ATLTV

LK--VGKGDAGQYTCYASNIAGKDSCSAHLGVQ-

>Q8WZ42|DOMAIN_8232-8321

------------------------PRFIKKLEP--SRI----VKQDE-FT

RYECKIGGSPEIKVLWYKDETE----------IQ--E----S-------S

K-F-R----------------------------MS-F-VD-SV--AVLEM

HN--LSVEDSGDYTCEAHNAAGSASSSTSLK---

>Q8WZ42|DOMAIN_8326-8414

-------------------------PIFRKKPH--PIE----TLKGA-DV

HLECELQGTPPFHVSWYKDKRE----------LR--S----G-------K

K-Y-K----------------------------IM-S-EN-FL--TSIHI

LN--VDAADIGEYQCKATNDVGSDTCVGSIA---

>Q8WZ42|DOMAIN_8419-8508

-------------------------PRFVKKLS--DIS----TVVGK-EV

QLQTTIEGAEPISVVWFKDKGE----------IV-RE----S-------D

N-I-W----------------------------IS-Y-SE-NI--ATLQF

SR--VEPANAGKYTCQIKNDAGMQECFATLS---

>Q8WZ42|DOMAIN_8512-8603

------------------------PATIVEKPE--SIK----VTTGD-TC

TLECTVAGTPELSTKWFKDGKE----------LT--S----D-------N

K-Y-K----------------------------IS-F-FN-KV--SGLKI

IN--VAPSDSGVYSFEVQNPVGKDSCTASLQVS-

>Q8WZ42|DOMAIN_8609-8697

-------------------------PSFTRKLK--ETN----GLSGS-SV

VMECKVYGSPPISVSWFHEGNE----------IS--S----G-------R

K-Y-Q----------------------------TT-L-TD-NT--CALTV

NM--LEESDSGDYTCIATNMAGSDECSAPLT---

>Q8WZ42|DOMAIN_8702-8790

-------------------------PSFVQKPD--PMD----VLTGT-NV

TFTSIVKGTPPFSVSWFKGSSE----------LV--P----G-------D

R-C-N----------------------------VS-L-ED-SV--AELEL

FD--VDTSQSGEYTCIVSNEAGKASCTTHLY---

>Q8WZ42|DOMAIN_8794-8883

------------------------PAKFVKRLN--DYS----IEKGK-PL

ILEGTFTGTPPISVTWKKNGIN----------VT--P----S-------Q

R-C-N----------------------------IT-T-TE-KS--AILEI

PS--STVEDAGQYNCYIENASGKDSCSAQIL---

>Q8WZ42|DOMAIN_8888-8976

-------------------------PYFVKQLE--PVK----VSVGD-SA

SLQCQLAGTPEIGVSWYKGDTK----------LR--P----T-------T

T-Y-K----------------------------MH-F-RN-NV--ATLVF

NQ--VDINDSGEYICKAENSVGEVSASTFLT---

>Q8WZ42|DOMAIN_8984-9074

-------------------------PSFSRQLR--DVQ----ETVGL-PV

VFDCAISGSEPISVSWYKDGKP----------LK--D----S-------P

N-V-Q----------------------------TS-F-LD-NT--ATLNI

FK--TDRSLAGQYSCTATNPIGSASSSARLILT-

>Q8WZ42|DOMAIN_9079-9168

------------------------PPFFDIRLA--PVD----AVVGE-SA

DFECHVTGTQPIKVSWAKDSRE----------IR--S----G-------G

K-Y-Q----------------------------IS-Y-LE-NS--AHLTV

LK--VDKGDSGQYTCYAVNEVGKDSCTAQLN---

>Q8WZ42|DOMAIN_9176-9265

------------------------PSFTKRLSE--TVE----ETEGN-SF

KLEGRVAGSQPITVAWYKNNIE----------IQ--P----T-------S

N-C-E----------------------------IT-F-KN-NT--LVLQV

RK--AGMNDAGLYTCKVSNDAGSALCTSSIV---

>Q8WZ42|DOMAIN_9272-9361

------------------------PPVFDQHLT--PVT----VSEGE-YV

QLSCHVQGSEPIRIQWLKAGRE----------IK--P----S-------D

R-C-S----------------------------FS-F-AS-GT--AVLEL

RD--VAKADSGDYVCKASNVAGSDTTKSKVT---

>Q8WZ42|DOMAIN_9366-9470

PA----------VAPATKkaavdgRLFFVSEPQ--SIR----VVEKT-TA

TFIAKVGGDPIPNVKWTKGKWR----------QL--N----Q-------G

G-R-V----------------------------FI-H-QKGDE--AKLEI

RD--TTKTDSGLYRCVAFNEHGEIESNVNLQ---

>Q8WZ42|DOMAIN_9660-9755

PH----------IASAKltvi---EPAWERHLQ--DVT----LKEGQ-TC

TMTCQFSV-PNVKSEWFRNGRI----------LK--P----Q-------G

R-H-K----------------------------TE-V-EH-KV--HKLTI

AD--VRAEDQGQYTCKYEDLETSAELR-------

>Q8WZ42|DOMAIN_9760-9851

------------------------PIQFTKRIQ--NIV----VSEHQ-SA

TFECEVSF-DDAIVTWYKGPTE----------LT--E----S-------Q

K-Y-N----------------------------FR-N-DG-RC--HYMTI

HN--VTPDDEGVYSVIARLEPRGEARSTAELylt

>Q8WZ42|DOMAIN_12041-12133

------------------------PLKFVKEIK--DIIltesEFVGS-SA

IFECLVSP-STAITTWMKDGSN----------IR--E----S-------P

K-H-R----------------------------FI-A-DG-KD--RKLHI

ID--VQLSDAGEYTCVLRLGNKEKTSTAKLV---

>Q8WZ42|DOMAIN_12138-12222

P-----------------------VRFVKTLEE--EVT----VVKGQ-PL

YLSCELNK--ERDVVWRKDGKI----------VV--E----K-------P

G-R-Ivpg-------------------------VI-G-L---M--RALTI

ND--ADDTDAGTYTVTVENANNLECS--------

>Q8WZ42|DOMAIN_12233-12318

-------------------------DWLVKPIR--DQH----VKPKG-TA

IFACDIAK-DTPNIKWFKGYDE----------IP--AEpnDK--------

--T-E----------------------------IL-R-DG-NH--LYLKI

KN--AMPEDIAEYAVEIEGKRYPAKLT-------

>Q8WZ42|DOMAIN_12499-12584

-----------------------IRLKFMSPLE--DQT----VKEGE-TA

TFVCELSH-EKMHVVWFKNDAK----------LH--T----S-------R

T-V-L----------------------------IS-S-EG-KT--HKLEM

KE--VTLDDISQIKAQVKELSSTAQLK-------

>Q8WZ42|DOMAIN_12590-12672

-------------------------PYFTVKLH--DKT----AVEKD-EI

TLKCEVSK--DVPVKWFKDGEE----------IV--P----S-------P

K-Y-S----------------------------IK-A-DG-LR--RILKI

KK--ADLKDKGEYVCDCGTDKTKANVT-------

>Q8WZ42|DOMAIN_12766-12850

------------------------PLIFITPLS--DVK----VFEKD-EA

KFECEVSR-EPKTFRWLKGTQE----------IT--GD--DR--------

--F-E----------------------------LI-K-DG-TK--HSMVI

KS--AAFEDEAKYMFEAEDKHTSGKLI-------

>Q8WZ42|DOMAIN_12945-13032

-------------------------PYFTGKLQ--DYT----GVEKD-EV

ILQCEISK-ADAPVKWFKDGKE----------IK--P----S-------K

N-A-V----------------------------IK-A-DG-KK--RMLIL

KK--ALKSDIGQYTCDCGTDKTSGKLDIEDR---

>Q8WZ42|DOMAIN_13120-13206

----------------------PRVIGLLRPLK--DVT----VTAGE-TA

TFDCELSY-EDIPVEWYLKGKK----------LE--P----S-------D

K-V-V----------------------------PR-S-EG-KV--HTLTL

RD--VKLEDAGEVQLTAKDFKTHANLF-------

>Q8WZ42|DOMAIN_13210-13295

p-----------------------PVEFTKPLE--DQT----VEEGA-TA

VLECEVSR-ENAKVKWFKNGTE----------IL--K----S-------K

K-Y-E----------------------------IV-A-DG-RV--RKLVI

HD--CTPEDIKTYTCDAKDFKTSCNLN-------

>Q8WZ42|DOMAIN_13299-13384

p-----------------------HVEFLRPLT--DLQ----VREKE-MA

RFECELSR-ENAKVKWFKDGAE----------IK--K----G-------K

K-Y-D----------------------------II-S-KG-AV--RILVI

NK--CLLDDEAEYSCEVRTARTSGMLT-------

>Q8WZ42|DOMAIN_13388-13478

-----------------------EEAVFTKNLA--NIE----VSETD-TI

KLVCEVSK-PGAEVIWYKGDEE----------II--E----T-------G

R-Y-E----------------------------IL-T-EG-RK--RILVI

QN--AHLEDAGNYNCRLPSSRTDGKVKVHEla--

>Q8WZ42|DOMAIN_13479-13562

-------------------------AEFISKPQ--NLE----ILEGE-KA

EFVCSISK-ESFPVQWKRDDKT----------LE--S----G-------D

K-Y-D----------------------------VI-A-DG-KK--RVLVV

KD--ATLQDMGTYVVMVGAARAAAHLT-------

>Q8WZ42|DOMAIN_13565-13655

-----------------------EKLRIVVPLK--DTR----VKEQQ-EV

VFNCEVNT-EGAKAKWFRNEEA----------IF--D----S-------S

K-Y-I----------------------------IL-Q-KD-LV--YTLRI

RD--AHLDDQANYNVSLTNHRGENVKSAANLI--

>Q8WZ42|DOMAIN_13659-13748

-----------------------EDLRIVEPLK--DIE----TMEKK-SV

TFWCKVNR-LNVTLKWTKNGEE----------VP--FD--NR-------V

S-Y-R----------------------------VD-K-Y---K--HMLTI

KD--CGFPDEGEYIVTAGQDKSVAELLIIEa---

>Q8WZ42|DOMAIN_13749-13833

------------------------PTEFVEHLE--DQT----VTEFD-DA

VFSCQLSR-EKANVKWYRNGRE----------IK--E----G-------K

K-Y-K----------------------------FE-K-DG-SI--HRLII

KD--CRLDDECEYACGVEDRKSRARLF-------

>Q8WZ42|DOMAIN_13927-14012

-------------------------PKIKTADQ--DLV----VDVGK-PL

TMVVPYDAYPKAEAEWFKENEP----------LS--T----K--------

----T----------------------------ID-T-TA-EQ--TSFRI

LE--AKKGDKGRYKIVLQNKHGKAEGFINLK---

>Q8WZ42|DOMAIN_14615-14708

----------------------PEIFLDVKLLA--GLT----VKAGT-KI

ELPATVTGKPEPKITWTKADMI----------LK--Q----D-------K

R-I-T----------------------------IE-N-VP-KK--STVTI

VD--SKRSDTGTYIIEAVNVCGRATAVVEVNVL-

>Q8WZ42|DOMAIN_15314-15402

-------------------------PTIDLETH--DII----VIEGE-KL

SIPVPFRAVPVPTVSWHKDGKE----------VK--A----S-------D

R-L-T----------------------------MK-N-DH-IS--AHLEV

PK--SVRADAGIYTITLENKLGSATASINVK---

>Q8WZ42|DOMAIN_15608-15724

--------------------------PKVILRT--SLE----VKRGD-EI

ALDASISGSPYPTITWIKDENV----------IV--PE--E---------

I-K-KraaplvrrrkgevqeeepfvlpltqrlsID-N-SKKGE--SQLRV

RD--SLRPDHGLYMIKVENDHGIAKAPCTVS---

>Q8WZ42|DOMAIN_16029-16119

----------------------PAVELDVSVKG--GIQ----IMAGK-TL

RIPAVVTGRPVPTKVWTKEEGE----------LD--K------------D

R-V-V----------------------------ID-N-VG-TK--SELII

KD--ALRKDHGRYVITATNSCGSKFAAARVE---

>Q8WZ42|DOMAIN_16322-16420

P---------------------TIKLRLSVRGD--TIK----VKAGE-PV

HIPADVTGLPMPKIEWSKNETV----------IE--K----PTdalqi-T

K-E-E----------------------------VS-R-SE-AK--TELSI

PK--AVREDKGTYTVTASNRLGSVFRNVHVE---

>Q8WZ42|DOMAIN_16727-16834

--------------PATdiQEEPEVFIDIGAQD--CLV----CKAGS-QI

RIPAVIKGRPTPKSSWEFDGKAkkamkdgvhdIP------ED-------A

Q-L-E----------------------------TA-E-N---S--SVIII

PE--CKRSHTGKYSITAKNKAGQKTANCRVK---

>Q8WZ42|DOMAIN_17044-17139

-------------------------PPSIDLKE--FME----VEEGT-NV

NIVAKIKGVPFPTLTWFKAPPK----------KP--D----NkepvlyDT

H-V-N----------------------------KL-V-VD-DT--CTLVI

PQ--SRRSDTGLYTITAVNNLGTASKEMRLN---

>Q8WZ42|DOMAIN_17449-17536

----------------------PDLQLDASVRD--RIV----VHAGG-VI

RIIAYVSGKPPPTVTWNMNERT----------LP------QE-------A

T-I-E----------------------------TT-A-I---S--SSMVI

KN--CQRSHQGVYSLLAKNEAGERKKTII-----

>Q8WZ42|DOMAIN_17745-17834

------------------------PTLHLDFRD--KLT----IRVGE-AF

ALTGRYSGKPKPKVSWFKDEAD----------VL--E----D-------D

R-T-H----------------------------IK-T-TP-AT--LALEK

IK--AKRSDSGKYCVVVENSTGSRKGFCQVN---

>Q8WZ42|DOMAIN_18143-18228

----------------------PELILDANMAR--EQH----IKVGD-TL

RLSAIIKGVPFPKVTWKKEDRD----------AP--T----K-------A

R-I-D----------------------------VT-P-V---G--SKLEI

RN--AAHEDGGIYSLTVENPAGSKTVS-------

>Q8WZ42|DOMAIN_18435-18526

----------------------PSVELDVKLIE--GLV----VKAGT-TV

RFPAIIRGVPVPTAKWTTDGSE----------IK--TD--EH-------Y

T-V-E----------------------------TD-N-F---S--SVLTI

KN--CLRRDTGEYQITVSNAAGSKTVAVHLT---

>Q8WZ42|DOMAIN_18833-18924

----------------------PEVELDVTCRD--VIT----VRVGQ-TI

RILARVKGRPEPDITWTKEGKV----------LV--R----E-------K

R-V-D----------------------------LI-Q-DL-PR--VELQI

KE--AVRADHGKYIISAKNSSGHAQGSAIVN---

>Q8WZ42|DOMAIN_19128-19219

------------------------PVLDLKLSG--VLT----VKAGD-TI

RLEAGVRGKPFPEVAWTKDKDA----------TDLTR----S-------P

R-V-K----------------------------ID-T-RA-DS--SKFSL

TK--AKRSDGGKYVVTATNTAGSFVAYATVN---

>Q8WZ42|DOMAIN_19531-19617

----------------------PEIDLDASMRK--LVI----VRAGC-PI

RLFAIVRGRPAPKVTWRKVGID----------NV-VR----K-------G

Q-V-D----------------------------LV-D-T---M--AFLVI

PN--STRDDSGKYSLTLVNPAGEKAVF-------

>Q8WZ42|DOMAIN_19826-19914

-------------------------PPKILMPE--QIT----IKAGK-KL

RIEAHVYGKPHPTCKWKKGEDE----------VV--T----S-------S

H-L-A----------------------------VH-K-AD-SS--SILII

KD--VTRKDSGYYSLTAENSSGTDTQKIKVV---

>Q8WZ42|DOMAIN_20220-20311

----------------------PRIDLSVAMKS--LLT----VKAGT-NV

CLDATVFGKPMPTVSWKKDGTL----------LK--PA--E---------

G-I-K----------------------------MA-M-QR-NL--CTLEL

FS--VNRKDSGDYTITAENSSGSKSATIKLK---

>Q8WZ42|DOMAIN_20893-20996

pseitvvarddvV--------APDLDLKGLPDL--CYL----AKENS-NF

RLKIPIKGKPAPSVSWKKGEDP----------LA-------T------DT

R-V-S----------------------------VE-S-SA-VN--TTLIV

YD--CQKSDAGKYTITLKNVAGTKEGTIS-----

>Q8WZ42|DOMAIN_21303-21395

P---------------------TIVLDPTIKDG--LTI----KAGDT-IV

LNAISILGKPLPKSSWSKAGKD----------IR--P----S-------D

I-T-Q----------------------------IT-S-TP-TS--SMLTI

KY--ATRKDAGEYTITATNPFGTKVEHVKVT---

>Q8WZ42|DOMAIN_21701-21793

----------------------PDFELDAELRR--TLV----VRAGL-SI

RIFVPIKGRPAPEVTWTKDNIN----------LK--N----R-------A

N-I-E----------------------------NT-E-S---F--TLLII

PE--CNRYDTGKFVMTIENPAGKKSGFVNVRVLd

>Q8WZ42|DOMAIN_21990-22083

----------------------PELDLRGIYQK--LVI----AKAGD-NI

KVEIPVLGRPKPTVTWKKGDQI----------LK--Q----T-------Q

R-V-N----------------------------FE-T-TA-TS--TILNI

NE--CVRSDSGPYPLTARNIVGEVGDVITIQVh-

>Q8WZ42|DOMAIN_22386-22477

----------------------PKIKVDVKFKD--TVI----LKAGE-AF

RLEADVSGRPPPTMEWSKDGKE----------LE--G----T-------A

K-L-E----------------------------IK-I-AD-FS--TNLVN

KD--STRRDSGAYTLTATNPGGFAKHIFNVK---

>Q8WZ42|DOMAIN_22785-22874

----------------------PEIELDADLRK--VVT----IRACC-TL

RLFVPIKGRPAPEVKWARDHGE----------SL--D----K-------A

S-I-E----------------------------ST-S-S---Y--TLLIV

GN--VNRFDSGKYILTVENSSGSKSAFVNVR---

>Q8WZ42|DOMAIN_23075-23163

-------------------------PAFKLLFN--TFT----VLAGE-DL

KVDVPFIGRPTPAVTWHKDNVP----------LK--Q----T-------T

R-V-N----------------------------AE-S-TE-NN--SLLTI

KD--ACREDVGHYVVKLTNSAGEAIETLNVI---

>Q8WZ42|DOMAIN_23468-23555

----------------------PRISMDPKYKD--TIV----VHAGE-SF

KVDADIYGKPIPTIQWIKGDQE----------LS--N----T-------A

R-L-E----------------------------IK-S-TD-FA--TSLSV

KD--AVRVDSGNYILKAKNVAGERSVT-------

>Q8WZ42|DOMAIN_23867-23954

----------------------PDIDLDLELRK--IIN----IRAGG-SL

RLFVPIKGRPTPEVKWGKVDGE----------IR--D----A-------A

I-I-D----------------------------VT-S-S---F--TSLVL

DN--VNRYDSGKYTLTLENSSGTKSAFVT-----

>Q8WZ42|DOMAIN_24157-24241

-------------------------PDVKPAFS--SYS----VQVGQ-DL

KIEVPISGRPKPTITWTKDGLP----------LK--Q----T-------T

R-I-N----------------------------VT-D-SL-DL--TTLSI

KE--THKDDGGQYGITVANVVGQKTAS-------

>Q8WZ42|DOMAIN_24550-24641

----------------------PRISMDPKFRD--TIV----VNAGE-TF

RLEADVHGKPLPTIEWLRGDKE----------IE--E----S-------A

R-C-E----------------------------IK-N-TD-FK--ALLIV

KD--AIRIDGGQYILRASNVAGSKSFPVNVK---

>Q8WZ42|DOMAIN_24949-25038

----------------------PELDLDSELRK--GIV----VRAGG-SA

RIHIPFKGRPTPEITWSREEGE----------FT--D----K-------V

Q-I-E----------------------------KG-V-N---Y--TQLSI

DN--CDRNDAGKYILKLENSSGSKSAFVTVK---

>Q8WZ42|DOMAIN_25239-25325

-------------------------PSLKLPFN--TYS----IQAGE-DL

KIEIPVIGRPRPNISWVKDGEP----------LK--Q----T-------T

R-V-N----------------------------VE-E-TA-TS--TVLHI

KE--GNKDDFGKYTVTATNSAGTATENLS-----

>Q8WZ42|DOMAIN_25632-25722

----------------------PNASLDPKYKD--VIV----VHAGE-TF

VLEADIRGKPIPDVVWSKDGKE----------LE--E----T------AA

R-M-E----------------------------IK-S-TI-QK--TTLVV

KD--CIRTDGGQYILKLSNVGGTKSIPIT-----

>Q8WZ42|DOMAIN_26032-26121

----------------------PEIELDADLRK--VVV----LRASA-TL

RLFVTIKGRPEPEVKWEKAEGI----------LT--D----R-------A

Q-I-E----------------------------VT-S-S---F--TMLVI

DN--VTRFDSGRYNLTLENNSGSKTAFVNVR---

>Q8WZ42|DOMAIN_26322-26410

-------------------------PSVELPFH--TFN----VKARE-QL

KIDVPFKGRPQATVNWRKDGQT----------LK--E----T-------T

R-V-N----------------------------VS-S-SK-TV--TSLSI

KE--ASKEDVGTYELCVSNSAGSITVPITII---

>Q8WZ42|DOMAIN_26714-26801

----------------------PRVMMDVKFRD--VIV----VKAGE-VL

KINADIAGRPLPVISWAKDGIE----------IE--E----R-------A

R-T-E----------------------------II-S-TD-NH--TLLTV

KD--CIRRDTGQYVLTLKNVAGTRSVA-------

>Q8WZ42|DOMAIN_27101-27196

PQ----------IAKER--EEEPLFDIDSEMRK--TLI----VKAGA-SF

TMTVPFRGRPVPNVLWSKPDTD----------LR--T----R-------A

Y-V-D----------------------------TT-D-S---R--TSLTI

EN--ANRNDSGKYTLTIQNVLSAASLT-------

>Q8WZ42|DOMAIN_27797-27888

----------------------PVIDLPLEYTE--VVK----YRAGT-SV

KLRAGISGKPAPTIEWYKDDKE----------LQ--T----N-------A

L-V-C----------------------------VE-N-TT-DL--ASILI

KD--ADRLNSGCYELKLRNAMGSASATIRVQ---

>Q8WZ42|DOMAIN_28196-28286

----------------------PEIDLDVALRT--SVI----AKAGE-DV

QVLIPFKGRPPPTVTWRKDEKN----------LG--SD--AR-------Y

S-I-E----------------------------NT-D-S---S--SLLTI

PQ--VTRNDTGKYILTIENGVGEPKSSTVS----

>Q8WZ42|DOMAIN_28488-28577

----------------------PEVDLSDIPGA--QVT----VRIGH-NV

HLELPYKGKPKPSISWLKDGLP----------LK--E----S-------E

F-V-R----------------------------FS-K-TE-NK--ITLSI

KN--AKKEHGGKYTVILDNAVCRIAVPIT-----

>Q8WZ42|DOMAIN_28882-28974

P---------------------PIVEFGPEYFD--GLI----IKSGE-SL

RIKALVQGRPVPRVTWFKDGVE----------IE--K----R-------M

N-M-E----------------------------IT-D-VL-GS--TSLFV

RD--ATRDHRGVYTVEAKNASGSAKAEIKVK---

>Q8WZ42|DOMAIN_29282-29367

----------------------PELDIDANFKQ--THV----VRAGA-SI

RLFIAYQGRPTPTAVWSKPDSN----------LS--L----R-------A

D-I-H----------------------------TT-D-S---F--STLTV

EN--CNRNDAGKYTLTVENNSGSKSIT-------

>Q8WZ42|DOMAIN_29568-29663

PQ----------I--------EPTADLTGITNQ--LIT----CKAGS-PF

TIDVPISGRPAPKVTWKLEEMR----------LK--E----T-------D

R-V-S----------------------------IT-T-TK-DR--TTLTV

KD--SMRGDSGRYFLTLENTAGVKTFSVTVV---

>Q8WZ42|DOMAIN_29971-30059

P---------------------KAELDARLHGD--LVT----IRAGS-DL

VLDAAVGGKPEPKIIWTKGDKE----------LDLcE----K-------V

S-L-Q----------------------------YT-G-K---R--ATAVI

KF--CDRSDSGKYTLTVKNASGTKAVS-------

>Q8WZ42|DOMAIN_30371-30460

----------------------PDLELADDLKK--TVT----IRAGA-SL

RLMVSVSGRPPPVITWSKQGID----------LA--S----R-------A

I-I-D----------------------------TT-E-S---Y--SLLIV

DK--VNRYDAGKYTIEAENQSGKKSATVLVK---

>Q8WZ42|DOMAIN_30663-30754

----------------------PTIDLSTMPQK--TIH----VPAGR-PV

ELVIPIAGRPPPAASWFFAGSK----------LR--E----S-------E

R-V-T----------------------------VE-T-HT-KV--AKLTI

RE--TTIRDTGEYTLELKNVTGTTSETIKVI---

>Q8WZ42|DOMAIN_31061-31150

----------------------PDYELDERYQE--GIF----VRQGG-VI

RLTIPIKGKPFPICKWTKEGQD----------IS--K----R-------A

M-I-A----------------------------TS-E-T---H--TELVI

KE--ADRGDSGTYDLVLENKCGKKAVYIKVR---

>Q8WZ42|DOMAIN_31460-31548

-------------------------PGIRKEMK--DVT----TKLGE-AA

QLSCQIVGRPLPDIKWYRFGKE----------LI--Q----S-------R

K-Y-K----------------------------MS-S-DG-RT--HTLTV

MT--EEQEDEGVYTCIATNEVGEVETSSKLL---

>Q8WZ42|DOMAIN_31855-31945

-------------------------PHFKEELR--NLN----VRYQS-NA

TLVCKVTGHPKPIVKWYRQGKE----------II--A----D-------G

LkyrI----------------------------QE-F-KG-GY--HQLII

AS--VTDDDATVYQVRATNQGGSVSGTASLE---

>Q8WZ42|DOMAIN_31955-32046

-------------------------PKTLEGMG--AVH----ALRGE-VV

SIKIPFSGKPDPVITWQKGQDL----------ID--NN--GH-------Y

Q-V------------------------------IV-T-RS-FT--SLVFP

NG--VERKDAGFYVVCAKNRFGIDQKTVELDVA-

>Q8WZ42|DOMAIN_32496-32584

-------------------------PVSGQIMH--AVG----EEGGH-VK

YVCKIENYDQSTQVTWYFGVRQ----------LE--N----S-------E

K-Y-E----------------------------IT-Y-ED-GV--AILYV

KD--ITKLDDGTYRCKVVNDYGEDSSYAELF---

>Q8WZ42|DOMAIN_32617-32710

-------------------------PEFTLPLY--NKT----AYVGE-NV

RFGVTITVHPEPHVTWYKSGQK----------IK--P----G-------D

N-D-K----------------------------KYtF-ES-DKGLYQLTI

NS--VTTDDDAEYTVVARNKYGEDSCKAKLTVT-

>Q8WZ42|DOMAIN_32722-32811

-------------------------PMFKRLLA--NAE----CQEGQ-SV

CFEIRVSGIPPPTLKWEKDGQP----------LS--L----G-------P

N-I-E----------------------------II-H-EGLDY--YALHI

RD--TLPEDTGYYRVTATNTAGSTSCQAHLQ---

>Q8WZ42|DOMAIN_33301-33391

-------------------------PRITLRMR--SHR----VPCGQ-NT

RFILNVQSKPTAEVKWYHNGVE----------LQ--E----S-------S

K-I-H----------------------------YT-N-TS-GV--LTLEI

LD--CHTDDSGTYRAVCTNYKGEASDYATLDVT-

>Q8WZ42|DOMAIN_33488-33576

-------------------------ARILTKPR--SMT----VYEGE-SA

RFSCDTDGEPVPTVTWLRKGQV----------LS--T----S-------A

R-H-Q----------------------------VT-T-TK-YK--STFEI

SS--VQASDEGNYSVVVENSEGKQEAEFTLT---

>Q8WZ42|DOMAIN_33645-33732

-------------------------PKITQFLK--AEA----SKEIA-KL

TCVVESSVLRAKEVTWYKDGKK----------LK--E----N-------G

H-F-Q----------------------------FH-Y-SA-D-GTYELKI

NN--LTESDQGEYVCEISGEGGTSKTNLQ-----

>Q8WZ42|DOMAIN_33779-33867

-------------------------PVIVTGLQ--DTT----VSSDS-VA

KFAVKATGEPRPTAIWTKDGKA----------IT--Q----G-------G

K-Y-K----------------------------LS-E-DK-GG--FFLEI

HK--TDTSDSGLYTCTVKNSAGSVSSSCKLT---

>Q8WZ42|DOMAIN_33963-34052

----------------------RTHAEIKAFST--QMS----INEGQ-RL

VLKANIAG--ATDVKWVLNGVE----------LT--N----S-------E

E-Y-R----------------------------YG-V-SG-SD--QTLTI

KQ--ASHRDEGILTCISKTKEGIVKCQYDLT---

>Q8WZ42|DOMAIN_34061-34149

-------------------------PAFISQPR--SQN----INEGQ-NV

LFTCEISGEPSPEIEWFKNNLP----------IS--I----S-------S

N-V-S----------------------------IS-R-SR-NV--YSLEI

RN--ASVSDSGKYTIKAKNFRGQCSATASLM---
