# Supplementary material for: Mechanical Network in Titin Immunoglobulin from Force Distribution Analysis
Source: PLoS Comput Biol. 2009 Mar 13;5(3):e1000306. doi: 10.1371/journal.pcbi.1000306 (PMC2643529; doi:10.1371/journal.pcbi.1000306)
Supplement: Dataset S2 — Multiple sequence alignment - IGdiverse alignment (0.34 MB DOC) [file pcbi.1000306.s002.doc]

>1WAA|DOMAIN_5253-5341Iglike1

l---I-EVE----KPLYGVEVF-VGETAHF-EIELS--E-P--DVH-G-Q

---WKL-K-----GQPLA-A------------------------------

---S---P-D--CE--I--I-E--D--G----------------------

------------------K--KHILILHNCQLG-MTGEVSFQA-ANT-KS

AANLKVKEL-----

>default|A2ASS5_MOUSE/52135297.0|A2ASS5_MOUSE/52135297

----I-KVE----KPLYGVEVF-VGETARF-EIELS--E-P--DVH-G-Q

---WKL-K-----GEPLT-A------------------------------

---S---P-D--CE--I--I-E--D--G----------------------

------------------K--KHVLVLYNCQLD-MTGEISFQA-ANA-KS

AANLKV--------

>default|Q4RE91_TETNG/729813.0|Q4RE91_TETNG/729813

----I-KVL----RPMYGAEVF-EGETARF-EVELS--E-D--DVH-G-Q

---WSL-N-----GELLS-P------------------------------

---S---S-D--IE--I--V-E--D--G----------------------

------------------P--KHTLVLYNCRVP-QTGEVVFTA-ANA-KC

SANLKV--------

>default|A2ASS5_MOUSE/55695653.0|A2ASS5_MOUSE/55695653

----I-KLV----RPLYSVEVM-ETETARF-ETEIS--E-D--DIH-A-N

---WKL-K-----GEALL-Q------------------------------

---T---P-E--CE--I--K-E--E--G----------------------

------------------K--IHVLILHNCRLD-QTGGVDFQA-ANV-KS

SAHLRV--------

>default|Q7ZZ46_BRARE/10111095.0|Q7ZZ46_BRARE/10111095

----I-KIS----RPLYSVEVT-ETETARF-ETEIS--E-E--DVH-G-H

---WKL-K-----GDALH-Q------------------------------

---S---P-D--CE--I--K-E--E--G----------------------

------------------T--KHMLILYNVRMD-MAGAVDFSA-ANA-KS

TAQLRV--------

>default|Q7ZZ46_BRARE/317401.0|Q7ZZ46_BRARE/317401

----A-EIL----KPLASVEVV-EKTEANF-ETEIS--E-D--DVP-G-E

---WKL-Q-----GQVLT-R------------------------------

---S---P-T--CD--I--R-S--E--G----------------------

------------------T--KRFLTIKNVQLD-QAGEVTYQA-LNA-VT

SAMLNV--------

>default|Q7YRF6_CANFA/15411625.0|Q7YRF6_CANFA/15411625

----V-ELL----KPIEDVTIY-EKESASF-DAEIS--E-A--DIP-G-Q

---WKL-K-----GELLR-P------------------------------

---S---P-T--CE--I--K-A--E--G----------------------

------------------G--KRFLTLHKVKLD-QAGEVLYQA-LNA-FT

TAILTV--------

>default|Q80TU8_MOUSE/605689.0|Q80TU8_MOUSE/605689

----V-SVL----RELRSVSAR-EGDGATF-ECTVS--E-T--EIT-G-R

---WEL-G-----GRALR-P------------------------------

---G---G-R--VR--I--R-Q--E--G----------------------

------------------K--KHILVLSELRTE-DTGEVCFQA-GPA-QS

LARLEV--------

>default|TITIN_HUMAN/1312313207.0|TITIN_HUMAN/1312313207

----I-GLL----RPLKDVTVT-AGETATF-DCELS--Y-E--DIP-V-E

---WYL-K-----GKKLE-P------------------------------

---S---D-K--VV--P--R-S--E--G----------------------

------------------K--VHTLTLRDVKLE-DAGEVQLTA-KDF-KT

HANLFV--------

>default|Q4SLN8_TETNG/776863.0|Q4SLN8_TETNG/776863

----I-TIV----QGLENVEIF-NGGEALFECALSR--P-E--SKD-C-R

---WLL-D-----GKPVK-E------------------------------

---S---P-T--TEI-V-SF-E--N--G----------------------

------------------R--RHLLLMKELSVK-DTCTVTFKA-GTA-ST

SAQLSV--------

>default|Q4RE90_TETNG/99183.0|Q4RE90_TETNG/99183

----V-EFT----KPLHDVEVR-EKESARF-ECEVS--R-E--NIK-V-R

---WFK-D-----GSEIR-K------------------------------

---G---K-K--YE--I--I-A--Q--G----------------------

------------------R--QHVLIVHKSVFD-DEAEYECDA-KTS-KS

SGMLTV--------

>default|Q7ZWM9_XENLA/267351.0|Q7ZWM9_XENLA/267351

----V-EFV----TELQNVTVL-EGETASF-KCMVS--P-D--NVD-L-R

---WEL-N-----GSPVY-S------------------------------

---D---K-R--F------S-TTNN--G----------------------

------------------M--CHSLILHSCRLS-DSGKITANA-EGL-IS

RAKLRV--------

>default|Q4SM88_TETNG/15651649.0|Q4SM88_TETNG/15651649

----I-CIR----KALTDQETY-ERETASF-EVELS--H-T--DVE-G-I

---WQK-D-----GIRVK-P------------------------------

---N---N-Q--WR--V--S-T--N--G----------------------

------------------R--VHSLTLSNLTLE-DTGSVVFSA-EGV-RT

TARLTV--------

>default|Q7ZWM9_XENLA/801886.0|Q7ZWM9_XENLA/801886

----I-QIS----RGLSDVRVK-APEDACFTCDVSL--P-L--SRP-A-Q

---WSL-N-----GEPLH-N------------------------------

---E---E-D--VS--I--E-T--R--G----------------------

------------------N--RHRLILRNTHPG-LSGTVRFTA-GKA-RS

EAKLTV--------

>default|Q7TNP4_MOUSE/697781.0|Q7TNP4_MOUSE/697781

----V-KLV----SELTPLTVH-EGDDATF-QCEVS--P-P--DAE-V-T

---WLR-N-----GAVIT-A------------------------------

---G---P-Q--LE--M--V-Q--N--G----------------------

------------------S--SRTLIIRGCQLK-DAGTVTARA-GAA-DT

SARLHV--------

>default|Q28633_RABIT/13471431.0|Q28633_RABIT/13471431

----G-AIS----KPLTDQTVA-ESQEAVF-ECEVA--N-P--DSE-G-E

---WLK-E-----GKHLP-L------------------------------

---S---N-A--VR--S--E-S--D--G----------------------

------------------H--KRRLIIAATKLD-DIGEYTYKV-ATS-KT

SAKLKV--------

>default|A2AAJ8_MOUSE/55139.0|A2AAJ8_MOUSE/55139

----V-QIT----KPLEDVEVM-EKEGATF-SCEVS--H-D--EVP-G-L

---WFR-E-----SSKLR-P------------------------------

---S---D-N--VR--I--R-Q--E--G----------------------

------------------R--TYTLIFRRVLAE-DAGEVKFVA-ENA-ES

RAHLRV--------

>default|Q98918_CHICK/23472431.0|Q98918_CHICK/23472431

----G-AIS----KPLADLTVA-ESQRAVF-ECEVA--N-P--ESE-G-Q

---WLK-N-----GKPLP-M------------------------------

---T---D-Q--YR--A--E-T--D--G----------------------

------------------V--KRRLNIPAAKMD-DMGEYSYEI-ASS-KT

SAKLHV--------

>default|Q7ZWM9_XENLA/623707.0|Q7ZWM9_XENLA/623707

P-----QML----KDLQDVDIA-ENENAVF-MCEVS--C-A--QAK-G-E

---WFK-N-----GEQVQ-V------------------------------

---S---N-T--TK--I--R-Q--E--G----------------------

------------------R--RHFLLICGAQCE-DAGEITFKA-RRL-QS

TARLRV--------

>default|OBSCN_HUMAN/42504336.0|OBSCN_HUMAN/42504336

----V-TIL----EPLQDVQLS-EGQDASFQCRLSR--A-S--GQE-A-R

---WAL-G-----GVPLQ-A------------------------------

---N---E-M--ND--I-TV-E--Q--G----------------------

------------------T--LHLLTLHKVTLE-DAGTVSFHV-GTC-SS

EAQLKV--------

>default|Q4RE92_TETNG/25492633.0|Q4RE92_TETNG/25492633

----I-KII----KKTKDVTTL-MDGTASF-EMSLS--H-D--DVP-V-K

---WMF-K-----GAELK-P------------------------------

---S---D-R--CK--I--L-R--E--R----------------------

------------------R--AHKLILQNVNSS-NAGEYTAVV-GHL-QC

SATLTV--------

>default|Q7ZZ48_BRARE/21032187.0|Q7ZZ48_BRARE/21032187

----I-KIV----KKPKDVTSL-LGATASF-ELSLS--H-D--DIP-V-K

---WML-N-----NQELT-T------------------------------

---S---S-N--IK--I--L-S--E--R----------------------

------------------K--AHKLIIQSVESD-LAGEYIAVI-GHL-QC

SAHLQV--------

>default|Q4T444_TETNG/16611745.0|Q4T444_TETNG/16611745

----V-GLV----RPLKDVMVT-AGETATF-ECELS--Y-E--GIV-V-E

---WFL-G-----GQKME-A------------------------------

---G---E-R--VK--T--R-V--A--G----------------------

------------------R--AHALTIRDVNLS-EAGEVKLTA-KDF-QT

QAHLIV--------

>default|TITIN_DROME/1703017114.0|TITIN_DROME/1703017114

P-----EIV----EPLNDVAVT-KGENAVF-EVELS--K-G--DAL-V-K

---WFK-N-----GKEIV-F------------------------------

---N---E-R--IQ--L--A-I--D--G----------------------

------------------K--KQSLRIVKAKPE-DVGEYSVQV-GEQ-TS

KAKLTV--------

>default|Q98918_CHICK/27852869.0|Q98918_CHICK/27852869

----I-EFR----KHIKDIKVV-EKKRAIF-ECEIS--E-P--DVQ-V-Q

---WMK-D-----GQELQ-I------------------------------

---G---D-R--MK--I--Q-R--E--K----------------------

------------------Y--VHRLIIPSTKMS-DAGQYTVVA-GGN-TS

SANLIV--------

>default|Q4TAD4_TETNG/15911675.0|Q4TAD4_TETNG/15911675

----V-QIT----KPMKDKECS-ESENVTF-EVDVS--H-P--GID-A-F

---WTF-N-----GQPLK-S------------------------------

---G---I-K--YK--M--E-S--K--D----------------------

------------------K--GHTLTIINAMKD-EEGEYMFAA-GEQ-TC

SATLTV--------

>default|Q4SLN7_TETNG/19212005.0|Q4SLN7_TETNG/19212005

----I-QVI----KGLTETTAH-ETETVTL-EIELS--Q-A--DVD-G-S

---WTK-D-----GVNVK-S------------------------------

---G---G-N--CR--I--T-A--L--G----------------------

------------------R--KQTLTLSNLKME-DAGTFVFQA-DGI-RV

SGKLTV--------

>default|Q7ZZ46_BRARE/744828.0|Q7ZZ46_BRARE/744828

----I-SFV----MPLSDVQVY-EKDEARF-EIELS--R-E--AKT-V-R

---WLK-G-----SQELK-V------------------------------

---D---E-K--FE--I--L-A--E--G----------------------

------------------K--RHILVVKSVAYE-DEAKYMFEA-EDK-RT

SAKLVI--------

>default|Q4TAD4_TETNG/21162200.0|Q4TAD4_TETNG/21162200

----I-EFT----KQLKDLNIT-EKKRATF-ECEVS--E-P--NVQ-V-M

---WMK-D-----GQELE-M------------------------------

---S---D-R--YK--I--S-W--D--Q----------------------

------------------F--THRLVLPSVRLS-DGGEYTAVA-GSN-MS

KANLGV--------

>default|A2ASS5_MOUSE/60146098.0|A2ASS5_MOUSE/60146098

----A-EFI----SKPQNLEIL-EGEKAEF-VCTIS--K-E--SFE-V-Q

---WKR-D-----DQTLE-S------------------------------

---G---D-K--YD--I--I-A--D--G----------------------

------------------K--KRVLVVKDATLQ-DMGTYVVMV-GAA-RA

AAHLTV--------

>default|Q4SM88_TETNG/13871471.0|Q4SM88_TETNG/13871471

----V-EFL----TELHNTTVL-EGEDATF-KCVVS--P-D--DVH-L-V

---WLM-D-----NEPIA-L------------------------------

---G---D-R--Y------Q-ATQN--G----------------------

------------------L--CHTLVIKKCQML-DCSKITAEA-EGQ-IS

KAILKV--------

>default|Q8T101_BOMMO/23332417.0|Q8T101_BOMMO/23332417

P-----EII----TRLQNQKVN-KGNKATF-EIELT--K-G--DAL-V-R

---WFK-D-----GSEIQ-F------------------------------

---S---N-H--IQ--L--T-I--D--G----------------------

------------------K--KQKLKIYDCDLD-DAGEYSCEV-GNS-KC

TAILTV--------

>default|OBSCN_HUMAN/25582642.0|OBSCN_HUMAN/25582642

----V-TVT----GPLQDAEAT-EEGWASF-SCELS--H-E--DEE-V-E

---WSL-N-----GMPLY-N------------------------------

---D---S-F--HE--I--S-H--K--G----------------------

------------------R--RHTLVLKSIQRA-DAGIVRASS-LKV-ST

SARLEV--------

>default|Q4SM88_TETNG/17431827.0|Q4SM88_TETNG/17431827

----L-EIM----QDLEDLYIQ-EDQNAVF-MCEVS--L-E--EVA-G-E

---WYK-D-----GHKIR-P------------------------------

---S---S-T--IK--I--R-S--E--G----------------------

------------------T--KHFLLMCNVKAE-DAGEIRFTA-RDV-ES

IAYLEV--------

>default|OBSCN_HUMAN/43404427.0|OBSCN_HUMAN/43404427

----N-TVV----RGLENVEAL-EGGEALFECQLSQ--P-E--VAA-H-T

---WLL-D-----DEPVH-T------------------------------

---S---E-N--AEV-V-FF-E--N--G----------------------

------------------L--RHLLLLKNLRPQ-DSCRVTFLA-GDM-VT

SAFLTV--------

>default|Q7ZZ48_BRARE/18411925.0|Q7ZZ48_BRARE/18411925

----V-AII----KHMEDCVCT-ETQNVTF-EVEVS--H-P--GID-A-L

---WTF-K-----GQALK-A------------------------------

---G---P-K--YK--I--E-S--K--G----------------------

------------------T--HYSLTVLNAMKD-EEGEYVFMA-GEK-ES

RARLIV--------

>default|Q6P6L5_MOUSE/343431.0|Q6P6L5_MOUSE/343431

----I-MVT----KQLEDMNAY-CGERVEM-EVEVS--E-D--DAN-V-K

---WFK-N-----GEEIF-P------------------------------

---G---P-K--SR--YKIK-V--E--G----------------------

------------------K--KHTLIIEGATKA-DSAEYSAMT-TGG-QS

SAKLSVDL------

>default|Q4TAD4_TETNG/14141500.0|Q4TAD4_TETNG/14141500

----V-TLM----QPLCDLTVC-EGEIAQM-EVKFS--H-G--NVE-G-T

---WLR-N-----GRPIQ-A------------------------------

---S---Q-R--VH--M--V-I--D--K----------------------

------------------Q--IHKLLIEDTNKD-DTGVYSFEV-PAQ-EI

STSAKLLI------

>default|Q7ZZ46_BRARE/14561540.0|Q7ZZ46_BRARE/14561540

----A-EFI----ARPQNQETV-EGEKAEF-VCSVS--K-D--NYE-V-K

---WFK-G-----DKELT-T------------------------------

---D---D-K--YE--I--V-V--D--G----------------------

------------------K--RRALIIKNCELK-DEGAFTANI-GSV-KA

SANLLV--------

>default|A2ASS5_MOUSE/58365920.0|A2ASS5_MOUSE/58365920

----V-EFL----RPLTDLQVK-EKETARF-ECEIS--K-E--NEK-V-Q

---WFK-D-----GAEIK-K------------------------------

---G---K-K--YD--I--I-S--K--G----------------------

------------------A--VRILVINKCLLN-DEAEYSCEV-RTA-RT

SGMLTV--------

>default|TITIN_DROME/1711917204.0|TITIN_DROME/1711917204

----V-DFV----IRLPDITLA-TKTTDAE-FTVQL--S-Qp-DVE-V-T

---WCK-K-----GKPIK-P------------------------------

---N---Q-K--HE--V--F-V--E--G----------------------

------------------T--VRRLVIHDASDE-DAGEISCVA-ENV-TS

STKLCV--------

>default|TITIN_HUMAN/1321213296.0|TITIN_HUMAN/1321213296

----V-EFT----KPLEDQTVE-EGATAVL-ECEVS--R-E--NAK-V-K

---WFK-N-----GTEIL-K------------------------------

---S---K-K--YE--I--V-A--D--G----------------------

------------------R--VRKLVIHDCTPE-DIKTYTCDA-KDF-KT

SCNLNV--------

>default|Q7ZZ46_BRARE/406490.0|Q7ZZ46_BRARE/406490

----M-EFT----VPLKDVTVH-EKKQAKF-ESTIN--K-D--VSK-V-I

---WFK-G-----SDIIT-Q------------------------------

---G---N-K--YE--I--I-D--D--G----------------------

------------------K--KHILIINNCAFE-DEGEYSIEV-LGK-SS

KAKMIV--------

>default|Q4RE91_TETNG/818902.0|Q4RE91_TETNG/818902

----I-SFI----TPLADVYVY-EKDEARF-ELEIS--R-E--PKN-F-R

---WLK-G-----PQELV-N------------------------------

---D---D-K--FE--L--L-V--E--G----------------------

------------------K--KHILLIKSAKYE-DEAKYTFEA-EDK-RT

SGKLFI--------

>default|O76280_DROME/279364.0|O76280_DROME/279364

----N-RFN----KKLKDTEAV-EREKLIL-DIELQ--D-Q--TAP-C-D

---WKF-N-----GEPIV-P------------------------------

---S---G-S--IE--I--K-N-MG--G----------------------

------------------G--KHQLIFSSLDMS-NEGEITFES-GQL-SS

KCKLSI--------

>default|A2ASS5_MOUSE/59256009.0|A2ASS5_MOUSE/59256009

----A-VFT----KNLANLEVS-EGDTIKL-VCEVS--K-P--GAE-V-I

---WYK-G-----DEEII-E------------------------------

---T---G-R--FE--I--L-T--D--G----------------------

------------------R--KRILIIQNAQLE-DAGSYNCRL-PSS-RT

DSKVKV--------

>default|Q4T9D9_TETNG/271358.0|Q4T9D9_TETNG/271358

----V-TIT----KLLDDVHTV-VGERVEF-EVEVS--E-E--GAN-V-K

---WMK-D-----GVELT-R------------------------------

---E---A-Ag-SK--FRFK-K--D--G----------------------

------------------K--KHILIINEATKE-DMGTYQVFT-NGG-ES

KAELEV--------

>default|Q9TXK2_CAEEL/47984887.0|Q9TXK2_CAEEL/47984887

P-----HFI----AKLSDISAR-QGHTVKFSAEVDG--N-P--EPT-V-Q

---WQF-K-----GKPIS-A------------------------------

---S---N-N--VK--I--S-R--D--G----------------------

------------------K--RAILELARVTPD-SAGEYQIVI-RND-KG

AATSQAKLTL----

>default|Q4ZG20_HUMAN/28822966.0|Q4ZG20_HUMAN/28822966

----L-HIT----KTMKNIEVP-ETKTASF-ECEVS--H-F--NVP-S-M

---WLK-N-----GVEIE-M------------------------------

---S---E-K--FK--I--V-V--Q--G----------------------

------------------K--LHQLIIMNTSTE-DSAEYTFVC-GND-QV

SATLTV--------

>default|Q9TXK2_CAEEL/18107.0|Q9TXK2_CAEEL/18107

P-----TFI----RPLADKRAV-VGETIILECQLEG--H-P--DPA-I-K

---WLK-D-----GHNVS-M------------------------------

---C---P-D--YR--I--E-E--D--G----------------------

------------------L--KHRLVIPQVQAA-DSGRFTAQA-SNS-AG

TKQSTCILIC----

>default|Q8N9C0_HUMAN/328414.0|Q8N9C0_HUMAN/328414

----L-KFL----GEMKPVKVT-ERQTAVF-EIRLS--K-K--EPN-F-V

---WKF-N-----GKELK-R------------------------------

---D---D-K--YE--I--T-VSED--G----------------------

------------------L--THTLKIKDARLS-DSGEFSAEA-GNL-VQ

KAQLTI--------

>default|Q4RE90_TETNG/1094.0|Q4RE90_TETNG/1094

----V-EFT----KPLEDQTVE-EEATATL-ECEVS--R-E--NAD-V-L

---WLR-D-----GQEIR-K------------------------------

---T---K-K--YE--M--V-I--D--G----------------------

------------------R--KRALVIHDCTLD-DSRTYTCGA-KDF-KT

SCFLTV--------

>default|Q7YRF6_CANFA/19842068.0|Q7YRF6_CANFA/19842068

----L-IFI----TPLSDVKVF-EKDEAKF-ECEVS--R-E--PKT-F-R

---WLK-G-----TQEIT-G------------------------------

---D---D-K--VE--L--I-K--D--G----------------------

------------------T--KHSLVIKSAAFE-DEGKYIFEA-EDK-HT

SGKLII--------

>default|Q7ZZ46_BRARE/16381722.0|Q7ZZ46_BRARE/16381722

----L-KII----EPLEDCEIE-EKKTITF-TCKVN--R-P--NVT-V-Q

---WMK-A-----GQEIT-L------------------------------

---S---K-R--IL--Y--R-V--D--K----------------------

------------------D--KHTLTIKDCSLA-DEGEYTVVA-GAD-KA

TAELII--------

>default|Q4T444_TETNG/12661350.0|Q4T444_TETNG/12661350

----L-NFL----TSLNDVQVY-EKDEARF-EVELS--R-P--PKS-F-R

---WLK-G-----SQELQ-S------------------------------

---D---E-K--YE--L--I-Q--E--G----------------------

------------------H--TYVLLIRSAIYE-DEAKYMFEA-EDK-RT

ACKLIL--------

>default|OBSCN_HUMAN/20772161.0|OBSCN_HUMAN/20772161

----V-RLV----RGLQAVEAR-EQGTATM-EVQLS--H-A--DVD-G-S

---WTR-D-----GLRFQ-Q------------------------------

---G---P-T--CH--L--A-V--R--G----------------------

------------------P--MHTLTLSGLRPE-DSGLMVFKA-EGV-HT

SARLVV--------

>default|Q7ZZ46_BRARE/11001184.0|Q7ZZ46_BRARE/11001184

----I-GLL----RPLRDVTVT-AGETATF-DCELS--Y-E--DIP-V-E

---WFV-G-----STKME-P------------------------------

---S---D-R--VV--T--R-A--E--G----------------------

------------------R--LHSLTLRDVIMT-EAGEVKLTA-KDF-VT

QAKLTV--------

>default|Q4T444_TETNG/911995.0|Q4T444_TETNG/911995

----M-GFV----DPLKDVSVP-EKKQAKF-ECTVT--K-E--VPK-V-M

---WFK-G-----AEIVT-P------------------------------

---G---P-K--YE--I--I-D--D--G----------------------

------------------L--KHMLVINSCEFD-DEAQYTIEV-LGQ-RS

SSQLRV--------

>default|Q4RE92_TETNG/21102196.0|Q4RE92_TETNG/21102196

----V-TLM----LPLTDLSVC-EGDIAQL-EVRFS--Q-E--NVE-G-T

---WMK-S-----GQAIS-P------------------------------

---S---D-R--VH--I--V-I--D--K----------------------

------------------Q--VHKLLLENVSTE-DAASYSFVV-PAQ-DI

STAGKLSV------

>default|Q4T444_TETNG/10001084.0|Q4T444_TETNG/10001084

----L-NFV----QPLQDQTVK-EGKTAHF-ELELT--H-D--NVP-V-L

---WYR-N-----EVKLH-V------------------------------

---S---R-T--VL--M--H-V--E--G----------------------

------------------K--KHRLEMRTVSLD-DTCQIKAEA-KGI-YS

VAQLTV--------

>default|A2AAJ8_MOUSE/590676.0|A2AAJ8_MOUSE/590676

----V-GIT----KRLKTVEVL-EGESCSFECVLSH--E-S--PSD-PaV

---WTV-G-----GKTVG-G------------------------------

---S---G-H--FH--A--V-R--Q--G----------------------

------------------R--KYTLTVKDAALS-DAGEVVFSV-LGL-TS

KASLII--------

>default|Q7ZZ48_BRARE/21902274.0|Q7ZZ48_BRARE/21902274

----L-RVT----RPLKNIEVP-ETHVATF-ECEVS--H-F--NVP-S-A

---WLK-N-----GVEIE-M------------------------------

---S---E-K--FG--I--V-V--Q--G----------------------

------------------K--IHQLKVMNVSQE-DVSEYTFIC-GND-KV

SANLTV--------

>default|Q4T444_TETNG/22002284.0|Q4T444_TETNG/22002284

----H-RFL----QPIEDIETQ-EKKTISF-VCKVN--R-P--EVM-V-K

---WTK-A-----GQEVA-L------------------------------

---S---K-R--IV--Y--R-A--D--G----------------------

------------------L--KHTLTIKDCVME-DEGEYTAVV-GED-RC

TADLMV--------

>default|Q7YRF6_CANFA/17181802.0|Q7YRF6_CANFA/17181802

----L-KFI----SPLEDQTVK-EGQTATF-VCELS--H-E--KMH-V-V

---WFK-N-----DAKLH-T------------------------------

---S---R-T--VL--I--S-S--E--G----------------------

------------------K--THKLEIREVTLD-DISQIRAQV-KDL-SS

SANLKV--------

>default|Q4RE92_TETNG/24612546.0|Q4RE92_TETNG/24612546

----V-KIK----KTLRNQTVT-ETQDAVF-TLELT--H-A--DVKGS-Q

---WIK-N-----GVELQ-S------------------------------

---S---D-K--YE--I--S-S--D--G----------------------

------------------M--VQSLKIRNCNTQ-DESVYSFKL-GKL-SA

NARLNV--------

>default|A2ASS5_MOUSE/26672752.0|A2ASS5_MOUSE/26672752

----V-KIK----KTLRNLTVT-ETQDAVF-SVELT--H-P--DVKGV-Q

---WIK-N-----GVVLD-S------------------------------

---N---D-K--YE--I--S-V--K--G----------------------

------------------T--LYSLKIKNCAMA-DESVYGFKL-GRL-GA

SARLHV--------

>default|Q98918_CHICK/22602344.0|Q98918_CHICK/22602344

----I-EII----RGLHDITCT-ETQNVSF-EVELS--H-S--GID-V-I

---WHF-K-----GQEIK-A------------------------------

---G---P-K--YK--I--E-A--R--G----------------------

------------------K--IYKLTVVKMMKD-DEGEYVFYA-GGK-KT

SGKLIV--------

>default|Q5BJ14_BRARE/311398.0|Q5BJ14_BRARE/311398

----I-TIT----KLLDDYHVV-VGERVEF-EIEVS--E-E--GAH-V-I

---WLF-E-----DQQLS-R------------------------------

---E---D-Kd-SK--YRFK-K--D--G----------------------

------------------K--RHCLIIQEATLE-DNGMFYVYT-NGG-QS

KGELIV--------

>default|A2AAJ8_MOUSE/412496.0|A2AAJ8_MOUSE/412496

----V-TVI----QPLQDAEAM-EEGRVCF-SCELS--H-K--DED-I-E

---WSL-N-----GTPLY-N------------------------------

---D---S-F--HE--I--S-H--E--G----------------------

------------------C--LHTLVLKSVRQA-DTGTVCATS-PKV-SV

SARLVV--------

>default|A2AAJ9_MOUSE/51035190.0|A2AAJ9_MOUSE/51035190

----V-TIV----RGLVDMEVQ-ADEDVEFTCKVSQ--A-G--ATD-V-Q

---WHL-Q-----GLPLQ-S------------------------------

---N---E-V--TE--V-AVlA--D--G----------------------

------------------C--THVLQLKGVTLE-DAGTVSFHV-GGL-SS

SAQLTV--------

>default|Q4S4W8_TETNG/46133.0|Q4S4W8_TETNG/46133

----V-TIT----KLMDDYHVV-VGERVEF-EVEVS--E-E--GAH-V-M

---WFF-E-----DIELH-K------------------------------

---D---K-Ds-SK--YRFK-K--D--G----------------------

------------------K--KHTLIIQEAMLD-DIGMYHAWT-NGG-HT

KGELEV--------

>default|OBSCN_HUMAN/44324519.0|OBSCN_HUMAN/44324519

----L-EIL----EPLKNAAVR-AGAQACF-TCTLS----E--AVP-Vge

aswYIN-G-----AAVQP-D------------------------------

---D---S-D--WT--V--T-A--D--G----------------------

------------------S--HHALLLRSAQPH-HAGEVTFAC-RDA-VA

SARLTV--------

>default|Q7ZZ46_BRARE/11891273.0|Q7ZZ46_BRARE/11891273

----V-EFS----KPLEDQTVE-EEATAIL-ECELS--R-E--KAD-V-K

---WFR-D-----GQEIR-K------------------------------

---T---K-K--HE--M--V-A--E--G----------------------

------------------R--IRRLIIKDCSLD-DSKTYTCDA-KEF-KT

SAFLNV--------

>default|Q4RU89_TETNG/30363128.0|Q4RU89_TETNG/30363128

----L-FFT----RSLQRVEVE-EGSSASL-RCQLS--K-P--GVS-V-Q

---WKK-N-----RMPLR-A------------------------------

---G---R-K--YE--M--K-Q--D--G----------------------

------------------C--LFELRIKDVLSE-DSGSYTCHA-GNA-ET

SGAVEVKALPTLfi

>default|Q4RE90_TETNG/278362.0|Q4RE90_TETNG/278362

----A-EFI----SKPCSQEVV-EGEKAVF-TCIVS--K-E--TFE-V-K

---WFK-G-----DKELE-D------------------------------

---G---D-K--YQ--M--V-S--D--G----------------------

------------------K--KRTLVIKNCELK-DESGYVVII-GVT-KA

SADLKV--------

>default|OBSCN_HUMAN/21662250.0|OBSCN_HUMAN/21662250

----V-SFS----RPLQDVVTT-EKEKVTL-ECELS--R-P--NVD-V-R

---WLK-D-----GVELR-A------------------------------

---G---K-T--MA--I--A-A--Q--G----------------------

------------------A--CRSLTIYRCEFA-DQGVYVCDA-HDA-QS

SASVKV--------

>default|Q5RGJ3_BRARE/230315.0|Q5RGJ3_BRARE/230315

-E--A-FAK----RLEPAYQVD-KGGKIRL-IVDLA--D-P--TVE-L-K

---WYK-N-----GQEIR-P------------------------------

---T---P-K--FI--F--E-H--K--G----------------------

------------------T--QRIMVINNCQMS-DDAAYSVTA-GEE-KC

TTELFV--------

>default|Q4RE91_TETNG/907991.0|Q4RE91_TETNG/907991

----L-DFI----KPIKDVTVK-ERETAEF-STELS--H-E--KIP-V-V

---WYK-N-----EVRLH-P------------------------------

---S---K-V--VH--I--T-E--D--G----------------------

------------------K--VHMLAFKELTVD-DTSMIKVEA-MGK-SS

EAMLTV--------

>default|Q7YT99_MYTGA/12801364.0|Q7YT99_MYTGA/12801364

----W-DFM----KLLDDVEGI-ERDRATF-ECDVN--D-P--EAE-V-K

---WSR-N-----DKELT-S------------------------------

---G---G-K--YE--I--Q-V--D--G----------------------

------------------F--KRRLVVKNCHSK-DDGPYTCKV-LDK-ET

TANLFV--------

>default|Q4TAD4_TETNG/18531937.0|Q4TAD4_TETNG/18531937

----I-KII----KKPKDVTTL-LGTSAVL-EVVIS--E-D--NIP-V-R

---WMF-N-----RVELK-I------------------------------

---S---D-D--YK--M--V-S--E--K----------------------

------------------K--SHKLIVQNVDKS-KEGEYTAVV-GHL-QT

SACLTV--------

>default|OBSCN_HUMAN/420505.0|OBSCN_HUMAN/420505

G---N-LLR----KLPRKTAVR-VGDTAMF-CVELA--V-P--VGP-V-H

---WLR-N-----QEEVV-A------------------------------

---G---G-R--VA--I--S-A--E--G----------------------

------------------T--RHTLTISQCCLE-DVGQVAFMA-GDC-QT

STQFCV--------

>default|Q4SK55_TETNG/278363.0|Q4SK55_TETNG/278363

G---T-ILR----KLPRKVDVL-EGENAAY-CVEVE--K-E--EMD-I-H

---WYK-D-----GVELR-E------------------------------

---T---H-Q--TI--I--K-S--F--G----------------------

------------------R--THILVFVNTMPQ-DSGIVTFLV-GRS-KT

SSQLRV--------

>default|Q4RH24_TETNG/404488.0|Q4RH24_TETNG/404488

P-----IVR----KLPRKLEVM-EGENAMF-CVEVE--N-D--EEE-I-H

---WFK-D-----GLQLH-E------------------------------

---M---H-Q--VI--L--K-S--F--G----------------------

------------------K--THILVFVNVAHR-DSGVVTFVA-GRS-RT

SSRLKV--------

>default|Q9JHQ1_RAT/147236.0|Q9JHQ1_RAT/147236

P-----TFL----SRPKSLTTF-VGKAAKFLCTVSG--T-P--VIE-T-I

---WQK-D-----GTALS-P------------------------------

---S---P-D--CR--I--T-D--A--D----------------------

------------------N--KHSLELSNLTVQ-DRGVYSCKA-SNK-FG

ADICQAELTI----

>default|Q86GD6_PROCL/15781662.0|Q86GD6_PROCL/15781662

----N-KFK----KKLQDQMTY-ERQPATF-EVELV--D-P--NAP-L-Q

---WFI-N-----GKEVT-E------------------------------

---G---D-N--YE--F--Q-K--K--G----------------------

------------------G--VHRLIIKEAATA-HEGEIKAVC-GNL-ET

ACQLSV--------

>default|Q4SLN7_TETNG/17431827.0|Q4SLN7_TETNG/17431827

----V-RLT----TKLNNVAAM-EGKDAIF-KCAVT--P-A--DAS-V-R

---WFH-K-----SLPIA-A------------------------------

---G---I-K--YK--M--E-H--S--G----------------------

------------------N--CHSLTVTSVSQQ-DAGEVSFEA-EGK-SC

KANLQV--------

>default|Q4RC52_TETNG/24108.0|Q4RC52_TETNG/24108

----M-DFV----VPLKDVTVP-EKKQAKF-ECTIT--K-D--VSK-V-M

---WYR-G-----DDIIT-T------------------------------

---D---Q-K--YD--I--I-D--D--G----------------------

------------------K--KHILVINCCEFD-DEDQYTVAV-LGK-TS

AARLTV--------

>default|A2ASS5_MOUSE/49485031.0|A2ASS5_MOUSE/49485031

----L-DFA----VPLKDVTVP-EKRQARF-ECVLT--R-E---AN-V-I

---WSK-G-----PDIIK-A------------------------------

---S---D-K--FD--I--I-A--D--G----------------------

------------------K--KHILVINDSQFD-DEGVYTAEV-EGK-KT

SAQLFV--------

>default|Q4RE90_TETNG/460544.0|Q4RE90_TETNG/460544

----L-RII----VPLEDIDTQ-EKKTVSF-SCKVN--R-P--NAT-L-K

---WLK-S-----GEEVT-F------------------------------

---N---K-R--LV--Y--R-V--D--V----------------------

------------------D--KHILTIKDCTLA-DEGEYTAIA-GDS-KS

TAELII--------

>default|Q4RE92_TETNG/27222807.0|Q4RE92_TETNG/27222807

---PV-LIT----SMLKDLNAQ-ERDTITF-EVTVN--H-E--GIT-Y-K

---WLK-N-----GVEVK-S------------------------------

---S---D-R--CQ--V--R-S--R--Q----------------------

------------------L--THTLTIRNVHFG-DGGEYQFVA-GSA-AT

SANLFV--------

>default|Q4RE92_TETNG/29022987.0|Q4RE92_TETNG/29022987

--I-R-IAP----PAERDITVL-EKHRATI-EFEVN--E-D--DVE-G-R

---WLK-N-----GAELS-L------------------------------

---E---E-R--FS--Y--V-V--I--R----------------------

------------------K--VHRITISETFRS-DAGEYTFIA-GKN-RS

SVNLRV--------

>default|Q4SAA1_TETNG/429517.0|Q4SAA1_TETNG/429517

----L-KVY----QDLQDMTVM-LGQPIKL-HCEIY--P-G--NVP-G-R

---WYR-N-----GQLIQ-P------------------------------

---S---D-R--IN--I--I-H--R--N----------------------

------------------K--VHRLEIESCSLH-DAGDYTFVP-EGY-SQ

SLSAKVHIId----

>default|Q569K7_HUMAN/252337.0|Q569K7_HUMAN/252337

--A-A-FAK----ILDPAYQVD-KGGRVRF-VVELA--D-P--KLE-V-K

---WYK-N-----GQEIR-P------------------------------

---S---T-K--YI--F--E-H--K--G----------------------

------------------C--QRILFINNCQMT-DDSEYYVTA-GDE-KC

STELFV--------

>default|Q4RE91_TETNG/555636.0|Q4RE91_TETNG/555636

--------K----KAGKIQINV-IXTTARF-ELELS--H-E--NIP-V-T

---WYK-N-----DAKIH-P------------------------------

---S---R-T--VL--T--H-V--D--G----------------------

------------------K--RHVLEIKEVTLD-DTCQIKAEA-KGI-PS

MANLTV--------

>default|Q4RU89_TETNG/378463.0|Q4RU89_TETNG/378463

----I-TIV----QRLKTLSVL-EGETCTFECHLSH--S-V--DDQ-P-C

---WTI-N-----GQVVA-T------------------------------

---D---S-R--LQ--A--I-G--N--G----------------------

------------------C--KYRLTIKEAQLS-DSGDVVFTI-KDL-AC

RTLLFV--------

>default|Q4RU89_TETNG/27762860.0|Q4RU89_TETNG/27762860

P-----IFK----MSLESVEVE-EGSSASL-RCQLS--K-P--GAS-V-Q

---WKK-N-----RMPLR-A------------------------------

---D---G-K--YE--M--K-Q--D--G----------------------

------------------C--LIQLQMKDLQPE-DSGSYSCHA-GSA-VT

SATVAV--------

>default|Q4SLN8_TETNG/325409.0|Q4SLN8_TETNG/325409

----V-LFK----KELENQEVL-EGDKAIL-SCETS--H-P--DCS-V-R

---WLK-G-----STVLT-H------------------------------

---G---D-K--YS--I--E-Q--R--A----------------------

------------------T--AHTLCIHKLDVK-DSGEYTCDT-GDK-RS

SASLTV--------

>default|Q4RE92_TETNG/20212105.0|Q4RE92_TETNG/20212105

----L-ELI----KHMENIEVP-ETYAGEF-EVELS--R-E--DVE-G-S

---WFF-G-----DKELS-P------------------------------

---S---S-K--HI--M--S-S--R--R----------------------

------------------G--RHTLSVKDVRKE-DQGKYTFVC-GDL-RT

SASLKM--------

>default|Q0VD56_BOVIN/447534.0|Q0VD56_BOVIN/447534

---PV-LIT----RPLEDQLVM-VGQRVEF-ECEVS--E-E--GAQ-V-K

---WLK-D-----GVELT-R------------------------------

---E---E-T--FK--YRFK-K--D--G----------------------

------------------Q--KHHLIINEATLE-DAGHYALRT-SGG-QA

LAELIV--------

>default|A2AGQ1_MOUSE/1094.0|A2AGQ1_MOUSE/1094

----S-AFN----KKPRSAEVT-AGSAAVF-EAETE--R-S--GVK-V-R

---WQR-D-----GSDIT-A------------------------------

---N---D-K--YG--L--A-A--E--G----------------------

------------------K--RHTLTVRDASPD-DQGSYAVIA-GSS-KV

KFDLKV--------

>default|A2AAJ9_MOUSE/49235007.0|A2AAJ9_MOUSE/49235007

----V-RFL----RELQAQDVD-EGATARL-RCELS--R-E--AVS-V-E

---WRK-G-----SLQLF-P------------------------------

---C---A-K--YQ--M--V-Q--E--G----------------------

------------------T--TAELLVHGVEQE-DAGEYTCDA-GHT-QS

IARLSV--------

>default|MYPC3_CHICK/1094.0|MYPC3_CHICK/1094

----S-AFT----KKPKTTEVA-AGSTAVF-EAETE--K-T--GIK-V-K

---WQR-A-----GTEIT-D------------------------------

---S---E-K--YA--I--K-A--E--G----------------------

------------------N--KHSLTISNVGKD-DEVTYAVIA-GTS-KV

KFELKV--------

>default|TITIN_HUMAN/1285612940.0|TITIN_HUMAN/1285612940

----L-KFL----TPLKDVTAK-EKESAVF-TVELS--H-D--NIR-V-K

---WFK-N-----DQRLH-T------------------------------

---T---R-S--VS--M--Q-D--E--G----------------------

------------------K--THSITFKDLSID-DTSQIRVEA-MGM-SS

EAKLTV--------

>default|Q4SLN7_TETNG/20992223.0|Q4SLN7_TETNG/20992223

----I-KIV----KKLQDVEVM-EKESATF-VCELS--H-D--EVD-S-Q

---WWK-G-----GTKLK-A------------------------------

---S---D-N--IK--M--R-Q--E--Gitkrtkencmvhlparafrihl

cdfcwipvstsisflvpgR--TYVLLFKSVTPE-DMGEIKFMA-EKA-SS

SAKLKV--------

>default|OBSCN_HUMAN/19882072.0|OBSCN_HUMAN/19882072

----V-LFK----KKLEPQTVE-ERSSVTL-EVELT--R-P--WPE-L-R

---WTR-N-----ATALA-P------------------------------

---G---K-N--VE--I--H-A--E--G----------------------

------------------A--RHRLVLHNVGFA-DRGFFGCET-PDD-KT

QAKLTV--------

>default|OBSCN_HUMAN/40684154.0|OBSCN_HUMAN/40684154

P-----KFK----TRLQSLEQE-TGDIARL-CCQLS--D-AesGAV-V-Q

---WLK-E-----GVELH-A------------------------------

---G---P-K--YE--M--R-S--Q--G----------------------

------------------A--TRELLIHQLEAK-DTGEYACVT-GGQ-KT

AASLRV--------

>default|Q80TU8_MOUSE/694779.0|Q80TU8_MOUSE/694779

--L-Q-MCR----RPPREKTVL-VNRRAVL-EVTVS--R-P--GGH-V-C

---WMR-E-----GVELC-P------------------------------

---G---N-K--YE--M--R-R--H--G----------------------

------------------T--THSLVIHDVRPE-DQGTYSCQA-GQD-SA

DTQLLV--------

>default|Q4RU89_TETNG/31703254.0|Q4RU89_TETNG/31703254

P-----FFI----TELQSVEAE-EGGAASL-CCELS--R-P--AVK-A-Q

---WKK-N-----RIPLR-A------------------------------

---N---S-R--YE--M--K-Q--E--G----------------------

------------------C--LLQLHIQELRPE-DGGSYSCQA-GSA-ET

SARLSV--------

>default|Q95YM2_PROCL/1631616400.0|Q95YM2_PROCL/1631616400

----A-EIV----RKMKDQVVS-KGDRATM-EVELT--K-G--DAV-I-T

---WYK-D-----EVEIR-F------------------------------

---S---D-H--YQ--L--S-I--D--G----------------------

------------------K--VQRLMVYNCQFE-DSGTYRAVV-GKS-EC

SATLKV--------

>default|Q4T444_TETNG/17501834.0|Q4T444_TETNG/17501834

----V-EFT----KPLEDQTVE-EDSTVTL-ECAVS--R-E--NAE-V-R

---WFR-D-----GQELR-K------------------------------

---T---K-K--FD--L--L-S--L--G----------------------

------------------C--KRMLVIHNCSSE-DIKMYTCDT-KDF-KT

SCYLDV--------

>default|A2ASS5_MOUSE/24932577.0|A2ASS5_MOUSE/24932577

----I-KII----RGLRDLTCT-ETQNVVF-EVELS--H-S--GID-V-V

---WNF-K-----GKEIK-P------------------------------

---S---S-K--YK--I--E-A--H--G----------------------

------------------K--IYKLTVLNMMKD-DEGEYAFYA-GEN-TT

SGKLTV--------

>default|A2ASS5_MOUSE/61966280.0|A2ASS5_MOUSE/61966280

----L-RIV----EPLKDIETM-EKKSVTF-WCKVN--R-L--NVT-L-K

---WTK-N-----GEEVA-F------------------------------

---D---N-R--IS--Y--R-I--D--K----------------------

------------------Y--KHSLIIKDCGFP-DEGEYVVTA-GQD-KS

VAELLI--------

>default|A2BEV6_BRARE/777866.0|A2BEV6_BRARE/777866

P-----WFK----KPLKNQSVV-DAEPVRFTVKITG--E-P--KPE-V-T

---WWF-E-----GEPLP-D------------------------------

---S---E-D--YQ--Y--I-E--R--G----------------------

------------------E--TYCLYLPETFPE-DEGEYMCKA-VNS-RG

TAASTCILTI----

>default|Q8T103_BOMMO/47234812.0|Q8T103_BOMMO/47234812

----L-PAL----TPLRDIVVY-EGQPAQFKTQITS--K-I--KPT-I-Q

---WLR-E-----GALIP-E------------------------------

---T---P-D--FQ--M--I-H--E--G----------------------

------------------N--NAVLLIGNTYEE-DTGTFTCRA-TTA-AG

QVETSAKLVV----

>default|Q4SLN8_TETNG/235320.0|Q4SLN8_TETNG/235320

----V-SIL----KFLESCVVS-EGEDVRFECLVSR--E-E--APL-V-Q

---WKL-Q-----DVPLQ-D------------------------------

---N---Q-M--NV--I--E-S--D--G----------------------

------------------R--LHSLTLRGVTEA-DSGTVTFTV-GNH-TS

TASLTV--------

>default|Q7ZZ46_BRARE/13671451.0|Q7ZZ46_BRARE/13671451

----A-RFT----KNLANVEGT-ETDSIKM-VCEVS--K-P--SAE-V-T

---WHK-G-----DQEIP-E------------------------------

---G---G-R--YE--H--V-A--D--G----------------------

------------------K--KRILIIQDLRMD-DAGEYHCKL-PAS-ET

TGTLKI--------

>default|Q4T444_TETNG/14831567.0|Q4T444_TETNG/14831567

----L-YFT----TKLQNYTAV-EKDEVKL-RCELS--K-A--IGD-V-K

---WFK-D-----GKELT-P------------------------------

---S---K-N--IA--I--I-T--D--G----------------------

------------------K--KRILTVRKAEKA-NIGEYTCDC-GSD-KT

TAKLNI--------

>default|MYPC1_RAT/55140.0|MYPC1_RAT/55140

----L-KIT----TPLTDQTVK-LGKEVCL-KCEIS----E--NVP-G-K

---WTK-N-----GLPVQ-E------------------------------

---G---E-R--LK--V--V-H--K--G----------------------

------------------R--IHKLVIANALIE-DEGEYVFTP-DAI-TV

PLVCQIHV------

>default|MYPC2_CHICK/250335.0|MYPC2_CHICK/250335

-E--A-FIR----KLDPAYQVD-KGNKIKL-VVELS--D-P--DLP-L-K

---WYK-N-----GQLLK-P------------------------------

---S---T-K--YV--F--E-N--V--G----------------------

------------------L--KRILTIHKCSLA-DDAAYECRV-NDE-KC

FTEVFV--------

>default|Q4RE92_TETNG/1000010086.0|Q4RE92_TETNG/1000010086

----V-TVL----SDISDQSVF-TGKEAEF-QCEVTINY-P--EIT-L-S

---WYK-G-----TQKLD-S------------------------------

---S---D-K--YD--I--S-I--S--G----------------------

------------------D--RHLLRIRRCQTS-DQGNYRVVC-GPH-IS

SAKLTV--------

>default|Q08476_CHICK/873959.0|Q08476_CHICK/873959

----V-TLI----RDIENQTVL-TDEDAIF-ECEIKINY-P--EIK-L-S

---WYK-G-----TQKLD-S------------------------------

---S---D-K--YK--I--K-I--E--G----------------------

------------------D--RHILKIKNCQLE-DQGNYRIVC-GPH-IA

SARLTV--------

>default|Q4RU89_TETNG/23192403.0|Q4RU89_TETNG/23192403

P-----FFT----KELRDLEVE-EGSSASL-RCELS--K-P--GAS-V-Q

---WKK-N-----RMPLR-A------------------------------

---G---G-K--FE--M--K-Q--D--G----------------------

------------------C--LFQLQMKDLVPE-DSGSYSCHA-DSA-ET

SSLVSV--------

>default|Q4RU89_TETNG/26862771.0|Q4RU89_TETNG/26862771

----T-FFS----SDLQSVEAE-ESGAVTL-CCELS--K-S--GVSGV-Q

---WKK-N-----GVPLR-V------------------------------

---S---R-K--YE--M--K-Q--D--G----------------------

------------------R--LLQLHIKDLTPE-DSGGYSCSA-GNA-KT

SANLVV--------

>default|Q4T444_TETNG/20182102.0|Q4T444_TETNG/20182102

----A-EFI----SRPQNQEVV-EGQAAEF-TCSVS--K-E--TYE-V-K

---WFR-G-----DKELE-N------------------------------

---G---D-K--FV--I--V-S--E--G----------------------

------------------K--RRVLTVKNCELK-DEGGYVAHI-GAI-KA

SADLLV--------

>default|Q90X86_XENLA/377463.0|Q90X86_XENLA/377463

-E--A-FLK----KMDPAYQID-KGQKMKL-VVEVA--N-P--DAE-V-K

---WLK-N-----GQEIRVS------------------------------

---G---S-K--YI--F--E-S--I--G----------------------

------------------N--KRILTINNCSLA-DDAAYTCVI-GEE-KC

FTELFV--------

>default|OBSCN_HUMAN/38903974.0|OBSCN_HUMAN/38903974

P-----VFR----EPLQSLQAE-EGSTATL-QCELS--E-P--TAT-V-V

---WSK-G-----GLQLQ-A------------------------------

---N---G-R--RE--P--R-L--Q--G----------------------

------------------C--TAELVLQDLQRE-DTGEYTCTC-GSQ-AT

SATLTV--------

>default|Q8N9C0_HUMAN/419509.0|Q8N9C0_HUMAN/419509

----I-KFV----SNLKNVRVK-ERSRACL-ECELT--S-K--DVT-L-R

---WKK-D-----GQLLM-H------------------------------

---G---T-K--YS--M--N-H--E--G----------------------

------------------K--QAELIIEDAQLS-DGGEYTVVA-MQD-GD

PTEYYSTAIVTV--

>default|Q28633_RABIT/10831169.0|Q28633_RABIT/10831169

----I-AIL----QGLSDQKVC-EGDIVQL-EVKVS--L-E--NVE-G-V

---WMK-D-----GQEVQ-S------------------------------

---S---D-R--VH--I--V-I--D--K----------------------

------------------H--SHMLLIEDMTKE-DAGNYSFTI-PAL-GL

STSGRVSV------

>default|A2ASS5_MOUSE/18011889.0|A2ASS5_MOUSE/18011889

P-----DIV----LFPEPVRVL-EGETARFRCRVTG--Y-P--QPK-V-N

---WYL-N-----GQLIR-K------------------------------

---S---K-R--FR--V--R-Y--D--G----------------------

------------------I--HYLDIV-DCKSY-DTGEVKVTA-ENP-EG

VTEHKVKLEI----

>default|Q4SM88_TETNG/19212006.0|Q4SM88_TETNG/19212006

----L-VVV----KHLEDLEVT-EPDAASFQCQVSV--A-I--NKP-P-V

---WTL-N-----GENLQ-P------------------------------

---G---P-W--VH--L--E-N--H--G----------------------

------------------T--TYKLRLKSTSTD-MSGVVKFTL-GKA-RS

SASLTV--------

>default|Q4RE91_TETNG/417509.0|Q4RE91_TETNG/417509

LKF-IKKLQ----DIVLKEAES-IGSSAVF-ECEVS--P-S--TAV-T-S

---WMK-D-----GGNLR-E------------------------------

---S---P-K--HK--F--T-S--D--G----------------------

------------------K--DRKLNIIDVQLS-DTGEYTCVA-KNA-GK

EISCMAKLIV----

>default|Q61SF4_CAEBR/14041494.0|Q61SF4_CAEBR/14041494

P-----EFTg---GGIKDLRLK-VGETIKYEVPISG--E-P--LPD-C-T

---WIV-N-----GKPLK-A------------------------------

---V---G-R--VK--M--S-S--E--R----------------------

------------------G--KHVMKIENAVRG-DSGQYTITL-KNS-SG

TCDSTATVTV----

>default|Q4RZI3_TETNG/21103.0|Q4RZI3_TETNG/21103

-------IV----KPIKDQHVK-PKATATF-KCELF--K-D--TPN-W-K

---WLK-G-----ENEIT-P------------------------------

---S---D-K--VE--I--K-K--D--G----------------------

------------------K--NLTLTIKNCQPD-DIAEYSLEV-EGR-TY

TAKLTL--------

>default|OBSCN_HUMAN/28272911.0|OBSCN_HUMAN/28272911

----A-AII----KPLEDQWVA-PGEDVEL-RCELS--R-A--GTP-V-H

---WLK-D-----RKAIR-K------------------------------

---S---Q-K--YD--V--V-C--E--G----------------------

------------------T--MAMLVIRGASLK-DAGEYTCEV-EAS-KS

TASLHV--------

>default|Q1JPV5_BRARE/406492.0|Q1JPV5_BRARE/406492

----L-EVL----QSIADLTVK-AAEQAVF-KCEVS--D-E--KVT-G-K

---WFK-D-----GVEVV-A------------------------------

---S---D-R--IK--M--S-H--I--G----------------------

------------------R--THKLVISDVKPE-DAGDYTFVP-DGY-AL

SLSAKLNF------

>default|Q95YM2_PROCL/35543645.0|Q95YM2_PROCL/35543645

PQ--F-VTQ----PQDSTIQEN-GLAHFECRLLPVN--D-P--NLR-V-E

---WYH-N-----GRPLS-A------------------------------

---G---S-R--IK--T--I-N--D--F----------------------

------------------G--YVILEMANCYGK-DSGTYTCRA-VNK-HG

EASVECKLVV----

>default|Q7YRF6_CANFA/98187.0|Q7YRF6_CANFA/98187

P-----VFI----KEVSNAEIS-IGDVAKLSVTVTG--I-P--KPK-I-Q

---WFF-K-----GVMLT-P------------------------------

---S---A-D--YK--F--V-F--D--G----------------------

------------------N--DHSLIILFTKFE-DEGEYTCVA-SNE-YG

QAVCSASLKI----

>default|MYPC2_CHICK/340426.0|MYPC2_CHICK/340426

----V-TVV----RGLEDQQVV-VGDRVVL-EAEVS--E-E--GAQ-V-M

---WLK-D-----GVDVT-R------------------------------

---D---D-A--FK--YRFK-K--D--G----------------------

------------------K--KHFLIINEAELS-DSAHYKIMT-NGG-ES

EAELSV--------

>default|A1L4G9_HUMAN/346434.0|A1L4G9_HUMAN/346434

----V-LIV----TPLEDQQVF-VGDRVEM-AVEVS--E-E--GAQ-V-M

---WMK-D-----GVELT-R------------------------------

---E---D-S--FKArYRFK-K--D--G----------------------

------------------K--RHILIFSDVVQE-DRGRYQVIT-NGG-QC

EAELIV--------

>default|Q7YT99_MYTGA/35423631.0|Q7YT99_MYTGA/35423631

P-----EFS----SGLRKCHAQ-QGKSAKFECEVSG--K-P--SPD-I-R

---WFK-G-----TREIY-S------------------------------

---S---P-K--FE--I--Y-N--E--G----------------------

------------------D--KQVLIVHDVFGE-DQDEYSCRA-SNH-GG

SRTSRANLEI----

>default|Q4RU89_TETNG/22302314.0|Q4RU89_TETNG/22302314

P-----FFQ----DGLQSVEVE-EGGSAFL-CCELS--K-P--GVP-V-D

---WKK-D-----RLPLR-S------------------------------

---S---R-K--HE--I--K-Q--D--G----------------------

------------------C--FLQLHIRDLKPE-DSGSYLCQA-GSA-ET

TAVVTV--------

>default|Q7QJK9_ANOGA/9261010.0|Q7QJK9_ANOGA/9261010

P-----EII----SAPRDVTVA-RGEDAIF-ELELT--K-G--DAL-V-R

---WYR-N-----EEELS-F------------------------------

---N---G-H--VQ--L--T-I--D--G----------------------

------------------K--RQTLKINKTVDA-DGGEYTCVV-GEQ-RA

KARLTV--------

>default|Q569K7_HUMAN/433518.0|Q569K7_HUMAN/433518

----L-KIL----TPLTDQTVN-LGKEICL-KCEIS----E--NIP-G-K

---WTK-N-----GLPVQ-E------------------------------

---S---D-R--LK--V--V-H--K--G----------------------

------------------R--IHKLVIANALTE-DEGDYVFAP-DAY-NV

TLPAKVHV------

>default|Q61S53_CAEBR/17901882.0|Q61S53_CAEBR/17901882

PKF-V-TQL----HSLDGVVEG-QPSHFEAQFIPFS--D-P--RTS-V-Q

---WYL-N-----GQPLS-A------------------------------

---S---S-R--RI--L--R-N--D--F----------------------

------------------G--LVSLDLQYTLGE-DAGEYSVVV-KNS-EG

EDRTNGSLSC----

>default|Q59E65_DROME/25262615.0|Q59E65_DROME/25262615

P-----VFT----QKISDQQQV-FGNNAKIPVTVSG--V-P--YPD-L-E

---WYF-Q-----DKPIP-K------------------------------

---S---E-K--YS--I--K-N--D--G----------------------

------------------D--HHMLIVNNCEKG-DQGVYKCIA-SNR-EG

KDITQGRLDI----

>default|Q2LYU8_DROPS/43834472.0|Q2LYU8_DROPS/43834472

P-----VFT----KKIQPCRVF-ENEQAKFEVEFDG--E-P--NPT-V-K

---WYR-E-----SFPIQ-N------------------------------

---S---P-D--LQ--I--H-T--F--S----------------------

------------------G--KSILIIRQVFVE-DSAVFSCVA-ENR-GG

TAKCSANLVV----

>default|Q90X86_XENLA/892.0|Q90X86_XENLA/892

----S-VFT----KKPKSEEVS-VGGTVTF-VAETD--K-P--GLK-V-K

---WQR-D-----NVDIA-A------------------------------

---N---E-R--FA--I--K-S--E--G----------------------

------------------K--QHSLTISNAIAE-DEVVYAIIV-GTS-KV

KFELKV--------

>default|Q17AF2_AEDAE/35633647.0|Q17AF2_AEDAE/35633647

P-----EII----TPLKDVTVA-RGENAVF-DIELT--K-G--DAL-V-R

---WYR-D-----DTELS-F------------------------------

---N---G-H--YQ--L--T-I--D--G----------------------

------------------K--RQTLKIIKTVDE-DAGVYKCVV-GDQ-LS

TAKLTV--------

>default|Q98918_CHICK/25222606.0|Q98918_CHICK/25222606

----V-KII----KKPKDVTAL-ENAVVSF-ELSVS--H-D--TVP-V-R

---WFH-K-----NVELK-Q------------------------------

---S---D-K--YK--M--I-S--Q--R----------------------

------------------K--VHKLMLHNISPA-DAGEYTAFV-GQL-EC

KAKLFV--------

>default|TITIN_HUMAN/1204212134.0|TITIN_HUMAN/1204212134

LKF-VKEIK----DIILTESEF-VGSSAIF-ECLVS--P-S--TAI-T-T

---WMK-D-----GSNIR-E------------------------------

---S---P-K--HR--F--I-A--D--G----------------------

------------------K--DRKLHIIDVQLS-DAGEYTCVL-RLG-NK

EKTSTAKLVV----

>default|Q290B4_DROPS/287.0|Q290B4_DROPS/287

---------------LKSVQVD-IGETAHF-EIQFK--D-Q--PGL-V-T

---WLK-D-----NKPLE-D------------------------------

---R---LAD--RV--I--Q-T--SaPM----------------------

------------------N--SYRLDIKNCSES-DAGTYTIRA-QSA-SE

TTTLSAQLAV----

>default|Q4SI34_TETNG/1094.0|Q4SI34_TETNG/1094

----S-AFT----KKPKTLEAA-AGSSVVF-EAETE--K-P--DAK-V-K

---WQC-N-----AKDIA-N------------------------------

---G---N-K--YT--I--A-A--D--G----------------------

------------------S--KHSLSIKAVTHQ-DANIYAVIS-GGS-KV

KFELKV--------

>default|A2AAJ9_MOUSE/23322416.0|A2AAJ9_MOUSE/23322416

----V-KFT----SGLSAMVAE-EGQEATF-QCVVS--P-S--DAG-V-T

---WYK-D-----GMQLQ-P------------------------------

---S---E-K--FV--M--V-E--S--G----------------------

------------------A--SRSLTILGLTLE-DAGQVTVEA-EGA-SS

SAALRV--------

>default|Q98918_CHICK/21722257.0|Q98918_CHICK/21722257

----V-EII----VPLKDVHVV-EGTKAILECKVSA--P-D--VTS-S-K

---WYL-N-----DHQIK-P------------------------------

---D---E-R--VQ--A--V-C--K--G----------------------

------------------T--KQRLVITRTHAS-DEGHYKLMV-GKV-ET

SCNVTV--------

>default|Q2LYU8_DROPS/22102302.0|Q2LYU8_DROPS/22102302

PHF-T-AEL----RGSTEIYEG-QTAHFEAQVAPVH--D-P--NLR-I-E

---FYH-N-----GKPLP-S------------------------------

---A---A-R--FH--I--T-F--D--F----------------------

------------------G--YVSLDITHAVAE-DAGEYSVRA-VNA-MG

QAVSATNLRV----

>default|TITIN_HUMAN/3272232812.0|TITIN_HUMAN/3272232812

P-----MFK----RLLANAECQ-EGQSVCFEIRVSG--I-P--PPT-L-K

---WEK-D-----GQPLS-L------------------------------

---G---P-N--IE--I--I-H--E--G----------------------

------------------LD-YYALHIRDTLPE-DTGYYRVTA-TNT-AG

STSCQAHLQV----

>default|Q7ZZ46_BRARE/495579.0|Q7ZZ46_BRARE/495579

----L-NFT----STLKDLTVK-EGKTAVF-ELELS--H-E--NVP-V-T

---WYK-N-----EIKVH-P------------------------------

---S---R-T--VF--T--S-V--V--G----------------------

------------------K--KHTLEIKEVSLD-DTCQIKAEA-KGI-FT

TANLLV--------

>default|A2AAJ9_MOUSE/34373523.0|A2AAJ9_MOUSE/34373523

----V-RVI----EDLEDTAVQ-EGSSAKFCCRIAP--A-D--YGP-V-H

---WFL-D-----KTPLH-S------------------------------

---N---E-L--NE--I-TV-Q--P--G----------------------

------------------G--YHVLTLRQLALK-DSGTVYFEA-GDQ-RT

SAALRV--------

>default|TITIN_HUMAN/1294513029.0|TITIN_HUMAN/1294513029

P-----YFT----GKLQDYTGV-EKDEVIL-QCEIS--K-A--DAP-V-K

---WFK-D-----GKEIK-P------------------------------

---S---K-N--AV--I--K-A--D--G----------------------

------------------K--KRMLILKKALKS-DIGQYTCDC-GTD-KT

SGKLDI--------

>default|Q4TAD4_TETNG/22982387.0|Q4TAD4_TETNG/22982387

P-----QII----RHLEPKVAS-VGRAVRFSLQVGG--L-P--KPQ-V-C

---WSR-D-----SEPLI-A------------------------------

---S---D-T--CK--F--F-H--D--D----------------------

------------------E--EHTLMLLNVLAE-DAGTYSCEA-KNE-YG

EATSSAPLTV----

>default|TITIN_DROME/1685116935.0|TITIN_DROME/1685116935

--V-T-FTK----KLKKTITIE-EVQSLTL-ECETS--H----VVT-T-K

---WFF-N-----GKELS-G------------------------------

---M---D-H--RV--V--V-E--D--G----------------------

------------------K--THKLVIRNTNLR-DSGTYTCKV-KKQ-ET

QSTVEV--------

>default|Q4SM88_TETNG/16541738.0|Q4SM88_TETNG/16541738

----V-SIL----KTLSDIRVE-EDFPATL-ECEFS--R-Q--IIE-V-R

---WFK-N-----GAELR-P------------------------------

---G---K-N--CR--I--Y-S--T--G----------------------

------------------R--KRFCQIMQCSRA-DSGTYTCDT-GEM-NT

SCTLEV--------

>default|Q95YM2_PROCL/44224511.0|Q95YM2_PROCL/44224511

P-----FFT----KKIQPCRAF-EREPARFQVEFDG--D-P--NPT-I-Q

---WYR-E-----DFLIT-S------------------------------

---S---P-D--FQ--I--H-T--F--G----------------------

------------------D--KSILVIREVFLE-DSGLFACVA-ENR-GG

RAKCSANLVV----

>default|Q0VD56_BOVIN/357443.0|Q0VD56_BOVIN/357443

--T-A-FQK----KLQPAYQVS-KGHKIRL-MVELA--D-P--DAE-V-K

---WLK-N-----GQEIQMS------------------------------

---G---S-K--YI--F--E-S--I--G----------------------

------------------A--KRTLTISQCSLA-DDAAYQCVV-GGE-KC

STELFV--------

>default|Q4SLN7_TETNG/18321916.0|Q4SLN7_TETNG/18321916

----V-MFK----EKLENLTVE-EQSEAKL-EVELS--K-P--SEE-V-R

---WMK-N-----SVVIQ-P------------------------------

---T---G-N--IK--I--R-T--D--G----------------------

------------------A--KQALVFMSVSRA-DRGVYSCET-LDD-KT

QAKLSV--------

>default|Q4RU89_TETNG/21412225.0|Q4RU89_TETNG/21412225

----V-LFE----EKPRDLVVM-EGETAVL-SCVTT--D-F--T-SPV-T

---WKC-N-----HVPLR-N------------------------------

---G---N-K--YE--M--H-K--E--G----------------------

------------------K--VNLLHIHDADPQ-DTGIYSCDT-GDV-QS

SARLTV--------

>default|OBSCN_HUMAN/38023885.0|OBSCN_HUMAN/38023885

----A-RFI----EDVKNQEAR-EGATAVL-QCELS--K----AAP-V-E

---WRK-G-----SETLR-G------------------------------

---G---D-R--YS--L--R-Q--D--G----------------------

------------------T--RCELQIHGLSVA-DTGEYSCVC-GQE-RT

SATLTV--------

>default|Q4RE91_TETNG/9961080.0|Q4RE91_TETNG/9961080

----V-YFT----VKLQNYTAV-EKDEAIL-SCELS--K-A--TAD-V-K

---WFK-D-----GEEVF-P------------------------------

---S---K-N--IL--I--K-S--D--G----------------------

------------------K--KRMLVIKKAAKS-SAGTYTCSC-ATD-KT

TSDLNI--------

>default|TITIN_HUMAN/1375013834.0|TITIN_HUMAN/1375013834

----T-EFV----EHLEDQTVT-EFDDAVF-SCQLS--R-E--KAN-V-K

---WYR-N-----GREIK-E------------------------------

---G---K-K--YK--F--E-K--D--G----------------------

------------------S--IHRLIIKDCRLD-DECEYACGV-EDR-KS

RARLFV--------

>default|Q98918_CHICK/19942078.0|Q98918_CHICK/19942078

----V-EFI----KELEDVEVP-ESFTGEL-ECEVS--P-E--DIE-G-K

---WYH-G-----DVELS-S------------------------------

---N---H-K--YV--L--A-S--R--R----------------------

------------------G--RRILTIKDVNKD-DQGEYSFVV-DGK-RT

HCKLKM--------

>default|Q4RE92_TETNG/22872371.0|Q4RE92_TETNG/22872371

----V-HIV----KHMEDRVCS-ESQKVTF-RVEVS--H-P--GID-P-V

---WTV-R-----NQLLK-P------------------------------

---G---A-K--YK--M--E-S--K--D----------------------

------------------K--VHSLTILDTMKD-EEGQYMFHA-GEQ-TS

SAMLTV--------

>default|Q17AE9_AEDAE/12721363.0|Q17AE9_AEDAE/12721363

PQ--F-VRP----LHNIETFEG-TNIHLECRLQPVG--D-P--SMR-V-D

---WFV-N-----GQPVK-I------------------------------

---G---H-R--FR--P--A-Y--E--F----------------------

------------------D--YVALDLLGVYPV-DSGVYTCQA-RNS-LG

EAVTSCSVRI----

>default|Q17AF2_AEDAE/36523736.0|Q17AF2_AEDAE/36523736

----V-DFV----KRLPDVTIA-TKQTDAV-FVVEL--S-E--SAE-V-T

---WYK-N-----SKKIK-A------------------------------

---S---S-K--YE--L--I-A--D--K----------------------

------------------K--VRKLIVKNVTED-DEDEYTCTI-ANV-KT

SSKLKV--------

>default|Q17HV9_AEDAE/349433.0|Q17HV9_AEDAE/349433

----Y-KFT----KVLKSQQLF-VKDTVTL-ACELD--H-V--LGT-V-E

---WFK-G-----DEKIV-E------------------------------

---S---D-K--VQ--I--I-K--D--G----------------------

------------------R--KHKLVLKDVAKA-DSGMYRCVS-NAD-KT

EAEIFV--------

>default|Q4RE92_TETNG/80578146.0|Q4RE92_TETNG/80578146

P-----CIL----EKPESMNVL-PGSKVHFNVLLSG--T-P--PLT-I-K

---WFK-N-----QKEIL-S------------------------------

---S---A-D--CS--V--I-K--D--N----------------------

------------------T--STSLALFFAKSS-DSGEYRCEI-QND-VG

STSCQATLFV----

>default|Q86VF2_HUMAN/73157.0|Q86VF2_HUMAN/73157

----G-HFS----QGLADMEVQ-PGEAATL-SCTLT--S-D--LGP-G-T

---WFK-D-----GVKLT-T------------------------------

---Q---D-G--VI--F--K-Q--D--G----------------------

------------------L--VHSLFITHVQGT-QAGRYTFVA-GDQ-QS

EATLTV--------

>default|Q4T3W5_TETNG/34133501.0|Q4T3W5_TETNG/34133501

P-----MFK----RLLANLECK-EGQDVRFEIRVSG--H-----PT-L-R

---WEK-D-----GAPLA-F------------------------------

---G---P-S--IE--V--V-H--E--G----------------------

------------------LD-YFILHVRDVLPE-DSGVYRVTA-TNS-AG

SASCQATLKV----

>default|UNC89_CAEEL/21712262.0|UNC89_CAEEL/21712262

---PK-FKS----QLTDKVADEGEPLRWNL-ELDGP--S-P--GTE-V-S

---WLL-N-----GQPLT-K------------------------------

---S---D-T--VQ--V--V-D-HG--D----------------------

------------------G--TYHVTIAEAKPE-MSGTLTAKA-KNA-AG

ECETSAKVTV----

>default|Q4T444_TETNG/65116601.0|Q4T444_TETNG/65116601

--I-E-VDV----KLIEGVIAK-AGTTIVLPAKMTG--I-P--IPT-A-K

---WMT-D-----GKDIT-S------------------------------

---E---G-R--CH--I--E-T--A--G----------------------

------------------S--STILSISECQRG-DTGEYVLIV-TNP-AG

SKTVALHVTV----

>default|Q4RU89_TETNG/16671751.0|Q4RU89_TETNG/16671751

----V-TFK----QEMENLVVK-EGDSGEF-CCELS--K-P--GAP-V-D

---WRK-G-----RVILK-P------------------------------

---G---Y-K--YE--M--K-L--E--G----------------------

------------------R--LNKLIINNIEES-DAGKYTCKS-SDS-QS

TAELTV--------

>default|Q7KQP5_DROME/15391623.0|Q7KQP5_DROME/15391623

----Y-KFV----KVLKSQQCI-EKDTVTL-ACEID--D-A--MGE-V-Q

---WLR-N-----GEEIK-P------------------------------

---D---K-R--IQ--I--V-K--D--G----------------------

------------------R--KRKLVIKDCKVT-DAGQFKCTT-NAD-TT

ESEIII--------

>default|TITIN_HUMAN/1259012673.0|TITIN_HUMAN/1259012673

P-----YFT----VKLHDKTAV-EKDEITL-KCEVS--K----DVP-V-K

---WFK-D-----GEEIV-P------------------------------

---S---P-K--YS--I--K-A--D--G----------------------

------------------L--RRILKIKKADLK-DKGEYVCDC-GTD-KT

KANVTV--------

>default|Q4TAD4_TETNG/20262111.0|Q4TAD4_TETNG/20262111

---PI-IIT----TPLQDLHAQ-EKDTVTF-EVGVN--Y-D--QIS-Y-R

---WLK-N-----GVEIR-P------------------------------

---T---D-R--CQ--A--R-C--K--Q----------------------

------------------L--THTLSIRNVHFG-DSATYTFMA-GSA-TT

QAQLSV--------

>default|Q4SI34_TETNG/428515.0|Q4SI34_TETNG/428515

---PV-LIV----KNLEDQMVM-KGDRVEL-ECEVS--E-E--GAN-V-K

---WEK-D-----GVELT-R------------------------------

---D---E-S--FK--YRFK-K--D--G----------------------

------------------C--KHILIINDATKE-DCGHYKVKT-NGG-ES

MAELLV--------

>default|MYPC3_CHICK/544630.0|MYPC3_CHICK/544630

----L-EVY----QSIADLTVK-ARDQAVF-KCEVS--D-E--NVK-G-I

---WLK-N-----GKEVV-P------------------------------

---D---E-R--IK--I--S-H--I--G----------------------

------------------R--IHKLTIEDVTPG-DEADYSFIP-QGF-AY

NLSAKLQF------

>default|Q8T103_BOMMO/31503242.0|Q8T103_BOMMO/31503242

PRF-T-THI----QNIEGLKDG-QSAHFECTVVPVD--D-P--DMK-I-E

---WYH-N-----GQPMR-H------------------------------

---S---T-R--IK--T--V-S--D--F----------------------

------------------G--FVVLDIAYTQNH-DSGEYVCRA-YNK-YG

EDFTRATINC----

>default|Q4S3Q6_TETNG/633722.0|Q4S3Q6_TETNG/633722

P-----QFI----QKLRSREIA-EGSKVQLDCIVRG--L-P--PPE-V-R

---WFC-E-----GKELE-N------------------------------

---S---P-D--IQ--I--L-N--N--G----------------------

------------------E--LHSLIISEAFEE-DTGRYSCFA-SNV-CG

TDSTSAEIYV----

>default|Q90YM0_BRARE/20110.0|Q90YM0_BRARE/20110

----L-QVS----ISLKKVELS-VGESKFF-TCTVI--G-E--PVK-L-E

---WFNpQ-----GERMV-A------------------------------

---S---P-R--VA--L--H-N--E--G----------------------

------------------I--RSRLTLYNAKIE-DAGIYRCQA-TDA-QG

ETQEATVVLei---

>default|Q9H8B3_HUMAN/348434.0|Q9H8B3_HUMAN/348434

--L-L-FLR----RLQDVRAEEGQDVCLEM-ETGRV--G-A--AGA-V-R

---WVR-G-----GQPLP-H------------------------------

---D---S-R--LS--M--A-Q--D--G----------------------

------------------H--IHRLFIHGVILA-DQGTYGCES-HHD-RT

LARLSV--------

>default|Q8NHN3_HUMAN/11581241.0|Q8NHN3_HUMAN/11581241

----A-RFT----QDLKTKEAS-EGATATL-QCELS--K----VAP-V-E

---WKK-G-----PETLR-D------------------------------

---G---G-R--YS--L--K-Q--D--G----------------------

------------------T--RCELQIHDLSVA-DAGEYSCMC-GQE-RT

SATLTV--------

>default|Q4SLN8_TETNG/43127.0|Q4SLN8_TETNG/43127

----V-LFT----KELESQTVD-EGDDVRL-HCELS--K-P--AVP-L-E

---WKK-G-----ELGLC-P------------------------------

---C---T-K--YD--I--K-Q--A--G----------------------

------------------T--RATLVIHDVDPE-DSGSYTCDT-GDC-QS

TAQLIV--------

>default|Q08476_CHICK/9621046.0|Q08476_CHICK/9621046

P-----AVE----RHLHDTTFK-EGNTCTL-SCQFS--I-P--NAK-S-Q

---WYR-N-----GRPIK-I------------------------------

---G---G-R--YS--T--Q-V--S--D----------------------

------------------K--VHKLIIKDVRTE-DQGQYTCKL-DNL-ET

TADLTI--------

>default|Q17AE9_AEDAE/22032295.0|Q17AE9_AEDAE/22032295

PRF-V-TEL----RGTTEIHEG-QTAHFEAQVEPIH--D-P--NLR-I-E

---FYH-N-----GKPLQ-S------------------------------

---A---A-R--FH--I--T-F--D--F----------------------

------------------G--YVALDIQHVVAE-DAGEYTVKA-INA-KG

QCKSSINMRV----

>default|Q4RU89_TETNG/14891573.0|Q4RU89_TETNG/14891573

----I-KFL----QELKNVQVQ-EGNEVKL-RCEIS--K-A--GAS-V-E

---WKK-G-----EKVLS-D------------------------------

---G---E-K--YQ--M--K-Q--S--G----------------------

------------------S--ILELVIRKCQPE-DSGTYSCVC-AEV-RS

TATIII--------

>default|TITIN_DROME/68416930.0|TITIN_DROME/68416930

P-----RFI----QPLEPKYFG-EHEVAIIEAIVES--E-P--LSS-F-Q

---WFV-H-----NEPIK-S------------------------------

---S---N-E--VR--I--V-S--Q--A----------------------

------------------N--KSTLLIENFQSK-FVGPFTCRA-ENV-GG

SVTSTATVNL----

>default|Q5UE88_CAEEL/1099.0|Q5UE88_CAEEL/1099

P-----KFV----EILVDKTET-VDNTVVFEVRVEG--E-P--KPT-V-T

---WYL-K-----GEELK-Q------------------------------

---S---D-R--VE--I--R-E--F--D----------------------

------------------G--SIKISIKNIKIE-DAGEIRAVA-TNS-EG

SDETKAKLTV----

>default|Q9VZT8_DROME/122211.0|Q9VZT8_DROME/122211

P-----RFI----DELVDTNAV-EDERIEFRVRILG--E-P--PPE-I-N

---WFK-D-----GYEIF-S------------------------------

---S---R-R--TK--I--V-N--D--N----------------------

------------------E--ASVLVIHQVALT-DEGEIKCTA-TNR-AG

HVITKARLMV----

>default|Q4RU89_TETNG/24082492.0|Q4RU89_TETNG/24082492

P-----FFI----DCLQSVTAH-EGDTLSL-KCELS--K-P--GVA-V-Q

---WKR-N-----KVVLR-S------------------------------

---N---R-K--YE--L--K-Q--E--G----------------------

------------------C--LLQLSIRGLTPE-DHGSYSCHA-GSA-ET

AALVTV--------

>default|A2AT70_MOUSE/27372826.0|A2AT70_MOUSE/27372826

----I-QFT----KRIQNIVVS-EHQSATF-ECEVS--F-D--DAI-V-T

---WYK-G-----PTELT-E------------------------------

---S---Q-K--YN--F--R-N--D--G----------------------

------------------R--CHYMTIHNVTPD-DEGVYSVIA-RLEpRG

EARSTAELYL----

>default|Q2LYU8_DROPS/16791770.0|Q2LYU8_DROPS/16791770

PV--F-TMP----VRDVRVAEN-QAVHFEARLIPVG--D-P--NLK-V-E

---WLR-N-----GQPIE-A------------------------------

---S---N-R--TT--T--M-H--D--F----------------------

------------------G--YVALNMKYVNPE-DSGSYSCRA-INE-LG

QAVTSASLIV----

>default|Q8T103_BOMMO/770862.0|Q8T103_BOMMO/770862

PQF-I-RPL----QDLGELQEG-RNAHFEAQLTPVS--D-P--TMR-V-E

---WYK-D-----GRPIT-A------------------------------

---S---S-R--IT--S--I-F--N--F----------------------

------------------G--YVSLNIMHLRAE-DAGSYTVRA-VNK-LG

EAISTASIRV----

>default|Q7PT04_ANOGA/26762767.0|Q7PT04_ANOGA/26762767

PV--F-VDH----LNNVEIKEG-ENAHFECRVEPSK--D-P--TMQ-I-E

---WFV-N-----GKPLP-T------------------------------

---G---A-R--FK--T--T-Y--D--F----------------------

------------------G--FVSLDLTGAYAE-DSGIYTCKA-TNS-KG

SASTSGSLRC----

>default|Q24463_DROME/15041593.0|Q24463_DROME/15041593

P-----KII----TPLNEVRIK-CGLIFHTDIHFIG--E-P--APE-A-T

---WTL-N-----SNPLL-S------------------------------

---N---D-R--ST--I--T-S--I--G----------------------

------------------H--HSVVHTVNCQRS-DSGIYHLLL-RNS-SG

IDEGSFELVV----

>default|Q4S5Q4_TETNG/389.0|Q4S5Q4_TETNG/389

--------S----VFTQAVKVK-AGQNAIFKMQFVG--Q-D--PIK-V-Q

---WYR-E-----GEELL-N------------------------------

---D---T-N--VR--V--E-K--C--T----------------------

------------------S--QSRLLLSRCQRK-DTGEIKIKL-KNE-HG

VTEAISQLTV----

>default|UNC89_CAEEL/40184107.0|UNC89_CAEEL/40184107

P-----GFF----KELSAIQVK-ETETAKF-ECKVS--G-T--KPD-V-K

---WFK-D-----GTPLK-E------------------------------

---D---K-R--VH--F--E-S--T--D----------------------

------------------D--GTQRLVIEDSKT-DDQGNYRIEVSND-AG

VANSKVPLTV----

>default|Q801W8_BRARE/1773317823.0|Q801W8_BRARE/1773317823

P-----MFK----RLLANIDCV-EGQSVRFDLRVSG--T-P--APT-L-K

---WEK-N-----GKPLE-F------------------------------

---R---P-Q--VE--V--V-Q--E--D----------------------

------------------VD-YFILHIRETLIE-DSGTYRVTA-TNT-AG

SASCQATLKV----

>default|MYLK_CHICK/735824.0|MYLK_CHICK/735824

P-----WFI----SKPRSVTAA-AGQNVLISCAIAG--D-P--FPT-V-H

---WFK-D-----GQEIT-P------------------------------

---G---T-G--CE--I--L-Q--N--E----------------------

------------------D--IFTLILRNVQSR-HAGQYEIQL-RNQ-VG

ECSCQVSLML----

>default|TITIN_DROME/47024792.0|TITIN_DROME/47024792

P-----PTI----TALKDVSVT-EGMPAQFKTTVTG--K-V--KATSV-Q

---WFR-E-----GQLIP-E------------------------------

---T---P-D--FQ--M--I-F--D--G----------------------

------------------N--SAVLLIGTTYEE-DSGIFTVRV-TSS-TG

QVESSAKLTV----

>default|Q5RHD8_BRARE/224313.0|Q5RHD8_BRARE/224313

P-----RFT----QKLKSQEVA-EGSPIRLECRVTG--N-P--LPL-V-R

---WFC-E-----GRELH-H------------------------------

---C---P-D--IQ--I--C-R--D--G----------------------

------------------D--LHTLVITEAFED-DTGRYTCVA-SNA-LG

ADNTSAEVYI----

>default|TITIN_HUMAN/3261732709.0|TITIN_HUMAN/3261732709

P-----EFT----LPLYNKTAY-VGENVRFGVTITV--H-P--EPH-V-T

---WYK-S-----GQKIK-P------------------------------

---GdndK-K--YT--F--E-S--D--K----------------------

------------------G--LYQLTINSVTTD-DDAEYTVVA-RNK-YG

EDSCKAKLTV----

>default|Q7ZZ46_BRARE/18161900.0|Q7ZZ46_BRARE/18161900

----V-EIV----RPPTDVFEP-PGSDIVF-EAELN--K-D--RVE-V-K

---WMR-N-----NMIIV-Q------------------------------

---G---D-K--YQ--M--I-S--E--G----------------------

------------------K--VHRLQVCEIRPR-DQGEYRIVA-KEK-DA

RAKLEL--------

>default|Q61S53_CAEBR/16591750.0|Q61S53_CAEBR/16591750

PQ--F-IEP----LESLERIEF-QPAHFQTKVTPQT--D-P--NLR-I-Q

---WFK-D-----GQPLR-N------------------------------

---S---N-R--FK--L--T-T--D--F----------------------

------------------G--YISLDIAHTIPE-DSGVYSVKA-SNA-KG

EAEVQAQLTV----

>default|Q61S53_CAEBR/24502542.0|Q61S53_CAEBR/24502542

PRF-I-QQL----SSPGDLVET-QPAHFEATVEPVD--D-P--TLT-I-S

---WFL-N-----GQPMS-A------------------------------

---S---S-R--VK--M--I-N--D--F----------------------

------------------G--WVIMDIAQTEPR-DSGEWKCVA-KNA-AG

EASSTATINV----

>default|Q2LYU8_DROPS/605696.0|Q2LYU8_DROPS/605696

PQ--F-IIP----LQNVQQTEG-GRVHMEARIEPVG--D-P--TMV-V-E

---WYL-N-----GRPLA-A------------------------------

---S---A-R--AT--S--V-F--K--F----------------------

------------------G--FIALDLLSIMGH-DSGEYMCRV-SNA-SG

VAESRAILSV----

>default|TITIN_HUMAN/43834472.0|TITIN_HUMAN/43834472

P-----VIK----RKIEPLEVA-LGHLAKFTCEIQS--A-P--NVR-F-Q

---WFK-A-----GREIY-E------------------------------

---S---D-K--CS--I--R-S--S--K----------------------

------------------Y--ISSLEILRTQVV-DCGEYTCKA-SNE-YG

SVSCTATLTV----

>default|MYLK_CHICK/429518.0|MYLK_CHICK/429518

P-----QFE----SRPQSLEAS-EGQEIKFKSKVSG--K-P--KPD-V-E

---WFK-E-----GVPIK-T------------------------------

---G---E-G--IQ--I--Y-E--E--D----------------------

------------------G--THCLWLKKACLG-DSGSYSCAA-FNP-RG

QTSTSWLLTV----

>default|Q17HV9_AEDAE/40874176.0|Q17HV9_AEDAE/40874176

P-----KIV----TPLRDMVVK-AGLIFHLDVNFTG--E-P--APE-V-V

---WTL-N-----GKPVE-S------------------------------

---D---K-R--TT--M--T-A--V--G----------------------

------------------H--HTIVHTVNCNRG-DSGDYKLKI-SNS-SG

TDEGSLHLTV----

>default|Q4RE90_TETNG/68286918.0|Q4RE90_TETNG/68286918

--V-D-LGI----NMKNLIVVK-AGENVFLDAEVFG--K-P--LPK-V-T

---WKR-D-----GIPLV-L------------------------------

---A---E-G--MK--M--V-Q--K--K----------------------

------------------H--VHLLELYSVTRK-ETGDYTILA-ENI-NG

SKYATIKVKV----

>default|Q0V960_BRARE/1094.0|Q0V960_BRARE/1094

----S-AFG----KKPKSQTAE-IGARVIF-EAETE--K-P--GVK-V-K

---WQR-E-----SKDIL-P------------------------------

---S---H-K--YT--I--S-A--E--D----------------------

------------------N--KHSLTINNIIQE-DAVGYAVIA-GMS-KV

KFELKI--------

>default|Q4SLN8_TETNG/507591.0|Q4SLN8_TETNG/507591

----V-FFR----KELKNQDAV-EGDDVSL-RCELS--K-P--GIR-V-E

---WRK-G-----GMVIQ-P------------------------------

---S---K-K--YE--M--K-Q--K--G----------------------

------------------C--VQELCIQNLVSE-DSGYYTCDA-GDQ-LT

TAALAV--------

>default|Q61SF4_CAEBR/65546642.0|Q61SF4_CAEBR/65546642

----L-RVA----KHLAGEVVK-TGEAVTF-EARIE--G-T--PDE-I-L

---WMR-N-----GQELT-D------------------------------

---G---E-K--TS--I--S-Q--N--G----------------------

------------------D--TLLLTVNSAEAS-DAGHYQLEV-RSK-GT

NIVSVASLVV----

>default|OBSL1_HUMAN/12101.0|OBSL1_HUMAN/12101

P-----CFL----RFPRPVRVV-SGAEAELKCVVLG--E-P--PPV-V-V

---WEK-G-----GQQLA-A------------------------------

---S---E-R--LS--F--P-A--D--G----------------------

------------------A--EHGLLLTAALPT-DAGVYVCRA-RNA-AG

EAYAAAAVTV----

>default|A2AAJ9_MOUSE/998.0|A2AAJ9_MOUSE/998

P-----RFL----TRPKAFVVS-VGKDATLSCQIVG--N-P--TPH-V-S

---WEK-D-----RQPVE-A------------------------------

---G---A-R--FR--L--A-Q--D--G----------------------

------------------D--VYRLTILDLALG-DSGQYVCRA-RNA-IG

EAFAAVGLRV----

>default|Q7ZZ48_BRARE/53365422.0|Q7ZZ48_BRARE/53365422

----V-TLL----KDITDQTTQ-LNKEAVF-ECEIKVNY-P--EIT-L-A

---WYK-D-----TQKLE-T------------------------------

---N---D-K--YD--I--K-L--V--G----------------------

------------------D--RQILKIKNCQTS-DQGNYRVVC-GPH-IS

SARLTV--------

>default|OBSCN_HUMAN/26472731.0|OBSCN_HUMAN/26472731

----V-VFL----KALDDLSAE-ERGTLAL-QCEVS--D-P--EAH-V-V

---WRK-D-----GVQLG-P------------------------------

---S---D-K--YD--F--L-H--T--A----------------------

------------------G--TRGLVVHDVSPE-DAGLYTCHV-GSE-ET

RARVRV--------

>default|Q4RU89_TETNG/25082592.0|Q4RU89_TETNG/25082592

P-----FFK----EELQSLEVE-EGGAASL-SCVLS--K-P--GVS-V-R

---WKK-N-----KLVLR-S------------------------------

---S---R-K--YE--M--K-Q--D--G----------------------

------------------C--LLQLQIKGLKPE-DAGSYSCHA-GDA-ET

TAMVAV--------

>default|A2AT70_MOUSE/26482732.0|A2AT70_MOUSE/26482732

P-----AWE----RHLQDVTLK-EGQTCTM-TCQFS--V-P--NVK-S-E

---WFR-N-----GRVLK-P------------------------------

---Q---G-R--VK--T--E-V--E--H----------------------

------------------K--VHKLTIADVRAE-DQGQYTCKH-EDL-ET

SAELRI--------

>default|Q9VR25_DROME/119212.0|Q9VR25_DROME/119212

----I-TFT----ENATVMTVK-EGEKATILCEVKG--E-P--QPN-V-T

---WHF-N-----GQPIS-A------------------------------

---G---A-A--DD--S--K-F--R--I----------------------

------------------L--ADGLLINKVTQN-DTGEYACRA-YQV-NS

IASDMQERTVlmki

>default|Q6KDZ1_CHICK/30983180.0|Q6KDZ1_CHICK/30983180

----L-AVR----VTPGSLVRG-VGSTAEF-ACTAS--D-P--RVH-L-E

---WLK-D-----GGELP-P------------------------------

---R-----------------H--S--V----------------------

------------------Q--DGVLRIAELAQG-AQGVYVCRA-STL-HG

QVEDRATLTV----

>default|Q20CA1_DROVI/65986687.0|Q20CA1_DROVI/65986687

P-----TFV----KFPVSVSVT-EGENATF-ECESD--G-E--LIN-L-A

---WLK-D-----GKPID-E------------------------------

---A---RAR--YS--F--T-K--D--S----------------------

------------------N--RYKFSVNKCTLD-DVGQYQARA-VSK-KG

ESLCAFSVNV----

>default|Q5BJ14_BRARE/221306.0|Q5BJ14_BRARE/221306

-E--A-FLR----RLESAYSVN-KGKKIVM-SVEVA--D-P--NAE-V-K

---WLR-N-----GQEIK-P------------------------------

---S---A-K--YI--M--E-A--D--G----------------------

------------------N--IRTLTINKCSLA-DDAAYECVA-GTD-KC

FTEVFV--------

>default|Q1JPV5_BRARE/227312.0|Q1JPV5_BRARE/227312

-D--A-FLK----KLDPAYSVE-KGKKIQL-SVELT--D-P--NAQ-V-K

---WLK-N-----GQEIK-P------------------------------

---S---A-K--YV--F--E-S--V--G----------------------

------------------G--IRKLTINKCTLA-DDAAYECVV-GEE-KS

FTEVFI--------

>default|A2AAJ9_MOUSE/38823965.0|A2AAJ9_MOUSE/38823965

----A-KFK----DSLKNEEAT-EGTTATL-SCELS--K----AAP-V-T

---WKK-G-----PKTLQ-S------------------------------

---G---D-K--YV--L--R-Q--D--G----------------------

------------------A--VCGLQIHGLTMA-DAGEYSCVC-GQE-KT

SATLTV--------

>default|Q4SLN8_TETNG/146230.0|Q4SLN8_TETNG/146230

----V-LFK----TQLQNLERQ-AGESASL-CCETT--K-P--GAS-V-V

---WRH-G-----DKVLS-S------------------------------

---S---S-K--YR--L--R-Q--D--G----------------------

------------------S--TVELVVYKLQGP-DAGVYSCDT-GSQ-RT

SAVLTV--------

>default|Q95YM2_PROCL/36913783.0|Q95YM2_PROCL/36913783

PRF-V-TNL----EDINDLVEG-QPAHFDCRVEPVG--D-P--TMR-I-E

---WFH-N-----GMPLA-A------------------------------

---G---S-R--VH--M--M-D--D--F----------------------

------------------G--FVVLDLEWTFAR-DTGEYICRA-TNK-WG

QATTKAMLNV----

>default|Q8BUJ6_MOUSE/697.0|Q8BUJ6_MOUSE/697

P-----MFT----QPLQSVVVL-EGSTATFEAHVSG--S-P--VPE-V-S

---WFR-D-----GQVIS-T------------------------------

---S---T-LpgVQ--I--S-F--S--D----------------------

------------------G--RARLMIPAVTKA-NSGRYSLRA-TNG-SG

QATSTAELLV----

>default|Q90933_CHICK/329417.0|Q90933_CHICK/329417

P-----YWV----RRPQSGVFG-PGETARLDCEVGG--K-P--RPQ-I-Q

---WSI-N-----GVPID-A------------------------------

---L---P-G--AE--R--R-W-----L----------------------

------------------R--GGALVLPELRPN-DSAVLQCEA-RNR-HG

RLLANAFLHV----

>default|Q7ZZ46_BRARE/64026492.0|Q7ZZ46_BRARE/64026492

--I-E-VDV----KLIEGLVVK-AGSTILLPAVMLG--I-P--TPT-A-K

---WQS-E-----GNELK-T------------------------------

---E---G-K--FK--I--V-T--E--G----------------------

------------------N--STTLTISECART-DSGEYSLTV-SNP-AG

SKSVALHVTV----

>default|Q4SM88_TETNG/18321916.0|Q4SM88_TETNG/18321916

----V-SIV----KPLRDRTAL-EKHRVIL-ECTVS--S-A--RCC-A-T

---WYK-D-----GKEVV-P------------------------------

---S---D-R--VE--I--L-D--D--G----------------------

------------------C--SLKLVIQEVAVE-DEGTYSVEV-GEH-TS

KAKLMV--------

>default|A2AAJ8_MOUSE/144228.0|A2AAJ8_MOUSE/144228

----V-TLL----RPLRDKIAM-EKHRGVL-ECQVS--R-A--SAQ-V-R

---WFK-G-----GVELQ-S------------------------------

---G---P-K--YE--V--V-S--D--G----------------------

------------------L--YRKLVINDVQPE-DEDTYTCDA-GNV-KT

SAQFFV--------

>default|Q4RE92_TETNG/93969485.0|Q4RE92_TETNG/93969485

P-----TFP----KKITSLQQT-EGQPIRFECRIAG--S-S--PIE-V-S

---WLR-D-----GEPLK-A------------------------------

---G---G-E--FS--M--L-Y--D--D----------------------

------------------N--TAVLQIERGEMR-HSGEYTCVA-TNS-VG

STSCRAKLTL----

>default|Q7PT04_ANOGA/33233415.0|Q7PT04_ANOGA/33233415

PVF-T-TPL----SNIDSLREG-ESAHFEAKLTPTD--D-P--KLK-V-E

---WFW-N-----GKPLK-T------------------------------

---G---S-R--FR--T--F-C--D--F----------------------

------------------G--FVILEISPVYPE-DSGEYSCRA-TNE-YG

EAVTTSTMRV----

>default|UNC89_CAEEL/23672456.0|UNC89_CAEEL/23672456

P-----KFT----TNMDDRQVK-EGEDVKFTANVEG--Y-P--EPS-V-A

---WTL-N-----GEPVS-K------------------------------

---H---P-N--IT--V--T-D--K--D----------------------

------------------G--EHTIEISAVTPE-QAGELSCEA-TNP-VG

SKKRDVQLAV----

>default|Q4RE92_TETNG/55825704.0|Q4RE92_TETNG/55825704

P-----KFI----IPLTNITAA-AGTPVILQCLVSG--T-P--NPT-A-E

---WYK-D-----GGRVT-Dsrciiqektvghfnllitnpvvqwshngkv

itsS---S-V--YK--L--I-E--E--R----------------------

------------------E--DYTLVITKVTSE-YEGEYSCTA-TNR-FG

QTTCTTYLEV----

>default|P79757_CHICK/734823.0|P79757_CHICK/734823

P-----AFI----TQPKSQNVN-EGQDVLFTCEVSG--D-P--SPE-V-E

---WLR-N-----NQPIA-V------------------------------

---S---S-H--MR--A--T-R--S--K----------------------

------------------N--TYSLEIRNAAVS-DTGKYTVKA-KNY-HG

QCSATASLTV----

>default|Q7PT04_ANOGA/46224711.0|Q7PT04_ANOGA/46224711

------PKL----SEMKDCLVE-EGSPAQFKTTIVG--KpT--KMT-V-Q

---WLR-E-----GLLIP-E------------------------------

---S---P-D--FQ--M--I-N--E--G----------------------

------------------N--NAVLLIGTTYEE-DSGTFTCRV-TTS-AG

QVETSAKLIV----

>default|Q61GU4_CAEBR/270362.0|Q61GU4_CAEBR/270362

---PT-VVD----PPQTNLQVP-RGSTFSLTCKAVA--V-P--EPY-I-N

---WRL-N-----WGPVC-E------------------------------

---P---P-R--CL--Q--T-S--E--G----------------------

------------------G--YGTLTIHDAQPV-DQGAYTCEA-INV-KG

RVLATPDCIVrv--

>default|Q4RU89_TETNG/683769.0|Q4RU89_TETNG/683769

----V-FIL----DELEDVECL-EGDTVTFRCRICP--S-D--YSE-V-K

---WYL-D-----ETLLY-T------------------------------

---N---D-L--NE--I-QL-L--A--G----------------------

------------------G--YHVLTFRQLARK-DTGTISFSA-GDK-RS

YASLLV--------

>default|Q9TXK2_CAEEL/33143406.0|Q9TXK2_CAEEL/33143406

PAI-V-NAL----QVQGALEEG-GSAHLQTQFTPVA--D-P--SIK-V-E

---WLK-D-----GQPIF-H------------------------------

---S---N-R--YK--M--V-H--D--F----------------------

------------------G--FAVLDILHLLKH-DAGEYTFRV-SNN-SG

EASTSTSFEV----

>default|Q95YM2_PROCL/15761667.0|Q95YM2_PROCL/15761667

PV--F-TTS----LKNIEILEG-QRAHFEVRLIPTS--D-P--TMK-V-E

---WFH-N-----NVPVK-S------------------------------

---G---S-R--FT--E--Y-N--D--F----------------------

------------------G--FVALDIMYAYAE-DSGTYTCRA-TNV-IG

QAVSSCNLVC----

>default|Q4TAD4_TETNG/30693158.0|Q4TAD4_TETNG/30693158

P-----SFV----KALVPVAAV-VGAPLHLHCQVDL--D-T--GVT-V-T

---WTK-D-----GRKVH-Q------------------------------

---S---P-D--CK--L--F-F--E--N----------------------

------------------K--MVSLDILKATLR-DCGTYVCTV-ANE-AG

SATCSAEVKV----

>default|A2AAJ9_MOUSE/35283612.0|A2AAJ9_MOUSE/35283612

----S-IFS----RPLTDVTVT-EGEDLTL-VCETT--T-V--DSS-V-R

---WTK-D-----GKTLR-P------------------------------

---S---A-R--CQ--L--S-H--E--G----------------------

------------------C--QAQLLITATTPQ-DGGRYKCEI-GGA-SS

SSIVRV--------

>default|Q8ISF7_CAEEL/30033088.0|Q8ISF7_CAEEL/30033088

----A-NIL----TRPETQFVM-EGQPTVI-TVELD--D-P--NVI-V-N

---WLK-D-----RRPLH-E------------------------------

---N---E-R--IR--L--E-T--D--G----------------------

------------------Q--GNHRVIIPNTCV-GDQGTYLALTSSE-S-

---VAITLVV----

>default|Q95YM2_PROCL/23712463.0|Q95YM2_PROCL/23712463

PVF-T-QPL----QNLDNLTEG-ETAHFECRLIPVG--D-P--NLK-V-E

---WFR-N-----EKPIE-T------------------------------

---S---S-R--IN--R--T-H--D--F----------------------

------------------G--YVALDISGVRPD-DEGIYMCRA-YND-LG

EAVTTASIKI----

>default|Q2LYU8_DROPS/27422833.0|Q2LYU8_DROPS/27422833

PV--F-ITH----LNNIECKES-DNVRFECNVEPAR--D-P--TMQ-I-E

---WFY-N-----GQPLQ-A------------------------------

---A---A-K--FK--S--I-Y--D--F----------------------

------------------G--YCALDLNNSYAE-NSGIYTCKA-TNS-RG

SATTSGTLKC----

>default|Q4S5G7_TETNG/209303.0|Q4S5G7_TETNG/209303

P-----AIH----TGPAQQAVF-VGETASFLCEVSG--K-P--RPE-I-T

---WEK-QlkgkeNTVMR-P------------------------------

---N---H-V--RG--N--I-V--V--T----------------------

------------------N--IGQLVIYNAQLH-DAGIYTCTA-RNV-GG

GVSSHFPLAV----

>default|Q4RJ20_TETNG/390.0|Q4RJ20_TETNG/390

-------IQ----GEELNATVM-LGGPVVLHCQSDA--I-P--PPT-L-S

---WRK-D-----GRPLF-R------------------------------

---K---P-G--LT--V--L-S--D--G----------------------

------------------S--V--LRVDSAQVQ-DSGRYSCEA-TNV-AG

KTEKNYNLNV----

>default|Q5RIP6_BRARE/20132100.0|Q5RIP6_BRARE/20132100

P-----NIM----GEEVNNTVL-MGQSVQLHCQSDA--I-P--PPA-L-S

---WRK-D-----GRPLY-R------------------------------

---K---P-G--LS--V--S-E--D--G----------------------

------------------S--F--LKIISAQVQ-DTGRYTCEA-TNV-AG

KTEKHYNLNV----

>default|Q7Q767_ANOGA/17241814.0|Q7Q767_ANOGA/17241814

P-----HFI----KPIRGLTVE-PGENAKF-TVQVD----E--MSN-M-Q

---WLK-D-----NKPLD-D------------------------------

---R---LAD--RV--I--T-K--E--N----------------------

------------------DDlTYSLEIQHCREQ-DSGIYTARA-ING-TE

NASCTAQLIV----

>default|Q17HV9_AEDAE/14241515.0|Q17HV9_AEDAE/14241515

---PR-IDR----TNLKPIVVR-GGKMVHYDVNVKG--E-P--EPE-I-T

---WVL-K-----EKPVF-A------------------------------

---D---D-H--YE--I--I-N--K--D----------------------

------------------Y--NTKFTIKDSMRSkHSGVYEIIA-KNE-HG

MDKEKVEITV----

>default|Q4SK55_TETNG/12871371.0|Q4SK55_TETNG/12871371

----V-NIV----KPLEDKTAF-EMSRVIL-DCTVS--N-P--RCS-I-R

---WYK-G-----CTVIL-P------------------------------

---S---E-R--FE--I--C-S--E--G----------------------

------------------G--YRKLIIQQVALD-DAGTYSVQV-GEY-SC

SAKLTV--------

>default|Q8ISF7_CAEEL/1528315372.0|Q8ISF7_CAEEL/1528315372

P-----KVE----AEQSIVEVK-VGDVAKL-SAKIS--E-P--ASS-V-N

---WTK-D-----DKPIK-E------------------------------

---D---G-N--VK--AQLS-P--D--G----------------------

---------------------TAQLTISKTDSA-HSGIYKLNV-END-AG

KGKVEIALRI----

>default|Q17HV9_AEDAE/20172109.0|Q17HV9_AEDAE/20172109

---PR-IDR----KNLDKKVLR-FGQLLNVEADVTG--E-P--APQ-I-T

---WEL-K-----GKPIT-G------------------------------

---K---E-D--ER--I--R-L--DneD----------------------

------------------Y--KTNLIIRNLKRS-DSGIYKVTA-KNK-NG

TDTVEMELVV----

>default|A2ASS5_MOUSE/21342222.0|A2ASS5_MOUSE/21342222

--I-T-FTQ----ELQDVVAKE-KDTMATF-ECETS--E-P--FIK-V-K

---WYK-D-----GIEVH-A------------------------------

---G---D-K--YR--M--H-S--D--R----------------------

------------------K--VHFLSVLTIDTS-DAEDYSCVL-VED-EN

IKTTAKLIV-----

>default|Q4RE92_TETNG/85838672.0|Q4RE92_TETNG/85838672

P-----SFV----SPPEPQSAV-PNAKVRFKGTFKG--T-P--PFT-V-Q

---WFK-D-----DRELM-T------------------------------

---G---P-T--CF--T--G-L--D--G----------------------

------------------L--SCFLELYSVNIS-QSGVYSCQV-SNA-AG

SARCSANLTV----

>default|SPEG_RAT/727816.0|SPEG_RAT/727816

P-----VFE----IPLQNMVVA-PGADVLLKCIITA--N-P--PPQ-V-S

---WKK-D-----GSVLH-S------------------------------

---E---G-R--LL--I--R-A--E--G----------------------

------------------E--RHTLLLREAQAA-DAGSYTATA-TNE-LG

QASCASSLAV----

>default|Q8NDA2_HUMAN/12061292.0|Q8NDA2_HUMAN/12061292

P-----VFQ----VEPQDMTVR-SGDDVALRCQATG--E-P--TPT-I-E

---WLQ-A-----GQPLR-A------------------------------

---S---R-R--LR--T--L-P--D--G----------------------

------------------S--L--W-LENVETG-DAGTYDCVA-HNL-LG

SATARAFLVV----

>default|Q8T104_BOMMO/259349.0|Q8T104_BOMMO/259349

--L-R-YPR----QYEDGLLYE-INETIFLKTTIVG--K-P--TPT-I-E

---WRH-D-----GHPII-T------------------------------

---D---D-R--IE--I--S-I--T--P----------------------

------------------K--FSVLKIHSAKRS-DRGEYQIQA-KNI-IG

EDTAAFLVTV----

>default|Q4RU89_TETNG/271355.0|Q4RU89_TETNG/271355

----A-VFL----KSLEDAVGE-EEGEVCL-HCEIS--K-E--TAT-P-V

---WRK-D-----GSVLT-A------------------------------

---D---D-K--HE--F--L-Q--F--G----------------------

------------------K--ARALIIRSLSKD-DAGLYTCDF-GTS-QT

KAKVTV--------

>default|Q4RE92_TETNG/43514440.0|Q4RE92_TETNG/43514440

P-----VFV----KEMCPLKAR-VGEMSEFTCQFHG--D-P--LPT-V-T

---WLK-D-----GHPLT-N------------------------------

---N---P-D--FD--I--I-D--R--A----------------------

------------------N--RSKLTVFYPTAD-HEGTYDCII-TNK-HG

KSLCSATLEI----

>default|A2AT18_MOUSE/12401329.0|A2AT18_MOUSE/12401329

a---T-IMEEA---VSIDVTQG-DPATLQV-KFSGT--K----EIS-A-K

---WFK-D-----GQELT-L------------------------------

---G---P-K--YK--I--S-V--T--D----------------------

------------------T--VSILKIISTEKK-DSGEYTFEV-QND-VG

RSSCKASINV----

>default|A2AJ76_MOUSE/27842872.0|A2AJ76_MOUSE/27842872

--------EEAqgRVTEYREIV-ENNPAYLYCDTNA--I-P--PPE-L-T

---WYR-E-----GQPLS-A------------------------------

---A---D-G--VS--V--L-Q--G--G----------------------

------------------R--I--LQLPLVQAE-DAGRYSCKA-ANE-VG

EDWLHYELLV----

>default|UNC89_CAEEL/25632652.0|UNC89_CAEEL/25632652

P-----AFQ----SDIAPINLT-EGDTLECKLLITG--D-P--TPF-V-K

---WYI-G-----TQLVC-A------------------------------

---T---E-D--TE--I--S-N--A--N----------------------

------------------G--VYTMKIHGVTAD-MTGKIKCVA-YNK-AG

EVSTEGPLKV----

>default|UNC89_CAEEL/46834772.0|UNC89_CAEEL/46834772

----I-TFL----KPLKDQSIT-EGENAEF-SVETN--T-K--PRI-V-K

---WYK-N-----GQEIK-P------------------------------

---N---S-R--FI--I--E-Q--K--T----------------------

------------------D--TKYQLVIKNAVR-DDADTYKIVLENT-AG

EAESSAQLTV----

>default|A2AAJ9_MOUSE/22422326.0|A2AAJ9_MOUSE/22422326

----A-KFS----KPLEPVEGE-LGGTVML-VCELS--P-E--QAE-V-V

---WRC-G-----NTQLR-P------------------------------

---G---K-R--FQ--M--T-S--E--G----------------------

------------------P--RRTLTVSGLRED-DAEEYVCES-RDD-RT

SARLTV--------

>default|A2AAJ9_MOUSE/42184301.0|A2AAJ9_MOUSE/42184301

P-----RFI----EDLRSQEAT-EGTMATL-RCQMS--K----AAP-V-E

---WRK-G-----SETLG-D------------------------------

---G---G-R--YS--L--R-Q--N--G----------------------

------------------A--VCELQIHDLAVE-DTGEYSCVC-GQE-KT

SATLNV--------

>default|A2AT70_MOUSE/830919.0|A2AT70_MOUSE/830919

----A-TFV----KRLADTSVE-TGSPIVLEATYSG--T-P--PIS-V-S

---WMK-N-----EYPLS-Q------------------------------

---S---P-N--CG--I--T-T--T--E----------------------

------------------K--SSILEILESTIE-DYAQYACLI-ENE-AG

QDICEALVSV----

>default|Q9TXK2_CAEEL/45714660.0|Q9TXK2_CAEEL/45714660

P-----DFS----ARLNDLRIQ-QNGPAEFSCQIGG--E-P--KPT-I-Q

---WFK-D-----GQPLP-N------------------------------

---D---D-R--FQ--V--V-E--E--G----------------------

------------------G--AYKLKFSNIIST-DAGIYEIVA-KNG-VG

EARCKARLNV----

>default|Q4SLP0_TETNG/31121.0|Q4SLP0_TETNG/31121

P-----DFD----EILANCTAE-LGQTVKLACKRTG--V-P--KPS-I-I

---WYK-D-----GRPVG-A------------------------------

---D---P-H--HI--I--I-E--D-PD----------------------

------------------G--SCTLILDNMSAD-DSGQYMCFA-TSS-AG

NATTLGKVTV----

>default|A0S6W8_SPOFR/116204.0|A0S6W8_SPOFR/116204

PK--I-VSR----PTTTYQSTE-GDATLKIPCVAVG--D-P--KPT-I-T

---WKM-D-----GRPIA-V------------------------------

---P---S-S--KY--A--V-E--D--G----------------------

------------------A--L--I-IKYPTVT-DTKSYECVA-TNE-AG

SVTTTFKTFI----

>default|A2AJ76_MOUSE/28762967.0|A2AJ76_MOUSE/28762967

PVIPG-DTQ----ELVEEVTVN-ASSAVSLECPALG--N-P--APA-V-S

---WFQ-N-----GLPVS-P------------------------------

---S---P-R--LQ--V--L-E--E--G----------------------

------------------Q--V--LKVATAEVA-DAASYMCVA-ENQ-AG

SAEKLFTLKV----

>default|Q5RIP6_BRARE/29683050.0|Q5RIP6_BRARE/29683050

--------------LPVVLNVQ-VGKSVMLECESNA--V-P--PPV-I-T

---WYK-N-----GRPIA-E------------------------------

---T---A-N--LR--I--L-A--D--G----------------------

------------------Q--M--LQLKETEVS-DTGQYVCKA-TNV-AG

QVDKNFHLNI----
