# Supplementary figures and images for: Mechanical Network in Titin Immunoglobulin from Force Distribution Analysis
Source: PLoS Comput Biol. 2009 Mar 13;5(3):e1000306. doi: 10.1371/journal.pcbi.1000306 (PMC2643529; doi:10.1371/journal.pcbi.1000306)

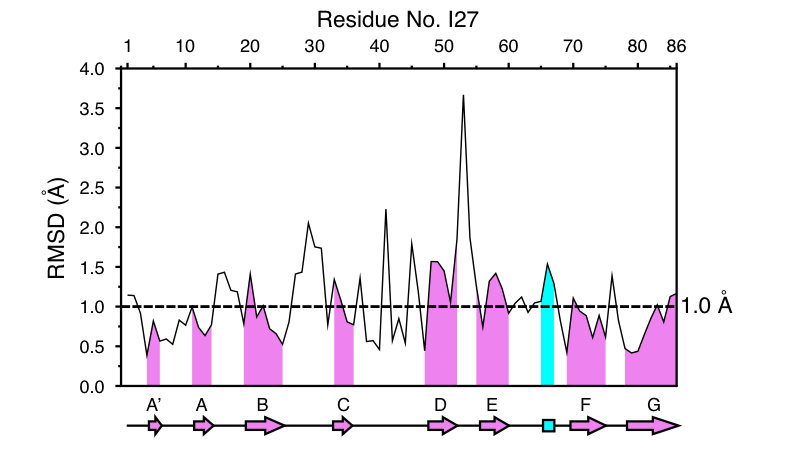

Supplement: Figure S1 — Root mean square distance between Cα carbons of the NMR ensemble (1TIT) and the newly determined crystal structure of I27. (0.07 MB TIF) [file pcbi.1000306.s003.tif]

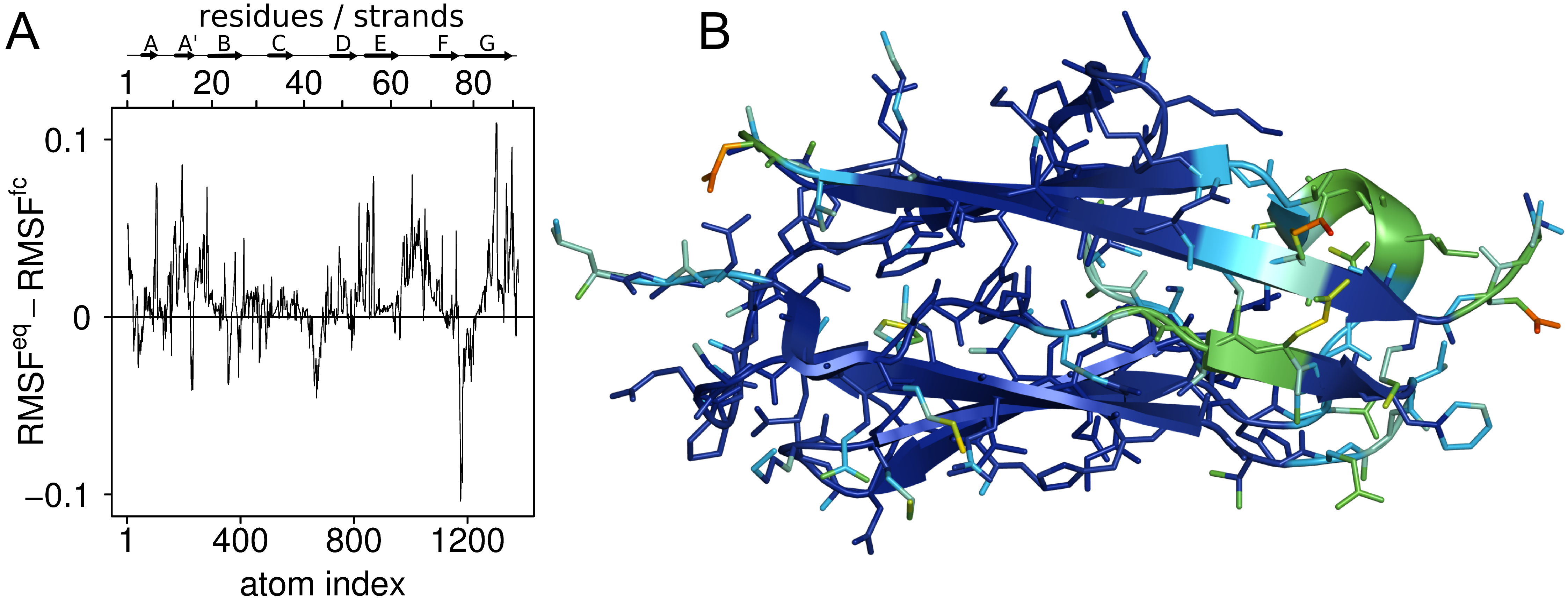

Supplement: Figure S2 — Stiffening of force bearing parts under load in I27 indicated by decreased root mean square fluctuations (RMSF). The observed decrease in RMSF corresponds well with the force distribution pattern. (A) Differences in RMSF between equilibrium and force clamp simulations plotted along the protein sequence. (B) RMSF differences color coded on the I27 structure. Colors range from blue for no change to red for high change. Data are averages over 10 equilibrium and 8 FCMD simulations, with 20 ns simulation time each. (2.75 MB TIF) [file pcbi.1000306.s004.tif]

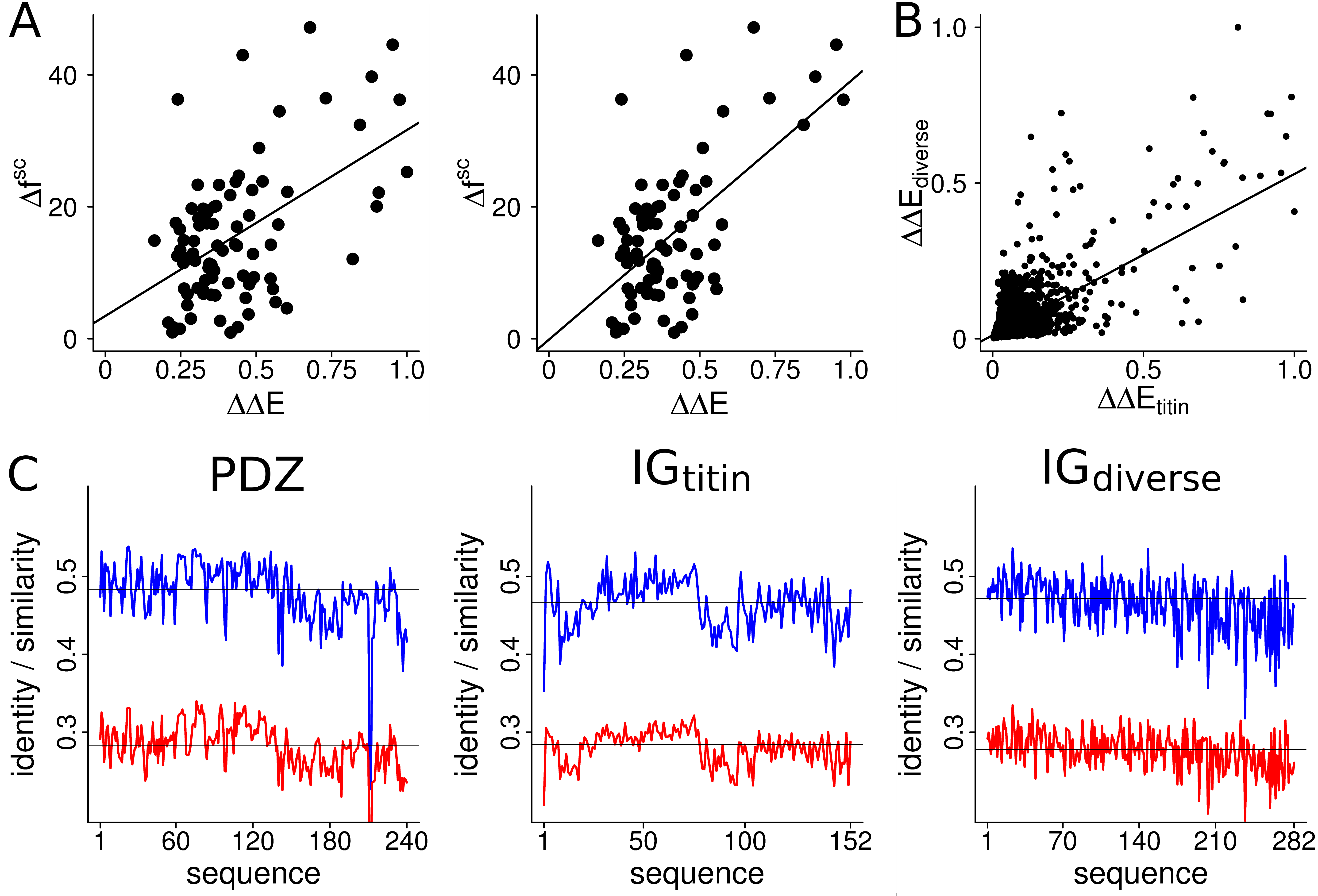

Supplement: Figure S3 — Correlation of the evolutionary and mechanical network and quality assessment for the multiple alignments. (A) Correlation of evolutionary ΔΔE values with inter side-chain forces Δfsc. Plotted are ΔΔE versus Δfsc including (left) and excluding (right) interaction interface residues, yielding correlation coefficients of R = 0.52 and R = 0.60. The lines show the fit of a linear model to the data. (B) Correlation between pair-wise statistical coupling ΔΔE values for IGtitin and IGdiverse, yielding a correlation coefficient of R = 0.65. The line shows the fit of a linear model to the data. (C) The average sequence similarity for IGtitin and IGdiverse is comparable with the PDZ alignment published by Ranganathan and co-workers. (0.78 MB TIF) [file pcbi.1000306.s005.tif]

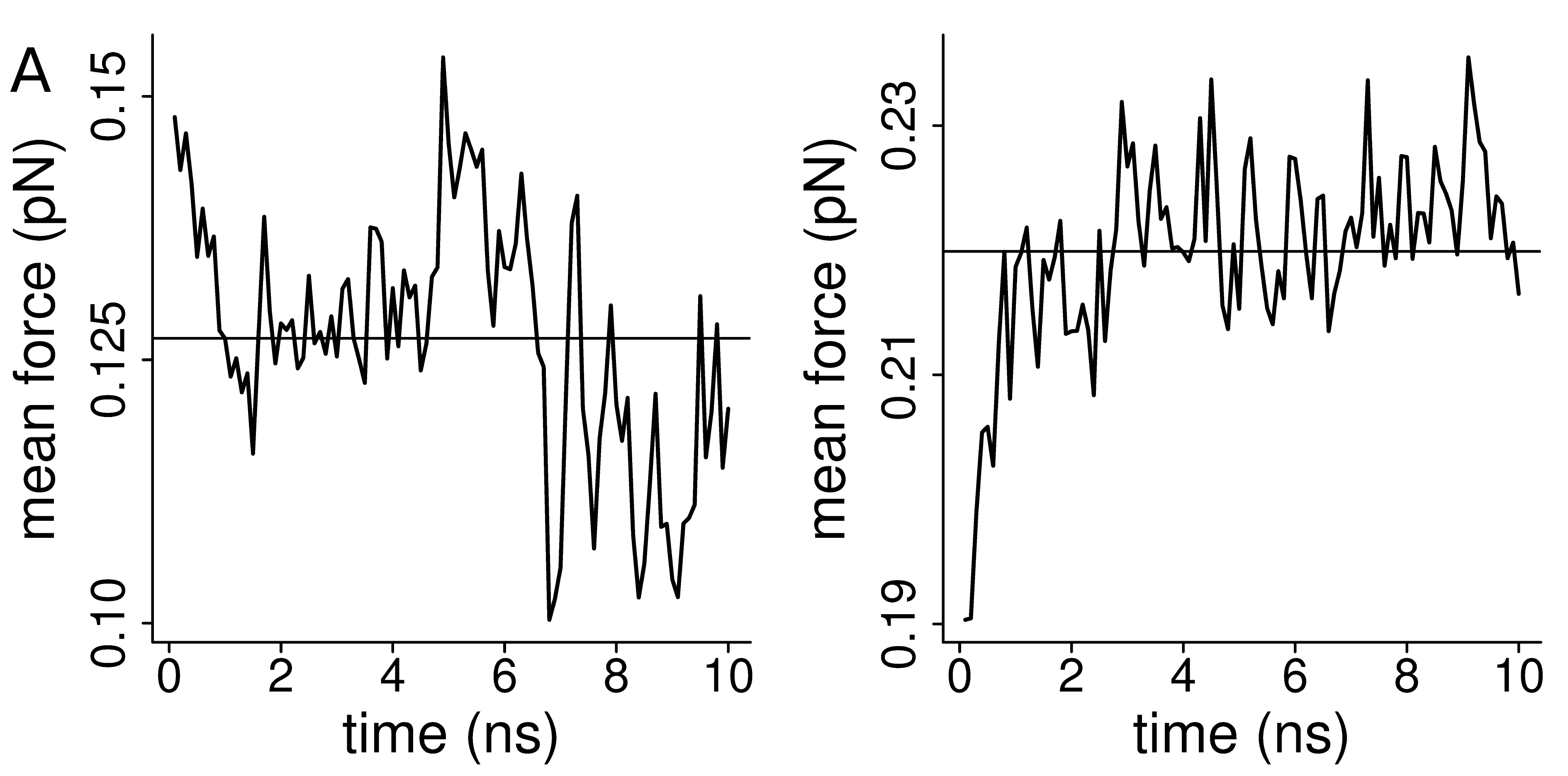

Supplement: Figure S4 — Speed of signal propagation in I27, shown are forces averaged over all atom pairs in I27 during equilibrium (left) and FCMD simulations (right). Each data point corresponds to the average force during 100 ps. The global means are plotted as black lines. (0.27 MB TIF) [file pcbi.1000306.s006.tif]

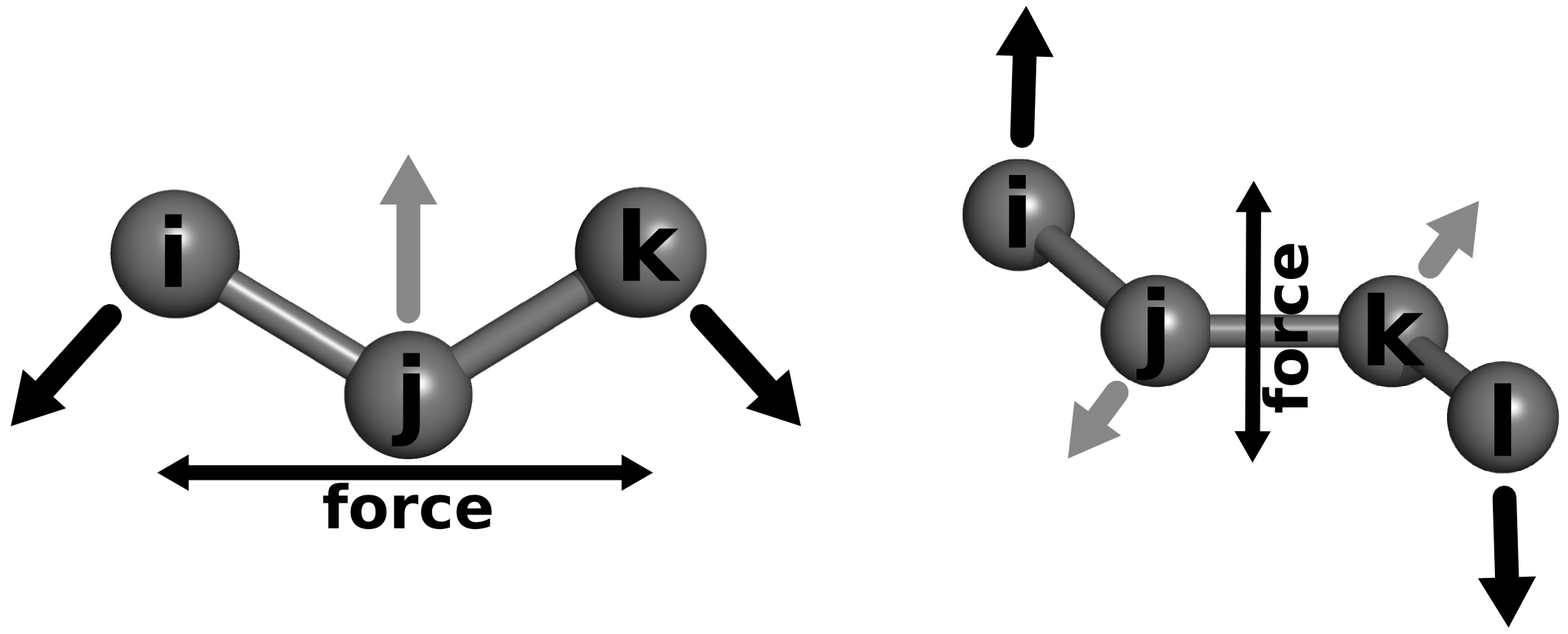

Supplement: Figure S5 — Approximations used for angle and dihedral terms. The OPLS force field uses angle and dihedral terms calculated as multibody forces between atoms i,j,k for angles and atoms i,j,k,l for dihedrals. For angles, Gromacs internally calculates the force vectors I,J,K acting on these atoms. As we cannot directly map such multibody forces to pairwise interactions, we represent angle bending as |K-I|, the force component acting in i,k direction. Similarly, for dihedral terms the force vectors I,J,K,L acting on the atoms i, j, k, l are calculated. To represent bending of dihedral angles we use |L-I|, the force component acting in direction i,l. This will not provide physically correct forces, but is sufficient to detect rearrangements under mechanical load. (0.25 MB TIF) [file pcbi.1000306.s007.tif]
