# Supplementary material for: Mechanical Network in Titin Immunoglobulin from Force Distribution Analysis
Source: PLoS Comput Biol. 2009 Mar 13;5(3):e1000306. doi: 10.1371/journal.pcbi.1000306 (PMC2643529; doi:10.1371/journal.pcbi.1000306)
Supplement: Table S1 — T-tests for unfolding forces of in silico mutants. (0.05 MB DOC) [file pcbi.1000306.s008.doc]

**Supplementary Table 1. T-tests for unfolding forces of *in-silico* mutants**

**t- and p-values from t-tests against wild-type I27 unfolding forces. Data were obtained from 15 independent unfolding simulations for the wild type and at least 10 independent simulations for *in-silico* mutants. We tested forces needed for transition to the intermediate and for rupture of the A’G strand that leads to complete unfolding.**

| **Mutation** | **t-value intermediate** | **p-value intermediate** |  | **t-value**  **A’G rupture** | **p-value**  **A’G rupture** |
| --- | --- | --- | --- | --- | --- |
| **I2A** | 5.5 | < 10^-4^ |  | -1.7 | 0.11 |
| **I23A** | 2.7 | 0.01 |  | 0.9 | 0.38 |
| **L25A** | 5.1 | < 10^-4^ |  | 0.3 | 0.74 |
| **V30A** | 0.8 | 0.46 |  | 0.1 | 0.89 |
| **L84A** | 3.5 | 0.002 |  | 3.4 | 0.002 |
| **L78A** | 0.9 | 0.38 |  | 1.3 | 0.22 |
| **F73A** | -0.7 | 0.47 |  | 0.2 | 0.82 |
| **F21A** | 2.1 | 0.04 |  | 3.0 | 0.006 |
| **V4A** | 4.9 | < 10^-4^ |  | 0.6 | 0.57 |
| **V13A** | 1.5 | 0.13 |  | 2.4 | 0.02 |
| **V86A** | 1.9 | 0.06 |  | 2.2 | 0.04 |
| **I49A** | 1.3 | 0.2 |  | 1.9 | 0.07 |
| **V71A** | 2.2 | 0.04 |  | 1.8 | 0.08 |
| **L41A** | 1.1 | 0.3 |  | 1.4 | 0.17 |
| **L58A** | 0.2 | 0.82 |  | 0.7 | 0.49 |
