# Supplementary material for: Mechanical Network in Titin Immunoglobulin from Force Distribution Analysis
Source: PLoS Comput Biol. 2009 Mar 13;5(3):e1000306. doi: 10.1371/journal.pcbi.1000306 (PMC2643529; doi:10.1371/journal.pcbi.1000306)
Supplement: Table S2 — Backbone and heavy atom root mean square deviation (RMSD) in Angstroem for I27 in silico mutants. (0.04 MB DOC) [file pcbi.1000306.s009.doc]

**Supplementary Table 2:** Backbone and heavy atom root mean square deviation (RMSD) in A for I27 *in-silico* mutants.

RMSD values were measured by comparing the equilibrated wild type structure with the mutant structures after 5 ns equilibration. All mutants remained remarkably stable with a backbone RMSD < 1.4 Å.

| Mutant | Backbone RMSD | Heavy atom RMSD |
| --- | --- | --- |
| I2A | 1.2 | 1.8 |
| V4A | 1.1 | 1.6 |
| V13A | 1.0 | 1.5 |
| F21A | 0.9 | 1.5 |
| I23A | 0.9 | 1.5 |
| L25A | 1.2 | 1.8 |
| V30A | 1.1 | 1.7 |
| I49A | 1.1 | 1.8 |
| L58A | 1.1 | 1.7 |
| V71A | 1.3 | 2.0 |
| F73A | 1.2 | 1.7 |
| L78A | 1.3 | 1.8 |
| L84A | 1.1 | 1.6 |
| V86A | 1.0 | 1.6 |
