# Supplementary material for: Mechanical Network in Titin Immunoglobulin from Force Distribution Analysis
Source: PLoS Comput Biol. 2009 Mar 13;5(3):e1000306. doi: 10.1371/journal.pcbi.1000306 (PMC2643529; doi:10.1371/journal.pcbi.1000306)
Supplement: Table S3 — X-ray refinement statistics. (0.05 MB DOC) [file pcbi.1000306.s010.doc]

**Supplementary Table 3.** X-ray refinement statistics.

R-factor = Σ*_hkl_* ||F_o_| - |F_c_|| / Σ*_hkl_* |F_o_|, where F_o_ is the observed structure factor amplitude and F_c_ the calculated structure-factor amplitude. R-free is calculated based on 7.2% of reflections not used in refinement. We use the abbreviations: RMS - root mean square. RMSD - root mean square distance. NCS - non-crystallographic symmetry.

| Space group | *P*2_1_2_1_2_1_ |
| --- | --- |
| Resolution range (Å) | 20.0-1.8 |
| R-factor (%) | 0.211 |
| R-free (%) | 0.268 |
| Asymmetric unit |  |
| Number of residues | 552 |
| Number of solvent atoms | 480 |
| Number of Zn atoms | 24 |
| RMSD bond length (Å) | 0.014 |
| RMSD bond angles (°) | 1.701 |
| Ramachandran plot |  |
| Core regions (%) | 98.7 |
| Disallowed regions (%) | 1.3 |
| Average B factors (Å^2^) |  |
| Main chain atoms | 27.3 |
| All atoms, including solvent | 28.8 |
| NCS |  |
| RMS difference for Cα atoms (Å) | 0.31 |
| RMS difference for all atoms (Å) | 0.94 |
