# Supplementary material for: Mechanical Network in Titin Immunoglobulin from Force Distribution Analysis
Source: PLoS Comput Biol. 2009 Mar 13;5(3):e1000306. doi: 10.1371/journal.pcbi.1000306 (PMC2643529; doi:10.1371/journal.pcbi.1000306)
Supplement: Table S4 — Crystallographic statistics, data processing and scaling. (0.04 MB DOC) [file pcbi.1000306.s011.doc]

**Supplementary Table 4.** Crystallographic statistics, data processing and scaling.

Values for the highest resolution shell are indicated between parentheses. R_merge_ = Σ*_h_*Σ*_i_* | I(h, i) - <I(h)> | / Σ*_h_*Σ*_i_* I(h, i); where I(h, i) is the *i*th intensity measurement of reflection h including symmetry-related reflections, and <I(h)> denotes the average.

| Beamline | BW7B (EMBL Hamburg) |
| --- | --- |
| Wavelength (Å) | 0.845 |
| Space group | *P*2_1_2_1_2_1_ |
| Unit cell dimensions (Å, °) | *a* = 62.1 *b* = 76.0, *c* = 134.3, α = β = γ = 90 |
| Resolution range (Å) | 20.0-1.80 (1.85-1.80) |
| Number of reflections (unique/total) | 53,298 (294,179) |
| R_merge_ (%) | 7.8 (18.0) |
| Multiplicity | 5.5 (5.6) |
| Completeness (%) | 89.2 (92.3) |
| Mean I/σ(I) | 11.3 (5.4) |
